# The neuropsychiatric systemic lupus erythematosus biomarker landscape: what blood and cerebrospinal fluid analytes have been studied and how they performed? A systematic review and meta-analysis

**DOI:** 10.3389/fimmu.2026.1903757

**Published:** 2026-10-01

**Authors:** Carolina Muñoz-Grajales, Sara Duaibes, Alan Cheng, Yasmin Nariman, Melanie Anderson, Dennisse Bonilla, Sindhu R. Johnson, Meggan Mackay, Joan Wither, Dafna D. Gladman, Zahi Touma

**Affiliations:** 1 Schroeder Arthritis Institute, Krembil Research Institute, University Health Network, Toronto, ON, Canada; 2 University of Toronto Lupus Clinic, Centre for Prognosis Studies in Rheumatic Diseases, Toronto Western Hospital, Toronto, ON, Canada; 3 Department of Internal Medicine, Rady Faculty of Health Sciences, University of Manitoba, Winnipeg, MB, Canada; 4 Library and Information Services, University Health Network, The Institute of Education Research, University Health Network, Toronto, ON, Canada; 5 Institute of Health Policy, Management and Evaluation, University of Toronto, Toronto, ON, Canada; 6 Division of Rheumatology, Department of Medicine, University of Toronto, Toronto, ON, Canada; 7 Institute of Molecular Medicine, The Feinstein Institutes for Medical Research, Manhasset, NY, United States; 8 Department of Immunology and Division of Rheumatology, University of Toronto, Toronto, ON, Canada; 9 Department of Medicine, University of Toronto, Toronto, ON, Canada

**Keywords:** autoantibodies, biomarkers, cerebrospinal fluid, cytokines, meta-analysis, neuroinflammation, neuropsychiatric systemic lupus erythematosus, systemic lupus erythematosus

## Abstract

**Objective:**

This study aims to evaluate biomarkers that discriminate systemic lupus erythematosus patients with and without neuropsychiatric involvement.

**Methods:**

We searched MEDLINE, Embase, and CENTRAL (inception—March 2024) for studies of blood or cerebrospinal fluid (CSF) analytes in neuropsychiatric systemic lupus erythematosus (NPSLE). Random-effects meta-analyses were conducted for analyte-biofluid pairs with ≥5 independent studies; the remaining data were synthesized narratively.

**Results:**

A total of 93 studies were included. Few analyte–biofluid combinations met the criteria for meta-analysis, and most promising biomarkers (intrathecal inflammation, neuronal injury, and reduced neuroprotection) were assessed in only small, heterogeneous studies. The meta-analyses showed higher CSF IL-6 and MCP-1 in NPSLE than non-NPSLE SLE (Hedges’ *g* 0.81 and 0.66, respectively), a smaller heterogeneous effect for IL-8, and two- to threefold higher odds of NPSLE in patients positive for serum anti-ribosomal P (OR 2.68, 95% CI 2.03–3.53), anti-NR2/NMDAR (2.09, 1.07–4.06), and IgG anticardiolipin (2.36, 1.56–3.59) antibodies. Compared with non-SLE controls, NPSLE generally showed higher CSF soluble mediators than healthy or non-inflammatory neurological comparators, but the levels in CNS infections were often similar or higher.

**Conclusion:**

CSF markers of intrathecal inflammation, neuronal injury, and reduced neuroprotection distinguish NPSLE from non-NPSLE SLE but are neither specific for NPSLE nor easily obtainable in routine practice. Classical serum autoantibodies (anti-ribosomal P, anti-NR2, and antiphospholipid antibodies) show modest, phenotype-dependent diagnostic performance. Evidence remains fragmented, with heterogeneous assays, cutoffs, and case definitions, underscoring the need for harmonized multicenter studies to identify robust diagnostic, evaluative, and prognostic biomarkers for overall and manifestation-specific NPSLE.

## Introduction

Neuropsychiatric (NP) manifestations of systemic lupus erythematosus (SLE) are collectively termed NPSLE and comprise at least 19 syndromes affecting the central and peripheral nervous systems. Estimates of NPSLE prevalence vary widely across studies (12% to 95%), in part due to heterogeneous case definitions, study design, and attribution rules (1). Nonetheless, considering studies with the stringent definitions and attribution, NPSLE occur in over 20% of patients (1, 2). NPSLE has a substantial negative impact on health-related quality of life (HRQoL) (3, 4), and, notably, is associated with increased mortality compared with both the general population and patients with SLE without neuropsychiatric involvement (4–6). Therefore, efforts to identify reliable diagnostic, evaluative, and prognostic biomarkers are of paramount importance.

Over the past decades, many analytes in blood and CSF have been studied as potential NPSLE biomarkers, ranging from autoantibodies (Ab) and soluble inflammatory mediators to extrapolated molecules from other neurological diseases reflecting general neuronal/glial injury or blood–brain barrier (BBB) dysfunction (7). However, the literature is hard to interpret as often the same analytes have been tested repeatedly with conflicting results across studies. Differences in case definitions, timing, phenotype granularity (overall vs. central/peripheral nervous system; focal vs. diffuse), biofluid choice (CSF, serum, and plasma), handling, assay platforms, and thresholds all contribute to these inconsistent findings. Previous systematic reviews have summarized the available evidence on biomarkers associated with NPSLE, highlighting the substantial heterogeneity across studies, biomarker assays, and clinical phenotypes as well as the need for further validation (Lindblom et al.) (7). The present systematic review provides an updated assessment of the literature through March 2024, incorporates quantitative meta-analyses where sufficient studies were available, maintains separate evaluations of CSF and blood biomarkers, pools continuous outcomes using Hedges’ *g*, and evaluates the robustness and generalizability of pooled estimates through sensitivity analyses and reporting of prediction intervals.

To clarify which biomarkers that discriminate SLE patients with and without neuropsychiatric involvement have been studied and which show consistent evidence of utility, we conducted this systematic review and, where possible, a meta-analysis of studies of analytes measured in serum, plasma, or CSF. We analyzed NPSLE overall and, when data allowed, by phenotype or manifestation, keep biofluids separate, standardize effect measures, and account for major methodological sources of heterogeneity.

## Methods

### Literature search

The literature search for published articles investigating analytes in circulation or CSF in the context of human NPSLE was conducted by an expert medical librarian (MA) using Ovid MEDLINE, Embase, and Cochrane CENTRAL, the latter to maximize sensitivity and capture reports indexed under drug headings but containing NPSLE biofluid outcomes. The search spanned from inception to March 13, 2024. The strategy included relevant subject headings and text-words for the concepts neuropsychiatric systemic lupus erythematosus (NPSLE) and analytes for each database. Additionally, the references from identified or related articles were manually reviewed to capture any missing relevant studies. The complete search strategies, including all subject headings and terms used, and the PRISMA 2020 checklist are available in **Supplementary Table 1**. This systematic review was not prospectively registered. The review methodology, including the eligibility criteria, data extraction, and planned analyses, was established before study selection and data synthesis.

### Inclusion criteria

Published studies that investigated soluble molecules (antibodies (Ab), cytokines, chemokines, inflammatory soluble factor mediators, enzymes, and metabolites) in blood and derivates (plasma and serum) or CSF in adult (≥16 years old) patients with NPSLE were included.

### Exclusion criteria

Studies were excluded if they were limited to imaging outcomes (no biofluid biomarkers), involved non-human subjects (animals or *in vitro*), focused on non-NPSLE manifestations, or included less than 20 NPSLE patients. Editorials and secondary research (narrative, scoping, and systematic reviews and meta-analyses) were excluded to avoid double-counting primary studies and mixing heterogeneous methods. Non-English articles were also excluded.

### Study selection

The Covidence platform was used to facilitate article selection. Three authors (AC, CM, and YN) independently screened the titles and abstracts of the first 100 studies captured in the literature search as a calibration exercise. This demonstrated high inter-reviewer agreement (97%; Fleiss’ *κ* = 0.94). Following the calibration, the remaining title and abstract records (*n =* 8,482) were allocated among the three reviewers for independent screening because of the large volume of retrieved records. Studies considered potentially eligible (*n =* 605) underwent full-text review. Two authors (CM and SD) independently assessed the first 100 full-text articles for eligibility according to the predefined inclusion and exclusion criteria. Agreement for these first 100 articles was 96% (Cohen’s *κ* = 0.834), with any discrepancies resolved by consensus. Following this calibration exercise, the remaining full-text articles were divided between the two reviewers for eligibility assessment. The PRISMA flow chart describing the process for studies selection is displayed in **Figure 1**.

**Figure 1:**
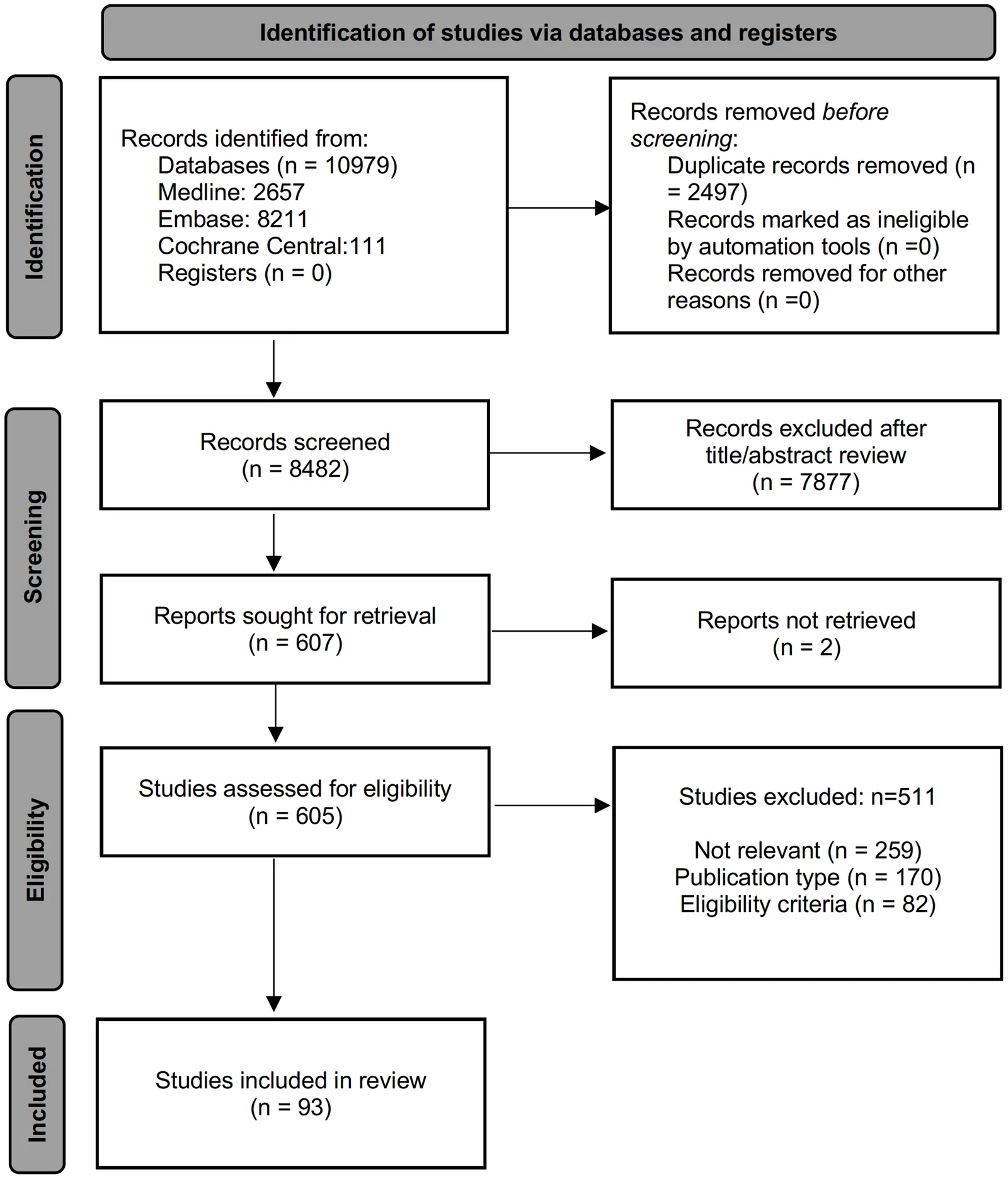
Flow chart describing the selection of studies. Not relevant (*n* = 259): non-NPSLE studies (*n* = 230), non-SLE or mixed-disease populations without separate SLE data (*n* = 14), and imaging-only studies without biofluid biomarkers (*n* = 15). Publication type (*n* = 170): review articles (*n* = 122), systematic reviews/meta-analyses without independent data (*n* = 7), conference abstracts (*n* = 20), and case reports (*n* = 21). Eligibility criteria (*n* = 82): case series with <20 NPSLE patients (*n* = 55), pediatric studies (*n* = 14), *in vitro* studies (*n* = 7), animal studies (*n* = 2), and non-English publications (*n* = 4).

### Outcome

The 1999 American College of Rheumatology (ACR) framework defines 19 NP syndromes, 12 of the central and seven of the peripheral nervous system (CNS and PNS, respectively) (8). CNS syndromes can be further categorized as diffuse psychiatric and neuropsychological or focal neurological (1). The latter is largely thought secondary to ischemia or venous thrombosis and, less often, vasculitis (1), whereas the pathogenic mechanisms leading to diffuse central NPSLE manifestations are likely multifactorial, including BBB injury/dysfunction, brain-reactive autoAb, soluble factors, cell-mediated inflammation, and many yet-unknown contributing pathways/factors (1, 9). Accordingly, to harmonize findings across NP phenotypes included in the retrieved studies, the reported NP manifestations were normalized to 1999 ACR syndromes and grouped as central (focal or diffuse) or peripheral. Non-ACR labels were mapped to the most appropriate ACR nomenclature.

### Data extraction

Two authors (CM and SD) independently extracted the relevant data pre-specified in a standardized form created in Covidence. CM cross-reviewed all data extracted to verify completeness and ensure suitability for analysis.

### Methodological quality appraisal

One author (CM) evaluated the methodological quality of non-randomized studies (case–control, cross-sectional, and cohort studies) using the Newcastle–Ottawa Scale (NOS) as previously described by our group (10) to identify potential biases related to study design (refer to **Supplementary Tables 2–4** for details).

### Statistical analysis

Random-effects meta-analyses were performed if at least five independent studies contributed data for a given analyte biofluid combination (serum/plasma or CSF); this was considered the minimum needed for stable estimates of heterogeneity. Importantly, data were insufficient to support meta-analysis stratified by specific NPSLE phenotypes/manifestations. For cross-sectional continuous outcomes, pooled effects were expressed as Hedges’ *g*, a standardized mean difference describing the difference in biomarker levels between groups (SLE patients with and without NPSLE) in standard deviation units. This approach was chosen because biomarker concentrations were measured using different assays, scales, and units across studies, precluding pooling of raw mean differences. Hedges’ *g* additionally incorporates a correction for small-sample bias, which was appropriate given the relatively small size of many included studies. Positive values indicate higher biomarker levels in NPSLE, and negative values indicate lower levels in NPSLE relative to the comparator group; for dichotomous outcomes, we pooled odds ratios. Where only graphical data were available, group means and standard deviations were approximated by digitizing plots using WebPlotDigitizer (version 5); these studies were treated as high risk of bias and included in the corresponding exclusion analyses. We planned to pool standardized mean change for longitudinal data, but too few studies reported compatible longitudinal measures to permit meta-analysis. We present pooled effect estimates with 95% confidence intervals (CIs), between-study heterogeneity using the *I*-squared statistic (*I*²), and 95% prediction intervals (PIs), and we assessed robustness with leave-one-out and high-risk of bias exclusion sensitivity analyses. For meta-analyses including ≥10 studies (anti–ribosomal P only), we explored small study effects by visual inspection of funnel plots; we did not perform formal tests of asymmetry given the modest number of studies.

## Results

A total of 8,482 articles were initially identified. After title and abstract screening, 605 articles were deemed eligible for full-text review, of which 93 were included in the final analysis (**Figure 1**). Among the included studies, 11 were cohort, three were case–control, and 79 were cross-sectional. The methodological appraisal is summarized in **Supplementary Files S2–S4**. Overall, the cohort and case–control studies were of good quality. Cross-sectional studies showed more variability: 18% (*n =* 14) were unsatisfactory, 54% (*n =* 43) were satisfactory, and 28% (*n =* 22) were good or above.

Most studies pooled multiple NPSLE phenotypes (central diffuse, central focal, and peripheral), whereas only a minority (*n =* 6) focused on a single manifestation (e.g., cognitive impairment (CI), psychosis). Where such distinctions were explicitly reported, we presented them separately in the review.

### Narrative synthesis for analytes with insufficient data for meta-analysis

#### Analytes in NPSLE compared to non-NPSLE patients

This section summarizes studies comparing the analyte levels between NPSLE and non-NPSLE patients where fewer than five independent studies contributed data precluding meta-analysis.

**Table 1** presents the list of analytes showing consistent differences, **Table 2** shows the list of analytes with no clear differences, and **Table 3** summarizes mixed or inconclusive findings. For each analyte, we report the matrix (serum/plasma or CSF) and whether subgroup analyses by NPSLE manifestation were performed. Summaries are presented per study given heterogeneity in assays, matrices, and designs.

**Table 1:** Analytes with less than five studies per biofluid predominantly showing differences between NPSLE and non-NPSLE.

Analyte	Matrix	Technique (units)	NPSLE (*n*)	Non-NPSLE (*n*)	Subgroup analysis^a^	Main findings	Author/year
α-Klotho	Serum CSF	ELISA (pg/mL)	34	25	No	Lower α-Klotho in CSF in NPSLE (*P* < 0.05)	Ushigusa/2016 (11)
Anti- Amyloid IgM and IgG	Serum	Protein microarray (MFI)	69	203	Inflammatory (38 of 69) and ischemic (31 of 69)^b^	Higher IgG and IgM NFI in NPSLE (effect size NR) No difference between subgroups	Van der Meulen/2017 (27)
Anti-AQP4 IgG	Serum	Live cell-based assay	11 Demyelinating NPSLE	97 non-demyelinating NPSLE	By default, with and without demyelination	Uncommon but enriched in demyelinating (3 of 11 vs. 0 of 97)	Mader/2018 (26)
Anti-α-internexin (INA) IgG	Serum CSF	ELISA (OD)	67 (CSF = 60)	85 (CSF = 28)	No	Higher in NPSLE (*p* < 0.001)	Lu/2010 (12)
Anti-collagen II (IgM and IgG)	Serum	Protein microarray (MFI)	69	203	Inflammatory (38 of 69) and ischemic (31 of 69)^b^	Higher IgM NFI in NPSLE (effect size NR) No difference between subgroups	Van der Meulen/2017 (27)
Anti-GP2 (glycoprotein 2) (IgG and IgM)	Serum	Protein microarray (MFI)	69	203	Inflammatory (38 of 69) and ischemic (31 of 69)^b^	Lower IgG NFI in NPSLE (effect size NR) No difference between subgroups	Van der Meulen/2017 (27)
Anti-heparin sulfate (IgG and IgM)	Serum	Protein microarray (MFI)	69	203	Inflammatory (38 of 69) and ischemic (31 of 69)^b^	Higher IgG and IgM NFI in NPSLE (effect size NR) No difference between subgroups	Van der Meulen/2017 (27)
Anti-MAA (malondialdehyde-acetaldehyde adducts) IgG	Serum	ELISA (aU/mL)	104	245	Inflammatory (51/104), ischemic (28/104), combined (25/104)^c^	More frequent in NPSLE (39% vs. 24%, *p =* 0.01) No difference between subgroups	Monahan/2022 (28)
**Anti-neuronal antibody** **(IgM and IgG)**	CSF serum	125I−protein A binding assay in human neuroblastoma cell line SK-N-SH, cultured (binding units)	27	18	No	Higher CSF IgG anti−neuronal positivity in NPSLE No difference in serum	Bluestein/1981 (13)
**Anti-neuronal antibody** **(IgG)**	CSF serum	Cell ELISA (paraformaldehyde-fixed SK-N-MC) (AU/mL)	34 psychosis (serum) 27 (CSF)	26 NPSLE non-psychosis (serum) 14 (CSF)	By default, psychosis vs. non-psychosis	Higher CSF anti−neuronal Ab in psychosis No difference in serum	Isshi-Hirohata/1998 (14)
**Anti-neuronal antibody** **(IgG)**	Serum	Mixed hemadsorption assay Neuroblastoma cell line (SK-N-SH) binding (+ binding activity ≥1:20) Glioblastoma cell line (LN229) (+ binding activity ≥1:20)	33	21	Diffuse (18), focal (15)^d^	Higher neuroblastoma-binding frequency in NPSLE (*p* < 0.038) Higher titers in diffuse vs. focal (neuroblastoma binding)	How/1985 (16)
Anti-nucleoporin 62 (IgG and IgM)	Serum	Protein microarray (Median NFI)	69	203	Inflammatory (38 of 69) and ischemic (31 of 69)^b^	Lower IgG and IgM NFI in NPSLE (effect size NR) No difference between subgroups	Van der Meulen/2017 (27)
Anti-prothrombin (IgG and IgM)	Serum	Protein microarray (MFI)	69	203	Inflammatory (38 of 69) and ischemic (31 of 69)^b^	Lower IgG NFI in NPSLE (effect size NR) No difference between subgroups	Van der Meulen/2017 (27)
**Anti-UCH-L1** **(UCH58-69, 119-130, and 203–214 epitopes)** **IgG**	Serum	ELISA (AU)	32	40	No	Higher anti-UCH58–69 in NPSLE (*p =* 0.0038)	Guo/2022 (17)
**Anti-UCH-L1** **(IgG)**	Serum CSF	ELISA(AU)	30	18	No	Higher in CSF in NPSLE (*p =* 0.042)	Li/2019 (15)
Anti-vimentin (IgM and IgG)	Serum	Protein microarray (MFI)	69	203	Inflammatory (38 of 69) and ischemic (31 of 69)^b^	Lower IgG and IgM NFI in NPSLE (effect size NR) No difference between subgroups	Van der Meulen/2017 (27)
APRIL	CSF serum	ELISA (pg/mL)	37	45	No	Higher in CSF in NPSLE (*p* < 0.001) No correlation with serum levels	George-Chandy/2008 (22)
**Basic fibroblast growth factor (FGF-2/bFGF)**	Serum	Milliplex MAP (pg/mL)	35	34	No	Higher in NPSLE (*p =* 0.035)	Hoang/2022 (29)
**Basic fibroblast growth factor (bFGF)**	CSF	Bio-Plex Human 27-plex (pg/mL)	34	25	No	Higher in NPSLE (*p* < 0.05)	Ushigusa/2016 (11)
GFAP (glial fibrillary acidic protein)	CSF	ELISA/ng/L	31	54	No	Higher in NPSLE (*p* < 0.05)	Trysberg/2003 (18)
GSH (reduced glutathione)	Serum	Biochemical assay	30 (CI)	20 (non-CI)	By default, CI vs. non-CI	Higher GSH associated with absence of CI (*p =* 0.03)	Cabral de Sousa/2023 (30)
IL-1 receptor antagonist (IL-1ra)	CSF	Bio-Plex Human 27-plex (pg/mL)	34	25	No	Lower in NPSLE (*p* < 0.05)	Ushigusa/2016 (11)
IL-5	CSF	Bio-Plex Human 27-plex (pg/mL)	34	25	No	Higher in NPSLE group (*p* < 0.05)	Ushigusa/2016 (11)
IL-7	CSF	Bio-Plex Human 27-plex (pg/mL)	34	25	No	Higher in NPSLE (*p* < 0.05)	Ushigusa/2016 (11)
**IL-9**	CSF	Bio-Plex Human 27-plex (pg/mL)	34	25	No	Higher in NPSLE (*p* < 0.05)	Ushigusa/2016 (11)
**IL-9**	Serum	Milliplex MAP (pg/mL)	35	34	No	Higher in NPSLE (*p =* 0.007)	Hoang/2022 (29)
IL-12 (p70)	CSF	Bio-Plex Human 27-plex (pg/mL)	34	25	No	Higher in NPSLE (*p* < 0.05)	Ushigusa/2016 (11)
IL-15	CSF	Bio-Plex Human 27-plex (pg/mL)	34	25	No	Higher in CSF in NPSLE (*p* < 0.05)	Ushigusa/2016 (11)
Lymphocytotoxic antibody	Serum	Micro cytotoxicity assay (+ or -)	50	48	CI (*n* = 27) No-CI (*n =* 71)	No difference between NPSLE and non-NPSLE, but more frequent positive in CI vs. non-CI (*p* < 0.02)	Long/1990 (31)
**MIP-1α (CCL3)**	Serum	Milliplex MAP (pg/mL)	35	34	No	Lower in NPSLE (*p* < 0.001)	Hoang/2022 (29)
**MIP-1α (CCL3)**	CSF	Bio-Plex Human 27-plex (pg/mL)	34	25	No	Higher in NPSLE (*p* < 0.0445)	Ushigusa/2016 (11)
**Neurofilament light (NfL)**	CSF	ELISA (ng/mL)	31	54	No	Intrathecal NfL markedly higher in NPSLE than SLE without CNS (*P* < 0.0001)	Trysberg/2003 (18)
**Neurofilament light (NfL)**	CSF	ELISA (pg/mL)	30 (CI)	37 (non-CI)	By default, CI vs. non-CI	Higher NfL concentrations were associated with impairment in psychomotor speed (OR 3.57 (1.48–8.64) *p* < 0.005) and motor function (OR 2.28 (1.09–4.76) p = 0.03)	Tjensvoll/2021 (19)
**Neurofilament light (NfL)**	Plasma	Quanterix Simoa SR-X (NR)	47 (CI)	54 (non-CI)	By default, CI vs. non-CI	Mean NfL higher in CI (*p =* 0.03). Frequency of positive no different (*p =* 0.27)	Hanly/2022 (20)
**Neurofilament light (NfL)**	Serum	ELISA (NR)	63	27	No	No difference	Ahmadzade/2024 (21)
PAI-1 (plasminogen activator inhibitor-1)	CSF	ELISA (ng/mL)	33	56	No	Higher in NPSLE (*P* < 0.05)	Kwiecinski/2009 (25)
P-selectin	CSF	Fluorescent bead-based assay/Luminex (pg/mL)	54	20	No	Higher in NPSLE (*p* < 0.01)	Lu/2010 (24)
**S100A8/A9** **(calprotectin)**	Serum	ELISA (ng/mL)	116 (CI)	93 (non-CI) 81 (Indet-CI)	By default, CI vs. non-CI and Indet-CI	Higher in CI vs. non-CI (*p =* 0.006) S100A8/A9 had a negative relationship with multiple CI tests	Muñoz-Grajales/2024 (32)
**S100A8/A9** **(calprotectin)**	Serum CSF	ELISA (ng/mL)	44 (21 had CSF)	28 (12 had CSF)	No	Serum S100A8/A9 higher in SLICC A and ACR-defined NPSLE No difference in CSF levels	Zervides/2022 (33)
TGF-β	CSF	ELISA (pg/mL)	36	71	No	Higher in NPSLE (*p* < 0.01)	Trysberg/2004 (23)
Vascular cell adhesion molecule 1 (VCAM-1)	CSF	Fluorescent bead-based assay/Luminex (pg/mL)	54	20	No	Higher in NPSLE (*p* < 0.01)	Lu/2010 (24)

Analytes in bold were reported in ≥2 independent studies.

aU/mL, arbitrary units per milliliter; AU, arbitrary units; ACS, acute confusional state; CI, cognitive impairment/dysfunction; CVD, cerebrovascular disease; HC, healthy control; Indet, indeterminate; MFI, median fluorescence intensity; NR, not reported; pNA, paranitroaniline; TLC, thin-layer chromatography.

^a^ Subgroup analyses (as designed per study) indicated when reported.

^b^ Van der Meulen’s paper classification: inflammatory (clinical suspicion of an autoimmune-mediated cause and a normal MRI); ischemic (infarctions or thrombotic lesions of brain arteries or venous sinus thrombosis on MRI). Based on expert opinions of the multidisciplinary consensus group (27).

^c^ Monahan’s paper classification: inflammatory NPSLE (started immunosuppressive treatment or had a dosage increase), ischemic NPSLE (began anticoagulant therapy), and combined (patients received a combination of both) (6).

^d^ How’s paper classification: opinion of a neurologist (focal: attributed to lesions of specific areas within the nervous system; diffuse: could not be attributed to a specific, localizable lesion) (16).

**Table 2:** Analytes with less than five studies by biofluid showing no differences between NPSLE and non-NPSLE (subgroup analyses indicated when reported).

Analyte	Matrix	Technique (units)	NPSLE (*n*)	Non-NPSLE (*n*)	Subgroup analysis^a^	Main findings	Author/year
Anti-AGE (advanced glycation end-product) (IgG)	Serum	ELISA (aU/mL)	104	245	Inflammatory (51/104), ischemic (28/104), combined^b^ (25/104)	No difference between NPSLE and non-NPSLE or subgroups	Monahan/2022 (28)
Anti-GAPDH (glyceraldehyde-3-phosphate dehydrogenase) (IgG)	Serum	ELISA (aU)	35	95	No by NPSLE syndromes	NPSLE aU higher in non-NPSLE, but marginal difference (*p =* 0.058)	Sun/2019 (42)
Anti-GFAP (glial fibrillary acidic protein) (IgG)	Serum	MagPix System (Luminex) (MFI)	47 (CI)	54 (non-CI)	By default, CI vs. non-CI	No difference (*p =* 0.07)	Hanly/2022 (20)
Anti-GRP78 (glucose-regulated protein 78) (IgG)	Serum	ELISA (U/mL)	88	20	ACS (*n =* 28), non-ACS diffuse (*n =* 30), focal (*n =* 30)	No difference between NPSLE and non-NPSLE Elevated in ACS vs. other diffuse (p= 0.0001) and focal (*p =* 0.0002)	Matsueda/2018 (41)
Anti-MAG (myelin-associated glycoprotein) (IgG)	Serum	ELISA (ng/mL)	119	135	Seizure (*n =* 45), psychosis (*n =* 17), mood disorder (*n =* 21), stroke (*n =* 14), mononeuropathy (*n =* 13), headache (*n =* 12), demyelinating (*n =* 7)	No difference between NPSLE and non-NPSLE or subgroups	Seth/2020 (39)
**Anti-MOG (myelin oligodendrocyte glycoprotein)** **(IgG)**	Serum	Live cell-based assay	11 demyelinating NPSLE	97 non-demyelinating NPSLE	By default, with and without demyelinating	Detected infrequently (*n =* 3)	Mader/2018 (26)
**Anti-MOG (myelin oligodendrocyte glycoprotein)** **(IgG)**	Serum	ELISA (ng/mL)	119	135	Seizure (*n =* 45), psychosis (*n =* 17), mood disorder (*n =* 21), stroke (*n =* 14), mononeuropathy (*n =* 13), headache (*n =* 12), demyelinating (*n =* 7)	No difference in NPSLE vs. non-NPSLE Significantly elevated in mood disorder (*p =* 0.003)	Seth/2020 (39)
Anti-MPP1 (M-phase phosphoprotein 1- KIF20B) (IgG)	Serum	MagPix System (Luminex) (MFI)	47 (CI)	54 (non-CI)	By default, CI vs. non-CI	No difference	Hanly/2022 (20)
Anti-Myelin antibody (AMA) (IgA)	Serum	ELISA (ng/mL)	119	135	Seizure (*n =* 45), psychosis (*n =* 17), mood disorder (*n =* 21), stroke (*n =* 14), mononeuropathy (*n =* 13), headache (*n =* 12), demyelinating (*n =* 7)	No difference in NPSLE vs. non-NPSLE or by subgroups	Seth/2020 (39)
Anti- neurofilament antibody (ANFA) (IgG)	Serum	Western blot (band intensity)	22	34	Diffuse CSN (*n =* 14), focal CNS (*n =* 10)	No significant difference between NPSLE and non-NPSLE (*p =* 0.13) Elevated in diffuse CNS vs. non-NPSLE (*p* < 0.015)	Robbins/1988 (43)
Anti-phosphatidylinositol (PI), phosphatidic acid (PA), phosphatidylserine (PS) (IgG and IgM)	Serum	ELISA (aU/mL)	44	17	No	No difference	Afeltra/2003 (40)
Anti-SBSN (suprabasin) antibodies (IgG)	Serum	Luciferase immunoprecipitation system (LIPS) (relative concentration)	35	34	No	Tend to be higher in NPSLE but no significant difference from non-NPSLE (*p =* 0.2)	Hoang/2022 (29)
APP (amyloid precursor protein)	CSF	ELISA (pg/mL)	26	60	No	No difference between NPSLE and non-NPSLE	Trysberg/2004 (23)
BDNF (brain-derived neurotrophic factor)	Serum	ELISA (pg/mL)	35	12	No	No difference	Noris-Garcia/2018 (51)
β-Amyloid (Aβ42)	CSF	ELISA (pg/mL)	36	71	No	No difference between NPSLE and non-NPSLE	Trysberg/2004 (23)
Chemokine (C-X-C motif) ligand 1 (CXCL1/GRO)	Serum	Milliplex MAP (pg/mL)	35	34	No	No difference in levels between NPSLE and non-NPSLE	Hoang/2022 (29)
D-dimer, Urokinase and tissue plasminogen activator (uPA and tPA)	CSF	ELISA (ng/mL)	33	56	No	D-dimer and uPA no difference; tPA not detectable	Kwiecinski/2009 (25)
Eotaxin (CCL11)	CSF	Bio-Plex Human 27-plex (pg/mL)	34	25	No	No difference	Ushigusa/2016 (11)
**IFN-α**	CSF Serum	Luminex/Milliplex (pg/mL)	34	44 16 different SLE patients for CSF (surgical)	Seizure (*n* = 13), severe refractory headache (*n* = 7), ACS (*n =* 8), CVD (*n =* 4), psychosis (*n =* 1), and transverse myelitis (*n =* 1)	No difference in serum IFN-α levels among patients with NPSLE, SLE controls, and non-autoimmune groups Higher in CSF in ACS vs. other NPSLE syndromes	Fragoso-Loyo/2012 (35)
**IFN-α**	CSF Serum	DELFIA (NR)	28	15 (14 CSF)	No	No difference	Jönsen/2003 (36)
**IFN-γ**	CSF	Cytometric bead array (pg/mL)	42	16	No	No difference	Fragoso-Loyo/2007 (37)
**IFN-γ**	Serum	ELISA (ng/mL)	116 (CI)	93 (non-CI) 81 (Indet-CI)	By default, CI vs. non-CI and Indet-CI	No difference No correlation with impaired domains	Muñoz-Grajales/2024 (32)
IL-1β	CSF	Bio-Plex Human 27-plex (pg/mL)	34	25	No	No difference	Ushigusa/2016 (11)
**IL-4**	CSF	Cytometric bead array (pg/mL)	42	16	No	No difference	Fragoso-Loyo/2007 (37)
**IL-4**	CSF	Bio-Plex Human 27-plex (pg/mL)	34	25	No	No difference	Ushigusa/2016 (11)
IL-8	Serum	Milliplex MAP (pg/mL)	35	34	No	No difference	Hoang/2022 (29)
**IL-10**	CSF	Cytometric bead array (pg/mL)	42	16	No	No difference	Fragoso-Loyo/2007 (37)
**IL-10**	Serum	Milliplex MAP (pg/mL)	35	34	No	No difference in levels between NPSLE and non-NPSLE	Hoang/2022 (29)
**IL-10**	CSF Serum	DELFIA (NR)	28	15 (14 CSF)	No	No difference	Jönsen/2003 (36)
**IL-10**	Serum	ELISA (ng/mL)	116 (CI)	93 (non-CI) 81 (Indet-CI)	By default, CI vs. non-CI and Indet-CI	No difference No correlation with impaired domains	Muñoz-Grajales/2024 (32)
**IL-10**	CSF	Bio-Plex Human 27-plex (pg/mL)	34	25	No	Higher in NPSLE *p =* 0.013); however, 27 analytes were measured and no mention of correction for multiple comparisons	Ushigusa/2016 (11)
IL-13	CSF	Bio-Plex Human 27-plex (pg/mL)	34	25	No	No difference	Ushigusa/2016 (11)
MDC (CCL22)	Serum	Milliplex MAP (pg/mL)	35	34	No	No difference in levels between NPSLE and non-NPSLE	Hoang/2022 (29)
MIG (CXCL9)	CSF	Cytometric bead array (pg/mL)	42	16	No	No difference	Fragoso-Loyo/2007 (37)
MIP-1β (CCL4)	CSF	Bio-Plex Human 27-plex (pg/mL)	34	25	No	No difference	Ushigusa/2016 (11)
MMP-2	CSF	Activity assay/enzymatically active forms (pg/mL)	43	71	No	No difference	Trysberg/2004 (34)
Myeloperoxidase (MPO)	Serum	Biochemical assay	30 (CI)	20 (non-CI)	By default, CI vs. non-CI	No difference overall	Cabral de Sousa/2023 (30)
NGAL (Lipocalin-2)	Serum	ELISA (ng/mL)	116 (CI)	93 (non-CI) 81 (Indet-CI)	By default, CI vs. non-CI and Indet-CI	No difference	Muñoz-Grajales/2024 (32)
PDGF-BB (platelet-derived growth factor-BB)	CSF	Bio-Plex Human 27-plex (pg/mL)	34	25	No	No difference	Ushigusa/2016 (11)
Soluble CD 40 receptor ligand (sCD40L)	Serum	Milliplex MAP (pg/mL)	35	34	No	No difference in levels between NPSLE and non-NPSLE	Hoang/2022 (29)
Tau	CSF	ELISA (pg/mL)	36	71	No	No difference between NPSLE and non-NPSLE	Trysberg/2004 (23)
**TWEAK**	Serum	ELISA (pg/mL)	28	53	Headache (*n =* 18), seizures (*n =* 12), CVD (*n =* 6), CI (*n =* 15), and anxiety (*n =* 14)	No difference between NPSLE vs. non-NPSLE or subgroups	Balajková/2020 (38)
**TWEAK**	Serum	ELISA (ng/mL)	116 (CI)	93 (non-CI) 81 (Indet-CI)	By default, CI vs. non-CI and Indet-CI	No difference No correlation with impaired domains	Muñoz-Grajales/2024 (32)
**TWEAK**	CSF	ELISA (ng/mL)	30 (CI)	37 (non-CI)	By default, CI vs. non-CI	No difference No correlation with impaired domains	Tjensvoll/2021 (19)
**Vascular endothelial growth factor (VEGF)**	Serum	Milliplex MAP (pg/mL)	35	34	No	Higher than in the non-NPSLE group but no difference with the HC group (pairwise *p =* 0.015 and *p =* 0.15, respectively)	Hoang/2022 (29)
**Vascular endothelial growth factor (VEGF)**	CSF	Bio-Plex Human 27-plex (pg/mL)	34	25	No	No difference	Ushigusa/2016 (11)

Analytes in bold were reported in ≥2 independent studies.

^a^ Subgroups refer to central, focal, or individual NPSLE syndromes.

aU/mL, arbitrary units per milliliter; AU, arbitrary units; ACS, acute confusional state; MFI, median fluorescence intensity; NR, not reported; TLC, thin-layer chromatography; CI, cognitive impairment or dysfunction; CVD, cerebrovascular disease; HC, healthy control; Indet, indeterminate; pNA, paranitroaniline.

^b^ Monahan’s paper classification: inflammatory NPSLE (started immunosuppressive treatment or had a dosage increase), ischemic NPSLE (began anticoagulant therapy), and combined (patients who received a combination of both) (6).

**Table 3:** Analytes with less than five studies per biofluid showing mixed/inconclusive findings between NPSLE and non-NPSLE; subgroup analyses indicated when reported.

Analyte	Matrix	Technique (units)	NPSLE (n)	Non-NPSLE (n)	Subgroup analysis^a^	Main findings	Author/year
Anti-ganglioside antibodies (AGA) (IgG and IgM)	Serum CSF	ELISA (OD) Antigen (solution of gangliosides from bovine brain)	40	10	No	IgG AGA elevated in CSF in NPSLE (*p* < 0.0002) IgM AGA in serum elevated in NPSLE, *p =* 0.006)	Pereira/1992 (44)
Anti-ganglioside antibodies (AGA) GM1, GM2, GM3, asialo-GM1 GD1a, GD1b, GD3, GT1b, GQ1b) IgG and IgM	Serum	ELISA(OD) confirmed by TLC Antigen (GM1, GM2, GM3, asialo- GM1 (aGM1), GD1a, GD1b, GD3, GT1b and GQ1b)	65	50	Group 1 (diffuse CNS = 29), group 2 (focal CNS = 48), Group 3 (PNS = 11)^b^	No correlation between AGA and neuropsychiatric manifestations	Labrador-Horrillo/2012 (45)
B-cell activating factor (BAFF)	CSF Serum	ELISA (pg/mL)	37	45	No	No difference	George-Chandy/2008 (22)
B-cell activating factor (BAFF)	Serum	ELISA (pg/mL)	105	101	No	Elevated in NPSLE vs. non-NPSLE (*p* < 0.001)	Pang/2024 (46)
Granulocyte- macrophage CSF (GM-CSF)	CSF	Bio-Plex Human 27-plex (pg/mL)	34	25	No	Higher in CSF in NPSLE (effect size NR)	Ushigusa/2016 (11)
Granulocyte- macrophage CSF (GM-CSF)	CSF Serum	Bio-Plex (pg/mL)	30	22	No	No difference	Yoshio/2016 (47)
Granulocyte colony-stimulating factor (G-CSF)	CSF Serum	Bio-Plex (pg/mL)	30	22	No	Elevated in CSF in NPSLE (*p =* 0.0001)	Yoshio/2016 (47)
Granulocyte colony-stimulating factor (G-CSF)	CSF	Bio-Plex Human 27-plex (pg/mL)	34	25	No	No difference	Ushigusa/2016 (11)
High‐mobility group box protein 1 (HMGB1)	Serum	ELISA (NR)	63	27	No	No difference	Ahmadzade/2024 (21)
High‐mobility group box protein 1 (HMGB1)	CSF Serum	ELISA (ng/mL)	86 (59 CSF)	102 (No CSF)	Seizures (*n =* 21) Non-seizures (*n =* 65)	Higher serum levels in NPSLE (*p* < 0.001) No difference by subgroup	Huang/2020 (48)
IL-2	CSF	Cytometric bead array (pg/mL)	42	16	No	No difference	Fragoso-Loyo/2007 (37)
IL-2	CSF	Bio-Plex Human 27-plex (pg/mL)	34	25	No	Higher in NPSLE (*p =* 0.0007)	Ushigusa/2016 (11)
IL-17	CSF	Fluorescent bead-based assay/Luminex (pg/mL)	54	20	No	No difference	Lu/2010 (24)
IL-17	CSF	Bio-Plex Human 27-plex (pg/mL)	34	25	No	Higher in CSF in NPSLE (*p* < 0.05)	Ushigusa/2016 (11)
IP-10	CSF	Cytometric bead array (pg/mL)	42	16	No	No difference	Fragoso-Loyo/2007 (37)
IP-10	CSF	Fluorescent bead-based assay/Luminex (pg/mL)	54	20	No	Reported lower levels in NPSLE vs. non-NPSLE (*p* < 0.01)	Lu/2010 (24)
IP-10	CSF	Bio-Plex Human 27-plex (pg/mL)	34	25	No	No difference	Ushigusa/2016 (11)
IP-10	CSF Serum	Bio-Plex (pg/mL)	30	22	No	Elevated in CSF in NPSLE (*p =* 0.0028)	Yoshio/2016 (47)
MMP-9	Serum	ELISA (NR)	63	27	No	No difference	Ahmadzade/2024 (21)
MMP-9	Serum	ELISA (ng/mL)	116 (CI)	93 (non-CI) 81 (Indet-CI)	By default, CI vs. non-CI and Indet-CI	Elevated in CI vs. non-CI	Muñoz-Grajales/2024 (32)
MMP-9	CSF	Activity assay/enzymatically active forms (pg/mL)	43	71	No	Intrathecal MMP-9 significantly higher in NPSLE than SLE without CNS involvement	Trysberg/2004 (34)
RANTES (CCL5)	CSF	Cytometric bead array (pg/mL)	42	16	No	Elevated in NPSLE CSF (*p =* 0.0006)	Fragoso-Loyo/2007 (37)
RANTES (CCL5)	CSF	Bio-Plex Human 27-plex (pg/mL)	34	25	No	No difference	Ushigusa/2016 (11)
S100B	Serum	ELISA (pg/mL)	116 (CI)	93 (non-CI) 81 (Indet-CI)	By default, CI vs. non-CI and Indet-CI	Tendency, yet no significant difference, higher in CI vs. non-CI (*p =* 0.055)	Muñoz-Grajales/2024 (32)
S100B	Serum	ELISA (ng/mL)	35	12	PNP (*n =* 27), non- PNP (*n =* 20)	ROC to discriminate PNP vs. non-PNP with a cutoff 0.0218: AUC 0.742; Se 81% and Sp 67%	Noris-Garcia/2018 (51)
S100B	Serum	Luminescence immunoassay (ng/mL)	23	64	Acute NPSLE (*n =* 18) Chronic/stable >3 months NPSLE (*n =* 5)	Higher S100B levels in NPSLE (*p* < 0.01) No difference by subgroups S100B levels showed good discriminatory capacity for NPSLE (cutoff >0.125 ng/mL = Se 73.9%, Sp 79.8%, AUC)	Schenatto/2006 (49)
S100B	CSF	ELISA (pg/mL)	30 (CI)	37 (non-CI)	By default, CI vs. non-CI	No difference	Tjensvoll/2021 (19)
S100B	Serum CSF	ELISA (μg/L)	65	92	OBS (*n =* 6), seizures (*n =* 11), CVD (*n =* 9), psychosis (*n =* 15), Headache (*n =* 13), neuropathy (type NR; *n =* 11)	Higher serum levels in NPSLE (*p* < 0.001) No difference by subgroups	Xu-yan/2008 (50)
TNF-α	CSF	Cytometric bead array (pg/mL)	42	16	No	No difference	Fragoso-Loyo/2007 (37)
TNF-α	CSF	Bio-Plex Human 27-plex (pg/mL)	34	25	No	Higher in NPSLE (*p =* 0.0254)	Ushigusa/2016 (11)
TNF-α	Serum	Milliplex MAP (pg/mL)	35	34	No	No difference	Hoang/2022 (29)
TNF-α	Serum	ELISA (ng/mL)	116 (CI)	93 (non-CI) 81 (Indet-CI)	By default, CI vs. non-CI and Indet-CI	No difference; no correlation with impaired domains	Muñoz-Grajales/2024 (32)

aU/mL, arbitrary units per milliliter; AU, arbitrary units; ACS, acute confusional state; MFI, median fluorescence intensity; NR, not reported; TLC, thin-layer chromatography; CI, cognitive impairment or dysfunction; CVD, cerebrovascular disease; HC, healthy control; Indet, indeterminate; pNA, paranitroaniline; PNP, polyneuropathy; Se, sensitivity; Sp, specificity; AUC, area under the curve; OBS, organic brain syndrome; CVD, cerebral vascular accident.

^a^ Subgroups refer to central, focal, or individual NPSLE syndromes.

^b^ Labrador–Horrillo’s paper classification: group 1 (diffuse psychiatric or neuropsychological syndromes such as anxiety disorder, cognitive disorder, acute confusional state, mood disorder, and psychosis), group 2 (neurological syndromes of the central nervous system, such as cerebrovascular disease (strokes), aseptic meningitis, seizures, and myelopathy), and group 3 (neurological syndromes of the peripheral nervous system, such as acute inflammatory demyelinating polyradiculoneuropathy, mononeuropathy, and polyneuropathy) (45).

As summarized in **Table 1**, with few exceptions, most analytes that showed significant differences between NPSLE and non-NPSLE were reported in a single study. Therefore, future studies are required to validate the findings.

Overall, in CSF, α-Klotho was reduced (11), whereas anti-α-internexin (12), anti-neuronal (in two independent studies) (13) (14), and anti-UCH-L1 Ab were all increased (15); anti-neuronal Ab were particularly increased in psychosis in one study (14). In both paired CSF–serum studies, anti-neuronal Ab were defined by reactivity to the SK-N-SH neuroblastoma cell line, and group differences were seen only in CSF, with no corresponding differences in serum (13) (14). By contrast, a single serum-only study from 1985 using a mixed hemadsorption assay reported more frequent neuroblastoma-binding anti-neuronal IgG in NPSLE than in non-NPSLE, with higher titers in diffuse than focal CNS disease (16). A similar pattern was seen for anti-UCH-L1: one serum-only study found higher anti-UCH58–69 IgG in NPSLE (17), whereas the paired CSF–serum study found elevated levels restricted to the CSF (15).

Markers of glial and axonal injury were clearly abnormal in CSF. GFAP was about threefold higher in NPSLE (18), and one study reported a markedly increased CSF NfL in NPSLE compared with SLE without CNS involvement (18). An independent study found that higher CSF NfL was associated with poorer psychomotor speed and motor function (19). None of these studies included paired serum measurements, and the two independent studies that examined circulating NfL suggested no clear difference in blood: Hanly et al. (20) reported higher mean plasma NfL in patients with CI, but not a higher frequency of abnormal values, and Ahmadzade et al. (21) found no difference in serum NfL between groups.

Multiple soluble mediators were also higher in CSF, including APRIL (without correlation to serum) (22), IL-12 (p70) (18), TGF-β (23), and from the Bio-plex Multiplex panel reported by Ushigusa et al. (11), basic FGF IL-1ra, IL-5, IL-7, IL-9, IL-15, and MIP-1α/CCL3. Similarly, the vascular/hemostatic markers P-selectin (24), PAI-1 (25), and VCAM-1 (24) were found to be increased in CSF from NPSLE patients.

Studies that examined serum or plasma levels alone reported a more variable pattern of findings. In the group of serum autoAbs, anti-AQP4 Abs were rare overall but enriched in demyelinating NPSLE (26). Anti-amyloid IgG/IgM (27), anti-MAA IgG (28), and anti-collagen II IgM (27) were higher in NPSLE, whereas anti-vimentin (27), anti-GP2 (27), anti-nucleoporin 62 (27), and anti-prothrombin (27) (IgG and IgM) were lower. These results came largely from a single protein-microarray study using median fluorescence intensity (MFI) as the readout (27). In addition, basic FGF, IL-9, and MIP-1α were not only increased in CSF but were also reported as elevated in serum in NPSLE versus non-NPSLE in a separate multiplex (Milliplex MAP) study (29).

Several serum markers were evaluated specifically in relation to CI. A higher serum reduced glutathione (GSH) level was associated with the absence of CI (30). Lymphocytotoxic Ab did not differ between NPSLE and non-NPSLE overall, but positivity was more frequent in patients with CI (31). Serum S100A8/A9 (calprotectin) was increased in cognitively impaired compared with cognitively intact SLE patients (32), and in an independent cohort it was also higher in SLICC A and ACR-defined NPSLE than in non-NPSLE, whereas CSF S100A8/A9 showed no group difference (33).

Taken together, these findings support a multifactorial pathophysiology in NPSLE involving immune-mediated neuronal targeting, glial and axonal damage, endothelial and fibrinolytic disturbances, and systemic inflammatory–oxidative imbalance. They also showed that serum and plasma measurements did not consistently mirror CSF abnormalities for several analytes (e.g., APRIL, S100A8/A9, UCH-L1, and NfL).

**Table 2** summarizes the analytes that, although biologically plausible, did not differ between NPSLE and non-NPSLE patients. Classical CSF neurodegeneration markers (Aβ42, tau, and APP) (23) and the matrix enzyme MMP-2 (34) all showed similar levels in both groups. Most cytokines, chemokines, and growth factors measured in CSF and/or serum—including IFN-α (35, 36), IFN-γ (32, 37), IL-1β (11), IL-4 (11, 37), IL-8 (29), IL-13 (11), eotaxin/CCL11 (11), MIG/CXCL9 (37), MIP-1β/CCL4 (11), MDC/CCL22 (29), PDGF-BB (11), TWEAK (19, 32, 38), and VEGF (11, 29), also showed no consistent group differences.

IL-10 was evaluated in five studies (11, 29, 32, 36, 37), but there were fewer than five observations per biofluid, precluding a separate meta-analysis by matrix. Significantly higher CSF IL-10 levels in NPSLE were observed in a single study (11), but this finding emerged from multiple parallel testing with unclear adjustment for multiple comparisons, making a type I error likely. Similarly, one study found higher serum VEGF levels in NPSLE versus SLE patients, but the levels did not differ from those seen in HC (29) and this result was not replicated in another independent study (11).

Regarding serum autoAb, anti-AGE (28), anti-MAG (39), anti-MOG (26, 39), anti-myelin Ab (39), non-criteria antiphospholipid Ab (anti-PI, PA, and PS) (40), anti-GFAP (20), anti-MPP1 (20), anti-SBSN (29), anti-GRP78 (41), anti-GAPDH (42), and ANFA Ab (43) either did not differ between NPSLE and non-NPSLE or showed only borderline trends [e.g., marginally higher anti-GAPDH (42) and higher anti-SBSN without statistical significance (29)]. Similarly, coagulation/fibrinolytic markers (D-dimer, uPA/tPA) (25), MPO (30), and NGAL (32) in serum showed no differences.

Importantly, three markers that did not differ significantly between NPSLE and non-NPSLE groups were associated with specific NPSLE syndromes—higher serum anti-GRP78 in acute confusional state (ACS) (41), higher serum ANFA in diffuse CNS disease (43), and higher serum anti-MOG in mood disorder (39)—warranting further investigation in their respective contexts. These results also highlight the utility of testing specific biomarkers in individual NPSLE phenotypes rather than grouping all NPSLE together. Given the vast differences in clinical NPSLE phenotypes, even if certain analytes are found to be elevated, it is not possible to know which individual syndromes are associated. Conversely, when no differences are found between NPSLE and non-NPSLE for a given analyte, a relevant association may be missed due to diluting effects from grouping all phenotypes together as illustrated by these three analytes.

**Table 3** summarizes markers for which study findings were conflicting or inconsistent, yielding no clear consensus. For these analytes, the number of studies per biofluid fell below our minimum threshold of five for meta-analysis. As described below, seven analytes were investigated in only two studies (anti-ganglioside Ab (AGA), BAFF, GM-CSF, G-CSF, HMGB1, IL-12, and RANTES), MMP-9 in three studies (two serum and one CSF), IP-10 in four CSF studies (one of which also reported serum levels), TNF-α in four studies (two serum and two CSF), and S100B in five studies, but with fewer than five in any single matrix.

AGA illustrates a pattern of conflicting results: one study reported elevated CSF IgG AGA and serum IgM AGA in NPSLE (44), whereas another found no clear correlation of serum levels with specific neuropsychiatric manifestations (45). A similar picture emerges for BAFF (22, 46), GM-CSF (11, 47), G-CSF (11, 47), IP-10 (11, 24, 37, 47), IL-2 (11, 37), IL-17 (11, 37), RANTES (11, 37), TNF-α (11, 29, 32, 37), and HMGB1 (21, 48), where some studies reported higher levels in NPSLE, while others showed no difference. Methodological heterogeneity, including the use of different assays or sampling CSF versus serum, small sample sizes, and varying definitions of NPSLE likely contributed to these discrepancies.

The markers of matrix turnover and astroglia injury presented in **Table 3** also show heterogeneous results. Intrathecal MMP-9 was higher in NPSLE compared with non-NPSLE in one study (34), and serum MMP-9 was associated with CI in another (32), yet a separate serum study found no group difference (NPSLE vs. non-NPSLE) (21).

S100B was among the most frequently evaluated analytes with discrepant results; however, serum studies suggest higher levels in NPSLE compared to non-NPSLE. Two serum studies reported higher S100B concentrations in NPSLE (49, 50), with one also demonstrating reasonable discriminatory performance between the two groups (49). A separate study focused on peripheral neuropathy (PNP) subgroups also showed acceptable discrimination between NPSLE and non-NPSLE (51) but did not compare PNP with non-PNP or non-NPSLE patients (51). Our group observed a trend toward higher serum S100B in CI that did not reach statistical significance (32). In contrast, the CSF S100B levels were not informative (19, 50).

#### Analytes in NPSLE compared with non-SLE patients

This section summarizes studies that compared analyte levels in NPSLE with non-SLE comparator groups, including healthy controls (HC) and patients with other neurological or rheumatic disorders (e.g., meningitis/encephalitis, multiple sclerosis (MS), neuromyelitis optical (NMO), and other rheumatic diseases). For every analyte–matrix combination (CSF or serum/plasma), fewer than five independent studies were available, which was insufficient for a quantitative meta-analysis.

The findings are presented at the study level and consolidated in a single table (**Table 4**). For each study, the table shows a list of analyte(s), matrix, comparator group(s), main results, and whether NPSLE phenotype-specific subgroup analyses were performed. Two studies that included multiplex cytokine and non-cytokines analytes are shown as separate entries (29, 52) to facilitate readability. A study-level summary was chosen because assays, matrices, comparator groups, and designs varied substantially across studies, limiting description by analyte as done in the previous section.

**Table 4:** Studies evaluating differences in CSF and serum analyte levels between NPSLE and non-SLE groups.

Studies	Analyte(s)	Matrix	Technique (units)	NPSLE (*n*)	Non-SLE (comparator groups, *n*)	Subgroup analysis^a^	Main findings
Baraczka/2004 (53)	TNF-α, IL-6, IL-10	CSF Serum	ELISA (pg/mL)	50	MS (*n =* 50) HC (*n =* 50; 25 with degenerative disease of spinal column and 25 with ischemic cerebrovascular lesions)	Demyelinating lesions (*n =* 20) Multifocal ischemic lesions (*n =* 30)	CSF TNF-α higher in NPSLE vs. HC (*P* < 0.01) IL-6 and IL-10 CSF and serum were higher in NPSLE vs. HC (*P* < 0.01) TNF-α CSF higher in MS vs. NPSLE (*P* < 0.01) CSF IL-10 higher in NPSLE vs. MS (*P* < 0.01) No differences between NPSLE subgroups
Dellalibera-Joviliano/2003 (52)	Pre-kallikrein, kininase II activity, kallikrein, high-MW (HKg) and low-MW (LKg) kininogen	Plasma CSF	Fluorometric assay (U/mL for pre-kallikrein; μmol His-Leu/mL plasma or nmoL His-Leu/mL CSF for kininase II; μmol pNA/mL for kallikrein) ELISA for HKg and LKg (µg bradykinin equivalents/mL)	29	HC (*n =* 29) No comparison with non-NPSLE	No	All were significantly higher in plasma and CSF in NPSLE vs. HC
Dellalibera-Joviliano/2003 (52)	IL-1ß, IL-6, IL-8, IL-10, TNF-α	Plasma CSF	ELISA (pg/mL)	29	HC (*n =* 29) No comparison with non-NPSLE	No	IL-6, IL-10 and TNF-α elevated in plasma and CSF in NPSLE vs. HC (*p* < 0.0001) and IL-1ß elevated in plasma IL-8 and IL-1ß not detected in NPSLE and HC CSF IL-8 showed no difference in NPSLE plasma vs. HC
Fragoso-Loyo/2007 (37)	IL-2, IL-4, IL-6, IL-8, IL-10, IFN-γ, IP-10, MIG, MCP-1, RANTES, TNF-α	CSF	Cytometric bead array (pg/mL)	42	31 HC-elective surgery went to spinal block (*n =* 25) SLE and infectious meningitis (*n =* 6)	No	CSF IL-6, IL-8, MIG, IP-10, MCP-1, RANTES all higher in NPSLE vs. HC All higher in SLE with meningitis vs. NPSLE except TNF-α IL-2, IL-4, IL-10, TNF-α, and IFN-**γ** were either low or undetectable in most patients
Hirohata-Tanaka/2019 (59)	Anti- N-methyl- D- aspartate receptor subunits 1 and 2 (NR1/NR2) (IgG)	Serum	Cell-based assay (HEK293 NR1/NR2B; anti-human IgG detection)/(titer ≥1:40)	31	HC (*n =* 18)	No	anti-NR1/NR2 was positive only in 2 of 31 patients NR1/ NR2 did not correlate with anti-NR2
Hoang/2022 (29)	CXCL1, EGF, eotaxin, FGF-2/bFGF, FLT-3 L, fractalkine, G-CSF, GM-CSF, IFN-α2, IFN-γ, IL-1α, IL-1β, IL-1Ra, IL-2, IL-3, IL-4, IL-5, IL-6, IL-7, IL-8, IL-9, IL-10, IL-12p40, IL-12p70, IL-13, IL-15, IL-17, IP-10, MCP-1, MCP-3, MDC, MIP-1α, MIP-1β, sCD40L, TGF-β, TNF-α, TNF-β, VEGF	Serum	Milliplex MAP human cytokines/chemokines premixed 38 plex kit (pg/mL)	35	HC (*n =* 38)	No	Higher in NPSLE vs. HC: EOTA, FRAC, G-CSF, GM-CSF, IFN-α2, IFN-γ, IL-1, IL-1α, IL-1β, IL-1Ra, IL-2, IL-5, IL-6, IL-7, IL-8, IL-9, IL-10, IL-12p40, IL-12p70, IL-13, IL-15, IL-17, IP-10, MCP-1, MCP-3, MDC
Hoang/2022 (29)	Anti-SBSN (suprabasin) antibodies (IgG)	Serum	Luciferase immunoprecipitation system (LIPS) (relative concentration)	35	Viral meningitis (*n =* 20) MS (*n =* 16)	No	Higher in NPSLE vs. viral meningitis (*p =* 0.011) and MS (*p =* 0.039) No difference in levels between NPSLE and non-NPSLE Useful to predict development of NPSLE based on Bayesian model
Hopia/2011 (57)	APRIL BAFF	Plasma CSF	ELISA (ng/mL)	28	30 OND (*n =* 17) MS (*n =* 13)	No	CSF APRIL elevated in NPSLE vs. OND (*p* < 0.01). BAFF CSF tended to be higher in NPSLE (*p* = 0.08) No difference in plasma APRIL levels in NPSLE vs. OND, but BAFF was higher in NPSLE (*p* < 0.05) No CSF or plasma APRIL and BAFF difference NPSLE vs. MS
How/1985 (16)	Anti-neuronal antibody (IgG)	Serum	Mixed hemadsorption assay Neuroblastoma cell line (SK-N-SH) binding (+ binding activity ≥1:20) Glioblastoma cell line (LN229) (+ binding activity ≥1:20)	33	77 RA (*n =* 25), psychiatric disease not connective tissue disease (*n =* 12), non-SLE autoimmune (*n =* 15), HC (*n =* 25)	No	NPSLE had higher neuroblastoma-binding frequency than controls (43% vs. 12%, *p* ≤ 0.0005)
Ichinose/2015 (56)	IL-1b, IL-1ra, IL-2, -4, -5, -6, -7, -8, -9, -10, -12 (p70), -13, -15, and -17A, FGF-b, eotaxin, G-CSF, GM-CSF, IFN-γ, IP-10, MCP-, MIP-1a, MIP-1β, PDGF, RANTES, TNF-α, and VEGF	CSF	Bio-Plex Human 27-plex (pg/mL)	32	42 MS (*n =* 20) NMO (*n =* 22)	No	Significantly higher in NPSLE CSF vs. MS/NMO: IL-2, -5, -7, -9, -10, -12(p70), -13, -15, -17, FGF-b, IFN-γ, GM-CSF, TNF-α, and eotaxine No differences between groups: IL-1b, -1ra, -4, -6, -8, G-CSF, IP-10, MCP-1, MIP-1a, MIP-1β, PDGF, RANTES, and VEGF A six-marker panel (IL-17, IL-2, IFN-γ, IL-5, FGF-b, IL-15) best distinguished NPSLE from MS/NMO with ~95% accuracy
Kwiecinski/2009 (25)	PAI-1 (plasminogen activator inhibitor-1), D-dimer, urokinase, and tissue plasminogen activator (uPA and tPA)	CSF	ELISA (ng/mL)	33	HC (*n =* 53)	No	CSF PAI-1 significantly higher in NPSLE vs. HC (*P* < 0.001) D-dimer and uPA no difference tPA not detectable in CSF
Lu/2010 (24)	IL-1b, IL-2, IL-4, IL-6, IL-8, IL-10, IL-12, IL-17, IP-10, IP-selectin, RANTES, MCP-1, TNF-α, IFN-γ, VCAM−1	CSF	Fluorescent bead-based assay/Luminex (pg/mL)	54	SLE with CNS infections (*n =* 16) non-inflammatory CNS diseases (*n =* 18)^b^	No	Not significantly different among groups: IL-1b, IL-2, IL-4, IL-10, IL-12, MCP-1, RANTES, TNF-α, and IFN-γ Higher in SLE with CNS infections vs. NPSLE: IL-6, IL-8, IL-17, IP-10 Higher in NPSLE vs. non-inflammatory CNS disease: P-selectin, IL-8, VCAM-1
Ogawa/2016 (58)	Anti-N-methyl-D-aspartate receptor (murine; mNR1) Anti-AA residues 19–44 from the N-terminus of NR1 (NR1-A), 37–62 (NR1-B), 56–81 (NR1-C), 75–100 (NR1-D) (IgG)	Serum CSF	ELISA (OD)	41	Various rheumatic diseases other than SLE (non-SLERD, *n =* 21)	Diffuse NPSLE (*n =* 22) Focal NPSLE (*n =* 19)	Serum anti-mNR1, anti-NR1-A, and anti-NR1-C levels were significantly higher in NPSLE than in non-SLERD Anti-NR1-A and anti-NR1-C levels in CSF were significantly elevated in diffuse NPSLE compared vs. focal NPSLE and tween subgroups
Schenatto/2006 (49)	S100B	Serum	Luminescence immunoassay (ng/mL)	23	HC (*n =* 25)	No	Higher S100B levels in NPSLE (*p* < 0.01)
Svenungsson/2001 (54)	Nitric oxide metabolites (NO*_x_*)	CSF	Capillary electrophoresis (μmol/L)	21	HC (*n =* 6)	No	Significantly elevated in NPSLE vs. HC (*p* < 0.0003)
Trysberg/2004 (23)	Tau	CSF	ELISA (pg/mL)	36	HC (*n =* 53)	No	Numerically higher in NPSLE vs. HC, not statistically significant
Trysberg/2004 (34)	MMP-2 MMP-9	CSF	Activity assay/enzymatically active forms (pg/mL)	43	HC (*n =* 22)	No	MMP-2: no difference MMP-9: elevated in NPSLE vs. HC (*p =* 0.0012)
Ushigusa/2016 (11)	α-Klotho	Serum CSF	ELISA (pg/mL)	34	Viral meningitis (*n =* 19) MS (*n =* 20) NMO (*n =* 20)	No	Lower in CSF and serum in NPSLE vs. viral meningitis (*p* < 0.05) Lower serum α-Klotho in NPSLE vs. MS and NMO (*P* < 0.005)
Vanarsa/2022 (55)	NGAL (lipocalin-2), IgM, M-CSF, C3, and other 1,125 proteins screened	CSF	Aptamer-based proteomic platform (1,129-protein screen) with ELISA validation	25 (Halifax cohort) 17 (Hong Kong—validation cohort)	Halifax: non-SLE neurologic disease controls (54; MS and mixed neurologic disorders) Hong Kong: HC (15)	No	Aptamer screen identified 40 proteins elevated in NPSLE CSF; ELISA confirmed a 10-protein panel (including IgM, lipocalin-2, M-CSF, C3). In Halifax, IgM and M-CSF best discriminated NPSLE from neurologic controls (AUC 0.95 and 0.91); in Hong Kong, IgM (AUC 0.78) and lipocalin-2 (AUC 0.85) were most discriminatory, and several markers (e.g. IgM, C3, SERPING1) showed high specificity/PPV. Elevated CSF C3 was associated with ACS; 11 NPSLE-elevated proteins also showed concordant upregulation in choroid plexus
Xu-yan/2008 (50)	S100B	Serum CSF	ELISA (μg/L)	65	HC (*n =* 20)	HC vs. OBS (*n =* 6), seizures (*n =* 11), CVD (*n =* 9), psychosis (*n =* 15), Headache (*n =* 13), neuropathy (*n =* 11)	Higher serum and CSF levels in NPSLE vs. HC (*p* = 0.005) Serum and CSF levels were higher in all individual NPSLE subgroups vs. HC (*p* < 0.001)

MS, multiple sclerosis; OND, other neurological diseases; NMO, neuromyelitis optical; OBS, organic brain syndrome; CVD, cerebral vascular accident.

^a^ Subgroup analysis indicates additional stratification beyond the primary comparison (e.g., psychosis vs. other NP, MRI subgroups, and attribution models). For many Cox analyses, the exact patient number in each risk set was not fixed and is therefore marked qualitatively.

^b^ Non-inflammatory CNS diseases: epilepsy in two, cerebrovascular disease in five, migraine in five, spinal stenosis in one, leukodystrophy in one, myelopathy in one, and mild head trauma in three patients (24).

Overall, the studies in **Table 4** point to broad inflammatory activation in NPSLE compared with HC. CSF IL-6, IL-10, and TNF-α were higher in NPSLE in up to three independent studies, and IL-8, MCP-1, IP-10, RANTES, and MIG were also increased in at least one CSF study (37, 52, 53). In serum/plasma, a large multiplex study reported higher levels of IFN-γ, MCP-1, IP-10, eotaxin, GM-CSF, G-CSF, and multiple interleukins (IL-1α/β, IL-2, IL-5, IL-7, IL-9, IL-10, IL-12p40/p70, IL-13, IL-15, and IL-17) in NPSLE versus HC (29). The components of the kallikrein–kinin system (prekallikrein, kallikrein, kininase II activity, and high- and low-molecular-weight kininogen) were also elevated in both plasma and CSF in NPSLE compared with HC (52).

Evidence for markers of neuronal or vascular injury was more limited but generally pointed in the same direction. Two studies reported higher serum S100B levels in NPSLE than in HC (49, 50), with one study also showing elevated S100B in patients with organic brain syndrome, seizures, cerebrovascular disease (CVD), and psychosis compared with controls (50). In CSF, individual studies found higher MMP-9, PAI-1, and nitric oxide metabolites (NO*_x_*) in NPSLE than in HC (25, 34, 54). By contrast, CSF tau and CSF S100B were either only numerically increased or not consistently different from controls (23, 50).

Proteomic work further supports an inflammatory–vascular CSF signature. An aptamer-based screen identified 40 proteins enriched in NPSLE CSF, and ELISA validation confirmed a 10-protein panel (including IgM, lipocalin-2, M-CSF, and C3) that discriminated NPSLE from neurologic and HC with AUCs up to 0.95; elevated CSF C3 was also linked to ACS (55). However, the two cohorts’ sample sizes (discovery and validation) assessed in this study were small (24 and 17 NPSLE, respectively); therefore, these findings require independent replication.

In MS/NMO comparisons, a single CSF multiplex study (56) found that several cytokines elevated in NPSLE versus HC (including IL-2, IL-5, IL-7, IL-9, IL-10, IL-12p70, IL-13, IL-15, IL-17, IFN-γ, GM-CSF, TNF-α, FGF-b, and eotaxin) were also higher in NPSLE than in MS/NMO, and a six-marker panel (IL-17, IL-2, IFN-γ, IL-5, FGF-b, and IL-15) discriminated NPSLE from MS/NMO with ~95% accuracy (56). Another CSF study reported higher P-selectin, VCAM-1, and IL-8 in NPSLE than in non-inflammatory CNS disease (*n =* 18, combination of subjects with epilepsy, CVD, spinal stenosis, leukodystrophy, myelopathy, and mild head trauma), but no differences in IL-1β, IL-2, IL-4, IL-10, IL-12, MCP-1, RANTES, TNF-α, and IFN-γ 30 (24).

For B-cell-related factors and autoAb, CSF APRIL (but not BAFF) and plasma BAFF were higher in NPSLE than in non-SLE neurological controls (57), and serum anti-neuronal Ab (16) and several NMDA-receptor-related Ab (anti-mNR1, anti-NR1-A, and anti-NR1-C) were more frequent or higher in NPSLE than in non-SLE rheumatic diseases, particularly in diffuse NPSLE (58). By contrast, anti-NR1/NR2 antibodies were rarely positive and added little beyond anti-NR2 (59).

Anti-suprabasin (SBSN) Ab were higher in NPSLE than in viral meningitis and MS and contributed to the prediction of incident NPSLE in a Bayesian model (29). In CSF, several inflammatory mediators that were increased in NPSLE versus HC—including IL-6, IL-8, IL-17, MIG, IP-10, MCP-1, and RANTES—were even more elevated in SLE with infectious meningitis/encephalitis, producing a gradient of infection > NPSLE > HC for these markers (37). Finally, the α-Klotho levels in CSF were lower in NPSLE than in viral meningitis, MS, or NMO, suggesting a relative depletion of this neuroprotective factor in NPSLE (11).

#### Narrative synthesis for analytes studied as prognostic and monitoring biomarkers in NPSLE

This section summarizes analytes that have been evaluated as prognostic markers or longitudinal monitoring tools in NPSLE. These prognostic and longitudinal studies were not pooled because for every analyte × matrix × outcome combination there were fewer than five independent studies. The included reports either related baseline levels to subsequent outcomes (e.g., relapse, poor vs. good prognosis, mortality) or examined within-patient changes over time (e.g., pre- vs. post-treatment). Their findings are summarized in **Table 5**. The results are presented at the study level, reflecting substantial heterogeneity in assays, sampling schedules, treatments, and outcome definitions.

**Table 5:** Longitudinal studies evaluating analytes as prognostic or monitoring biomarkers in NPSLE.

Study	Analyte(s)	Matrix	Technique	Population/exposure (*n*)	Outcome/comparison	Main findings
Ichinose/2016 (60)	27-plex cytokine/chemokine panel (incl. IL-6, IL-10, IFN-γ, TNF-α)	CSF	Multiplex bead assay	NPSLE (*n =* 27): responders (*n =* 11) vs. non-responders (*n =* 16)	Clinical response at 1 year	Significantly higher baseline IL-6, IL-10, IFN-γ, and TNF-α (*p* = 0.02, *p =* 0.005, *p =* 0.024, and *p =* 0.007, respectively) in non-responders; no prognostic signal for other cytokines
Fragoso-Loyo/2007 (37)	IL-6, IL-8, MCP-1, IP-10, MIG (±low/undetectable IL-2, IL-4, IL-10, TNF-α, IFN-γ)	CSF	Cytometric bead array	NPSLE (*n =* 30): baseline vs. 6-month follow-up	Pre- vs. post-treatment change	IL-6, IL-8, MCP-1, IP-10 and MIG were significantly lower at 6 months (*p =* 0.0001, *p =* 0.001, *p =* 0.002, *p =* 0.0001, respectively)
Lu/2010 (24)	IL-6, IL-8, MCP-1, P-selectin, IP-10, VCAM-1 (± IL-1, IL-4, IL-10, IL-17, TNF-α, IFN-γ, RANTES)	CSF	Luminex bead assay	NPSLE (*n =* 21): baseline vs. post-induction	Pre- vs. post-treatment change	IL-6 (*p* < 0.01), IL-8 (*p* < 0.001), MCP-1 (*p* < 0.05), P-selectin (*p* < 0.05), and VCAM-1 (*p* < 0.01) after induction; other cytokines/chemokines showed no significant change
Pang/2024 (46)	BAFF	Serum	ELISA	NPSLE (*n =* 105): high BAFF (≥428 pg/mL, *n =* 52) vs. low BAFF (*n =* 53)	Relapse or death over 24 months	High baseline BAFF predicted greater risk of relapse or death (HR 2.50, 95% CI 1.19–5.24)
Arinuma/2019 (61)	Anti-ribosomal P (IgG)	Serum	ELISA	Diffuse NPSLE (*n =* 55)	Overall and 1-year mortality	Anti-RibP positivity at onset independently predicted higher overall and early mortality compared with anti-RibP-negative patients (RR 2.26 (95% CI 1.28–4.42), *p* < 0.005)
Ding/2022 (62)	Anti-ribosomal P (IgG)	Serum	Immunoblot (P0, P1, P2)	SLE (*n =* 2,395); anti-RibP^+^ *n =* 659	Anti-RibP^+^ vs. anti-RibP^-^	Anti-RibP^+^ patients accrued more NP damage (adjusted HR = 3.8, 95% CI 2.7–57, *p* < 0.001) and were more likely to develop NP events (log-rank test: *p* < 0.05)
Hanly/2011 (63)^a^	LA, aCL, anti-β2GPI, anti-RibP, anti-NR2	Plasma/serum	Coagulation assay; ELISA	SLE inception cohort (*n =* 1,047)	Time to first NP event and NP attributed to SLE	Baseline autoAb did not predict overall NP events
Hanly/2019 (64)^a^	LA, aCL, anti-β2GPI, anti-RibP, anti-NR2	Plasma/serum	Coagulation assay; ELISA	Inception cohort (*n =* 1,826); psychosis cases *n =* 28	Time to first psychosis attributed to SLE	Anti-RibP (baseline/time-varying) associated with psychosis in univariate analyses, but no antibody independently predicted psychosis after multivariable adjustment
Mikdashi/2005 (65)	LA, aCL IgG, anti-Sm IgG	Plasma/serum	LA: phospholipid-dependent coagulation assay/aCL : ELISA	SLE (*n =* 195); seizures *n =* 28	Time to first seizure and to epilepsy	High-titer IgG aCL (OR = 3.7, 95% CI 1.3–9.5, *p =* 0.03), LA (OR = 6.2, 95% CI 1.7–22.5, *p =* 0.037), and anti-Sm (OR = 3.3, 95% CI 1.2–9, *p =* 0.007) independently predicted any seizure; IgG aCL associated with earlier seizure onset (HR = 2.23, 95% CI 1.01–4.67, *p =* 0.034); no robust predictor of epilepsy
Appenzeller/2008 (66)	aPL (aCL IgG/IgM, LA)	Plasma/serum	LA: phospholipid-dependent coagulation assay/aCL : ELISA	SLE (*n =* 520); SLE psychosis *n =* 40, steroid-induced psychosis *n =* 28	Psychosis vs. no psychosis; SLE-related vs. steroid-induced psychosis	Moderate-high-titer aPL risk factor for psychosis (OR = 3.2, 95% CI 1.9–4.5, *p =* 0.004; multivariable analysis)

^a^ Analyses derived from the same SLICC inception cohort.

Overall, studies evaluating prognostic or evaluative analytes in NPSLE are few and heterogeneous, but some consistent themes emerge. Among CSF biomarkers studied longitudinally, small cohorts suggest that inflammatory cytokines and chemokines mainly behave as activity and treatment-response markers rather than robust prognostic tools (24, 37, 60). In Ichinose et al. (60), higher pretreatment CSF IL-6, IL-10, IFN-γ, and TNF-α distinguished NPSLE non-responders from responders at 1 year, whereas the remaining multiplex analytes showed no signal. In two other independent studies, CSF IL-6, IL-8, and MCP-1 decreased after induction therapy in parallel with clinical improvement (24, 37). CSF IP-10 and MIG were also lower at 6 months post-treatment in Frogoso-Loyo et al. (37), whereas P-selectin and VCAM-1 decreased in Lu et al. (24).

In peripheral blood, one study reported that baseline serum BAFF above the cohort median predicted a higher 24-month risk of relapse or death in NPSLE (46), but this has not yet been independently replicated. Anti-ribosomal P (Ribo P) Ab have been linked to more severe outcomes in some but not all cohorts. In diffuse NPSLE, baseline anti-Ribo P positivity predicted increased overall and early mortality (*n =* 55) (61), and in a larger SLE cohort (*n =* 659) it was associated with greater subsequent NP damage, particularly cognitive and cerebrovascular (62). However, analyses from the SLICC inception cohort (*n =* 1,826) did not confirm anti-Ribo P as an independent predictor of overall NP events or psychosis after adjustment for demographic and clinical factors (63, 64).

Antiphospholipid Ab show outcome-specific but inconsistent associations. lupus anticoagulant (LA) predicted later stroke/sinus thrombosis in one inception cohort (63), and high-titer anticardiolipin (aCL), LA, and anti-Sm were associated with seizures in another (65), while moderate to high-titer aPL increased the risk of SLE-related (but not steroid-induced) psychosis (66). Yet in larger adjusted models, aPL and other Abs generally failed to independently predict NP events (63, 64).

### Studies comparing analyte levels between NPSLE and non-NPSLE patients with data from five or more independent studies

This section summarizes studies comparing analyte levels between NPSLE and non-NPSLE patients with data from five or more independent studies overall. Meta-analyses were performed for studies reporting continuous variables and were conducted separately for serum/plasma and CSF when at least five studies were available for a given analyte within each biofluid. Studies reporting dichotomous data or analytes with fewer than five studies per biofluid were summarized descriptively.

#### MCP-1/CCL2

Pooling the five available studies of CSF MCP-1/CCL2 in NPSLE (*n =* 256) versus non-NPSLE (*n =* 172 SLE) using a random-effects model (Hedges’ *g*, REML) yielded a moderate elevation of CSF MCP-1 in NPSLE (pooled *g* = 0.66, 95% CI 0.41 to 0.90), with low-moderate heterogeneity (*τ*² = 0.02, *I*² ≈ 25%). The 95% prediction interval (0.28 to 1.03) suggests that future similar studies are still likely to observe a positive association, ranging from small to large effects (**Figure 2** shows the random effects results and **Table 6** summarizes the NPSLE manifestations and SLE disease activity scores in each study). Disease activity was reported inconsistently across studies, and none matched or adjusted their analyses for disease activity. Excluding the higher risk, the study of Lu et al. (24) (MCP-1 digitized from a figure) produced very similar results (*k* = 4; g = 0.62, 95% CI 0.34 to 0.91, *I*² ≈ 33%, PI 0.18 to 1.06), indicating that the overall finding is robust to the removal of digitized data. A simple meta-regression by assay class (bead-based multiplex vs. single-plex ELISA) explained ~23% of between-study heterogeneity (*R*² = 23%) but did not identify a statistically significant difference in effect sizes between platforms (ELISA ≈ *g* 0.49 vs. multiplex ≈ *g* 0.75; QM (1) = 1.13, *p =* 0.29). The leave-one-out analyses showed pooled effects ranging from *g* = 0.55 to 0.75, with all 95% CIs remaining above zero and *I*² dropping to 0% only when the largest-effect study (11) was removed, further indicating that no single study or assay drives the observed association between higher CSF MCP-1/CCL2 levels and NPSLE.

**Figure 2:**
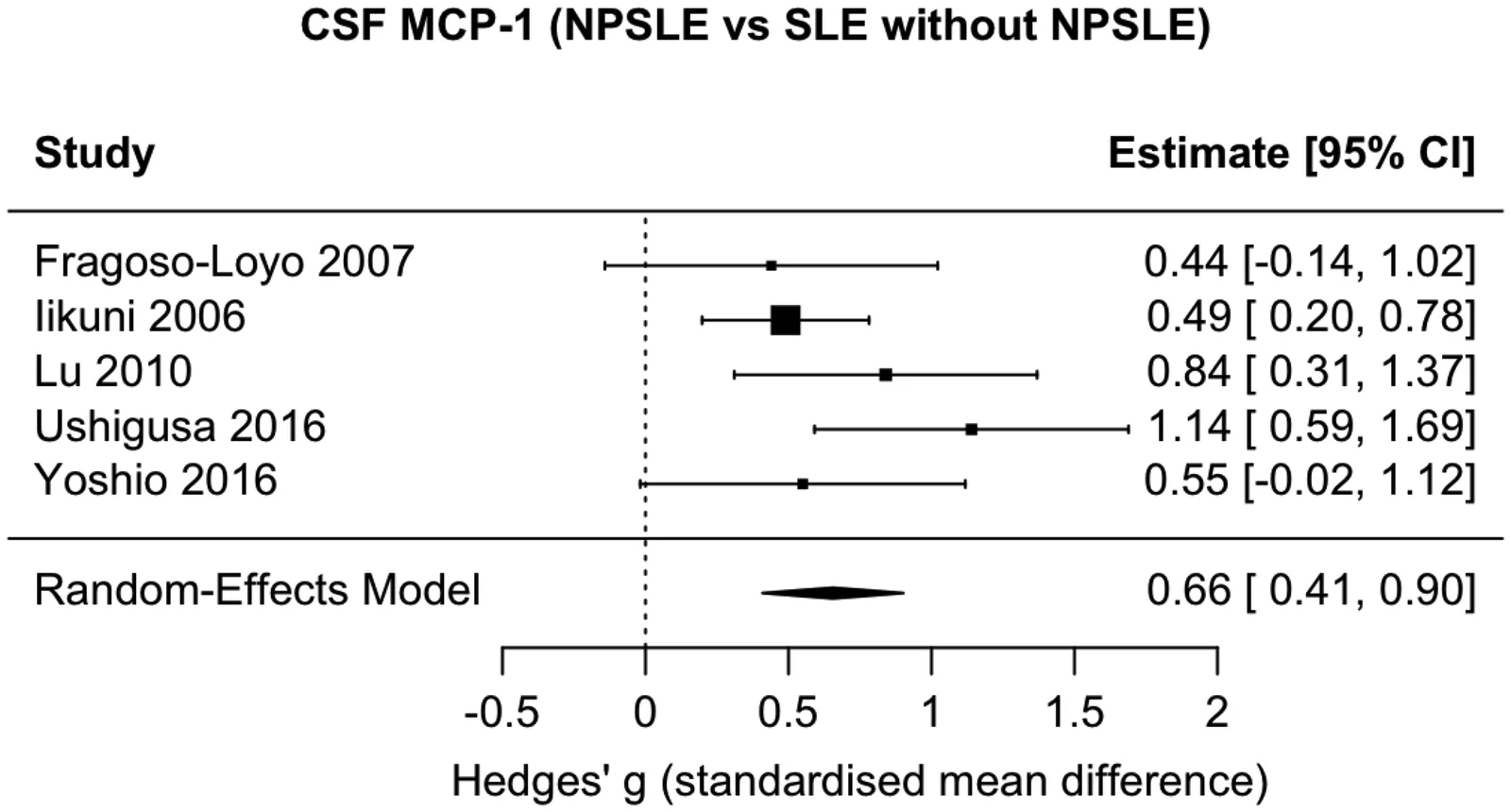
Forest plot of CSF MCP-1 (CCL2) concentrations in NPSLE versus SLE without NPSLE. Effect sizes are standardized mean differences (Hedges’ *g*), with positive values indicating higher MCP-1 levels in NPSLE. Squares show study-specific estimates (size proportional to precision), horizontal lines show the 95% confidence intervals, and the diamond shows the random-effects pooled estimate.

**Table 6:** Studies included in the CSF MCP-1/CCL2 meta-analysis.

Study	*N* NPSLE (total)	Central—diffuse, *n*	Central—focal, *n*	Peripheral, *n*	Disease activity measure	Disease activity in NPSLE vs. comparator	Notes
Fragoso-Loyo/2007 (37)	42	10 (ACS 8, psychosis 2)	28 (seizure disorder 14, severe refractory headache 8, CVD 4, transverse myelitis 1, pseudotumor cerebri 1)	4 (mononeuritis multiplex 3, polyneuropathy 1)	SLEDAI-2K	Higher in NPSLE (14.1 ± 8.7 vs. 7.0 ± 5.9; *p =* 0.013)	One primary NP category per patient
Iikuni/2006 (98)	96	38 (ACS 3, anxiety 5, CI 4, mood disorders 9, psychosis 17)	55 (CVD 16, demyelinating syndrome 2, movement disorder 6, seizures 20, aseptic meningitis 11)	3 (cranial neuropathy 3)	Not reported	Not reported	Primary category per patient reported
Lu/2010 (24)	54	47 (ACS 38, CI 7, psychosis 2)	7 (isolated seizures 3, CVD 2, myelitis 2)	0	BILAG (NPSLE only)	NPSLE patients had BILAG A at enrollment; no direct comparison with non-NPSLE controls	Some ACS patients had additional focal/peripheral manifestations
Ushigusa/2016 (11)	34	21 (psychosis 10, ACS 2, CI 3, mood disorder 6)	7 (CVD 3, seizure disorder 2, headache 2)	6 (polyneuropathy 2, cranial neuropathy 2, mononeuropathy 2)	SELENA-SLEDAI	Median 12 vs. 8; *p =* 0.053	One primary NP category per patient
Yoshio/2016 (47)	30	22 (ACS 16, anxiety 1, mood disorder 4, psychosis 1)	8 (aseptic meningitis 4, CVD 3, myelopathy 1)	0	SLEDAI	Higher in central NPSLE; mean SLEDAI significantly greater than comparator SLE group	Central NPSLE only; one primary NP category per patient

One serum study (29) measured MCP-1 in a 38-plex panel in 35 NPSLE and 34 SLE patients, without showing a clear MCP-1 difference between NPSLE and non-NPSLE SLE. As this is the only serum MCP-1 study, it was not meta-analyzed.

#### IL-8

Five studies evaluated CSF IL-8 in NPSLE (*n =* 191) versus non-NPSLE (*n =* 131). A random-effects meta-analysis suggested higher CSF IL-8 in NPSLE (pooled Hedges’ *g* = 0.60, 95% CI 0.05 to 1.16), but heterogeneity was substantial (*τ*² = 0.32; *I*² = 81%, Q(4) = 21.99, *p =* 0.0002). The 95% prediction interval was wide (-0.64 to 1.85), indicating that future studies could find no difference or even lower IL-8 in NPSLE. Excluding the higher risk, digitized study of Lu et al. (24) yielded a smaller, non-significant effect (*k* = 4; *g* = 0.49, 95% CI -0.16 to 1.13, *p =* 0.14; *I*² = 83%, PI -0.86 to 1.83). In leave-one-out analyses, pooled effects remained positive (*g* = 0.48 to 0.89), but confidence intervals frequently crossed zero, except in the study of Ushigusa et al. (11), which reported slightly lower IL-8 in NPSLE (g ≈ -0.44) and was thus removed. In that case, the summary effect became large and precise (*g* = 0.89, 95% CI 0.62–1.16), with *I*² = 0%. Assay class (ELISA vs. bead-based multiplex) did not explain the heterogeneity (*R*² = 0%; QM (1) = 0.73, *p =* 0.39). **Figure 3** shows the random effects results, and **Table 7** summarizes the NPSLE manifestations in each study.

**Figure 3:**
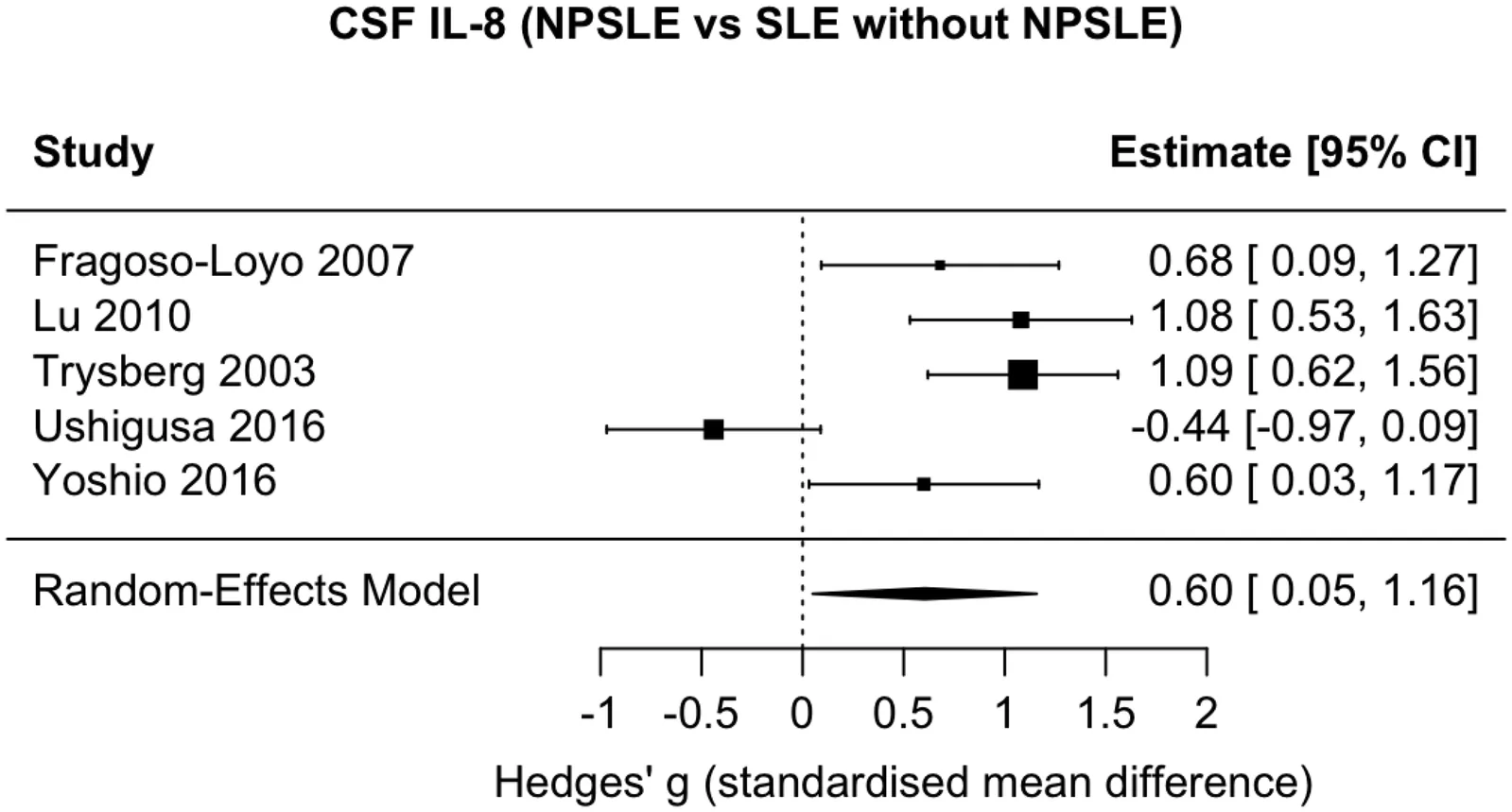
Forest plot of CSF IL-8 concentrations in NPSLE versus SLE without NPSLE. Effect sizes are standardized mean differences (Hedges’ *g*), with positive values indicating higher IL-8 levels in NPSLE. Squares show study-specific estimates (size proportional to precision), horizontal lines show the 95% confidence intervals, and the diamond shows the random-effects pooled estimate.

**Table 7:** Studies included in the CSF IL-8 meta-analysis.

Study	*N* NPSLE (total)	Central—diffuse, *n*	Central—focal, *n*	Peripheral, *n*	Notes
Fragoso-Loyo/2007 (37)	42	10 (ACS 8, psychosis 2)	28 (seizure disorder 14, severe refractory headache 8, CVD 4, transverse myelitis 1, pseudotumor cerebri 1)	4 (mononeuritis multiplex 3, polyneuropathy 1)	One primary NP category per patient
Lu/2010 (24)	54	47 (ACS 38, CI 7, psychosis 2)	7 (isolated seizures 3, CVD 2, myelitis 2)	0	Some ACS patients had additional focal/peripheral manifestations
Trysberg/2003 (18)	31	14 events (CI 6, mood disorders 6, ACS 1, anxiety 1)	23 events (CVD 5, seizure disorders 7, headache 6, demyelinating syndrome 1, movement disorder 2, myelopathy 2)	0	Counts are events; some patients have ≥2 manifestations
Ushigusa/2016 (11)	34	21 (psychosis 10, ACS 2, CI 3, mood disorder 6)	7 (CVD 3, seizure disorder 2, headache 2)	6 (polyneuropathy 2, cranial neuropathy 2, mononeuropathy 2)	One primary NP category per patient
Yoshio/2016 (47)	30	22 (ACS 16, anxiety 1, mood disorder 4, psychosis 1)	8 (aseptic meningitis 4, CVD 3, myelopathy 1)	0	Central NPSLE only; one primary NP category per patient

Only two studies assessed circulating IL-8 in relation to NPSLE. Dellalibera-Joviliano et al. (52) measured plasma IL-8 in active NPSLE versus HC and found no clear difference between the levels, aligning with their observation that IL-8 was largely undetectable in CSF. Hoang et al. (29) included serum IL-8 in a multiplex panel comparing NPSLE with non-NPSLE (and HC) and reported very similar concentrations across groups, with no significant NPSLE vs. non-NPSLE differences.

#### IL-6

##### IL-6 in CSF (reported as continuous levels)

Seven studies contributed continuous CSF IL-6 data in NPSLE (*n =* 251) versus non-NPSLE (*n =* 260) across a mix of diffuse (ACS, psychosis, mood and cognitive disorders; **Table 8**) and focal syndromes (seizures, CVD, myelopathy, aseptic meningitis, severe headache; **Table 8**), with peripheral neuropathies being rare. Disease activity was reported inconsistently across studies (**Table 8**); none matched participants on disease activity, and only one study (Shi et al.) (67), adjusted its analyses for disease activity, finding that CSF IL-6 was no longer independently associated with NPSLE after adjustment.

**Table 8:** Studies included in the CSF IL-6 continuous data meta-analysis.

Study	*N* NPSLE (total)	Central—diffuse, *n*	Central—focal, *n*	Peripheral, *n*	Disease activity	Disease activity in NPSLE vs. comparator	Notes
Asano/2017 (99)	48	30 (ACS 14, psychosis 10, mood 5, CI 1)	18 (no further subdivision given)	0	Not reported	Not reported	One primary NP category per patient. IL-6 available for 47/48, but phenotype counts use full NPSLE group
Fragoso-Loyo/2007 (37)	42	10 (ACS 8, psychosis 2)	28 (seizures 14, severe headache 8, CVD 4, transverse myelitis 1, pseudotumor cerebri 1)	4 (mononeuritis multiplex 3, polyneuropathy 1)	SLEDAI-2K	Higher in NPSLE (14.1 ± 8.7 vs. 7.0 ± 5.9; *p =* 0.013)	One primary NP category per patient.
George-Chandy/2008 (22)	32	17 (ACS 2, anxiety 1, CI 4, mood 3, psychosis 7)	15 (aseptic meningitis 1, CVD 6, demyelinating 4, headache 3, myelopathy 1)	0	Not reported	Not reported	One primary NP category per patient
Trysberg/2003 (18)	31	14 events (CI 6, mood 6, ACS 1, anxiety 1)	23 events (CVD 5, seizures 7, headache 6, demyelinating 1, movement disorder 2, myelopathy 2)	0	Not reported	Not reported	Counts are events; some patients have multiple manifestations
Ushigusa/2016 (11)	34	21 (psychosis 10, ACS 2, CI 3, mood 6)	7 (CVD 3, seizures 2, headache 2)	6 (polyneuropathy 2, cranial neuropathy 2, mononeuropathy 2)	SELENA-SLEDAI	Median in NPSLE 12 vs. 8; *p =* 0.053	One primary NP category per patient
Yoshio/2016 (47)	30	22 (ACS 16, anxiety 1, mood 4, psychosis 1)	8 (aseptic meningitis 4, CVD 3, myelopathy 1)	0	SLEDAI	Higher in NPSLE (16.1 ± 8.25 vs. 10.9 ± 7.89; *p =* 0.003)	Central NPSLE only; one primary NP category per patient
Shi/2023 (67)	30	22 events (anxiety 7, CI 4, mood 9, psychosis 2)	17 events (headache 11, seizures 4, CVD 2)	0	SLEDAI	Higher in NPSLE (median 10 vs. 4; *p* < 0.001)	Counts are events; some patients have multiple manifestations

In a random-effects model of these studies, the CSF IL-6 levels were substantially higher in NPSLE than in non-NPSLE, with a pooled Hedges’ *g* of 0.81 (95% CI 0.54 to 1.07). Between-study heterogeneity was moderate (*τ*² = 0.05; *I*² = 42%; Q(6) = 10.0, *p =* 0.12), and the 95% prediction interval (0.29 to 1.32) indicated that most future studies would be expected to show a positive association ranging from small to large effects. The assay platform (cell-based bioassay, bead-based multiplex, chemiluminescent immunoassay, and single-plex ELISA) did not explain heterogeneity (*R*² ≈ 0%; QM (3) = 0.34, *p =* 0.95). The leave-one-out analyses yielded pooled effects between *g* = 0.71 and 0.90, all with 95% CIs above zero and *I*² between ~16% and ~49%, indicating that no single study or assay type fully accounted for the association of higher CSF IL-6 with NPSLE. The forest plot of these findings is presented in **Figure 4**.

**Figure 4:**
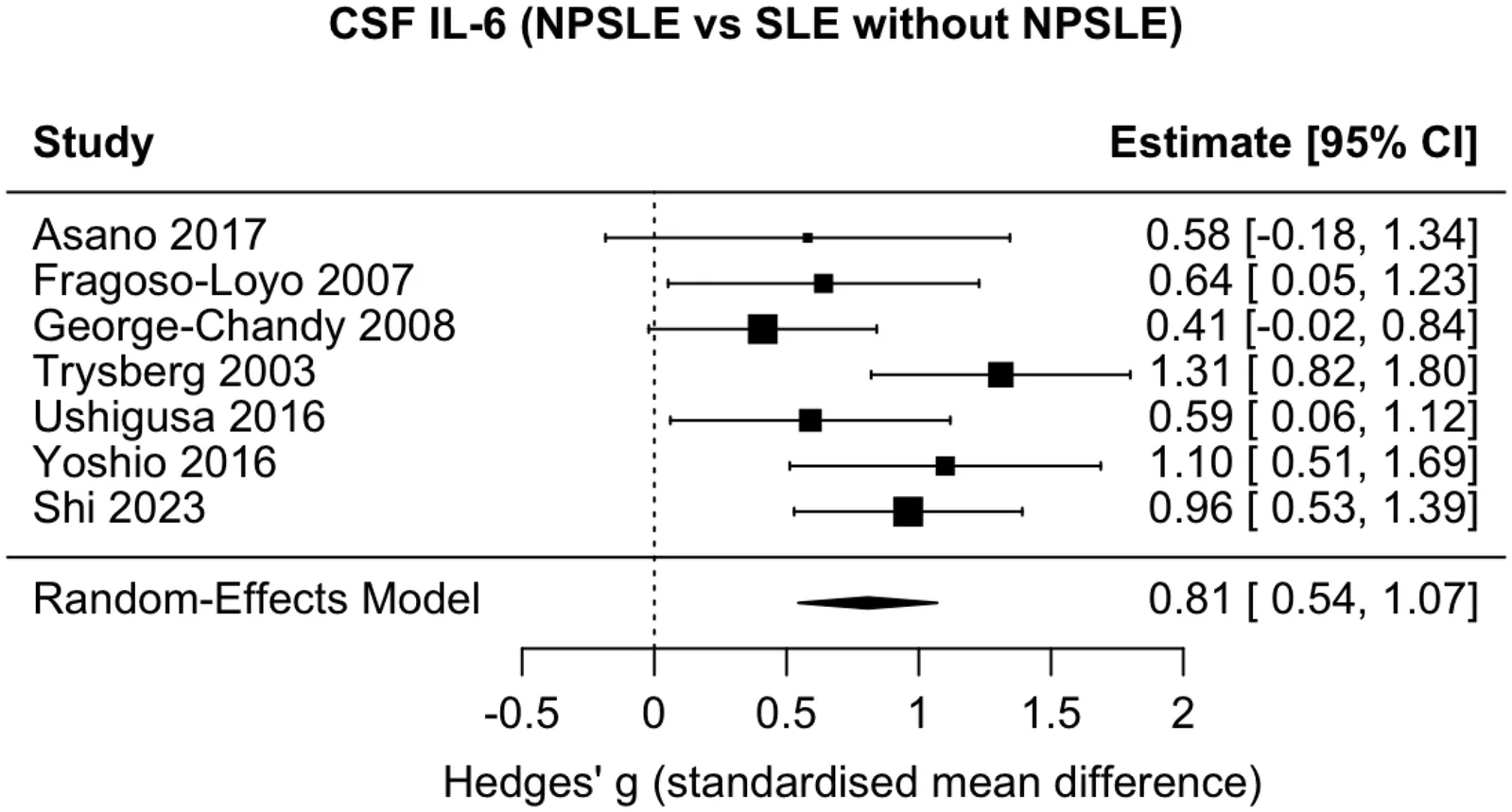
Forest plot of CSF IL-6 concentrations in NPSLE versus SLE without NPSLE. Effect sizes are standardized mean differences (Hedges’ *g*), with positive values indicating higher IL-6 levels in NPSLE. Squares show study-specific estimates (size proportional to precision), horizontal lines show the 95% confidence intervals, and the diamond shows the random-effects pooled estimate.

##### IL-6 in CSF (dichotomous data)

Two studies reported CSF IL-6 only as “elevated” versus “normal” using assay-specific cutoffs, without continuous values. In the study of Hirohata et al. (68) among SLE patients with psychiatric manifestations, IL-6 above 4.3 pg/mL was detected in 28 of 32 lupus psychosis cases but in only one of 13 non-lupus psychosis controls, yielding a very large but highly imprecise odds ratio (OR ≈ 84, 95% CI ≈ 8 to 830) driven by this single positive control. In contrast, Jönsen et al. (36) defined elevated CSF IL-6 as >15 ng/L and found it in three of 28 NPSLE cases and one of 14 non-NPSLE SLE controls (OR ≈ 1.6 with very wide confidence intervals).

##### IL-6 serum

Three studies provided continuous serum IL-6 data in relation to neuropsychiatric involvement, comprising 176 NPSLE patients and 294 non-NPSLE. Two cohorts (De Sousa et al. and Muñoz-Grajales et al.) (30, 32) examined serum IL-6 levels in SLE patients stratified by cognitive function and specific cognitive domains. De Sousa et al. found higher IL-6 levels in patients with impaired attention (*p =* 0.031), while our group observed significantly elevated levels in patients with impairments in both simple attention and processing speed (*p* < 0.01 for both) (30, 32). In contrast, Shi et al. (67), which directly compared various NPSLE manifestations with non-NPSLE, reported very similar serum IL-6 distributions (medians 5.0 vs. 4.7 pg/mL) and no significant difference. Similarly, Jönsen et al. (36), which reported dichotomous data, found elevated serum IL-6 in one of 28 NPSLE versus two of 16 non-NPSLE patients (OR ≈ 0.3, with wide confidence intervals). In aggregate, while these reports do not suggest a consistent association between serum IL-6 and NPSLE, elevated IL-6 serum levels may be associated more specifically with CI.

#### Anti-NR2/NMDAR

##### Serum anti-NR2

Five studies contributed data for the serum anti-NR2 or anti-NMDAR Ab meta-analysis (20, 26, 69, 70, 71) representing 255 NPSLE and 252 non-NPSLE (**Table 9**). In the studies of Gono (69), Hanly (20), and Mader (26), ORs were reported directly; in the studies of Harrison (70) and Fragoso-Loyo (71), we reconstructed ORs from the reported numbers of anti-NR2-positive and anti-NR2-negative patients in each group. Using a random-effects model with the studies, anti-NR2 positivity was associated with higher odds of NPSLE compared with non-NPSLE (pooled logOR 0.74; OR 2.09, 95% CI 1.07–4.06; **Figure 5**). Between-study heterogeneity was moderate (*τ*² = 0.34; *I*² ≈ 59%), and the 95% prediction interval was wide (OR 0.55 to 7.86), so individual future studies could still observe weak or null associations. Assay type (ELISA vs. multiplex bead assay) did not explain this heterogeneity (*R*² ≈ 0%; QM(1) = 0.98, *p =* 0.32). The leave-one-out sensitivity analyses yielded pooled odds ratios between 1.66 and 2.54, consistently favoring higher serum anti-NR2 Ab positivity in NPSLE versus SLE without NPSLE. However, the statistical significance of the pooled effect was attenuated when individual studies were omitted (e.g., *p* ≈ 0.15 when the study of Mader et al. (26) was excluded), indicating that while the direction of association was stable, the strength of evidence was moderately sensitive to the inclusion of specific studies.

**Table 9:** Studies included in the anti-NR2/NMDAR meta-analysis.

Study	*N* NPSLE (total)	Central—diffuse, *n*	Central—focal, *n*	Peripheral, *n*	Notes
Gono/2011 (69)	24	12 events (not discriminated)	12 events (not discriminated)	7 events (not discriminated)	Counts are events; some patients have multiple manifestations
Hanly/2022 (20)	47	47 (CI)	No	No	Focus on CI; some patients also had prior NP events
Harrison/2006 (70)	48	47 (CI)	No	No	Restricted to CI; no focal CNS or PNS NPSLE reported
Mader/2018 (26)	108	NR (mixed diffuse and focal CNS not broken down)	NR includes 11 demyelinating (mixed diffuse and focal CNS not broken down)	No	ACR-defined NPSLE; 11 demyelinating CNS cases reported, other diffuse/focal/PNS phenotypes not stratified
Fragoso-Loyo/2008 (71)	47	9 (ACS 8, psychosis 1)	34 (seizure disorders 16, severe refractory headache 9, CVD 7, pseudotumor cerebri 1, transverse myelitis 1)	4 (mononeuritis multiplex 3, polyneuropathy 1)	One category per patient

**Figure 5:**
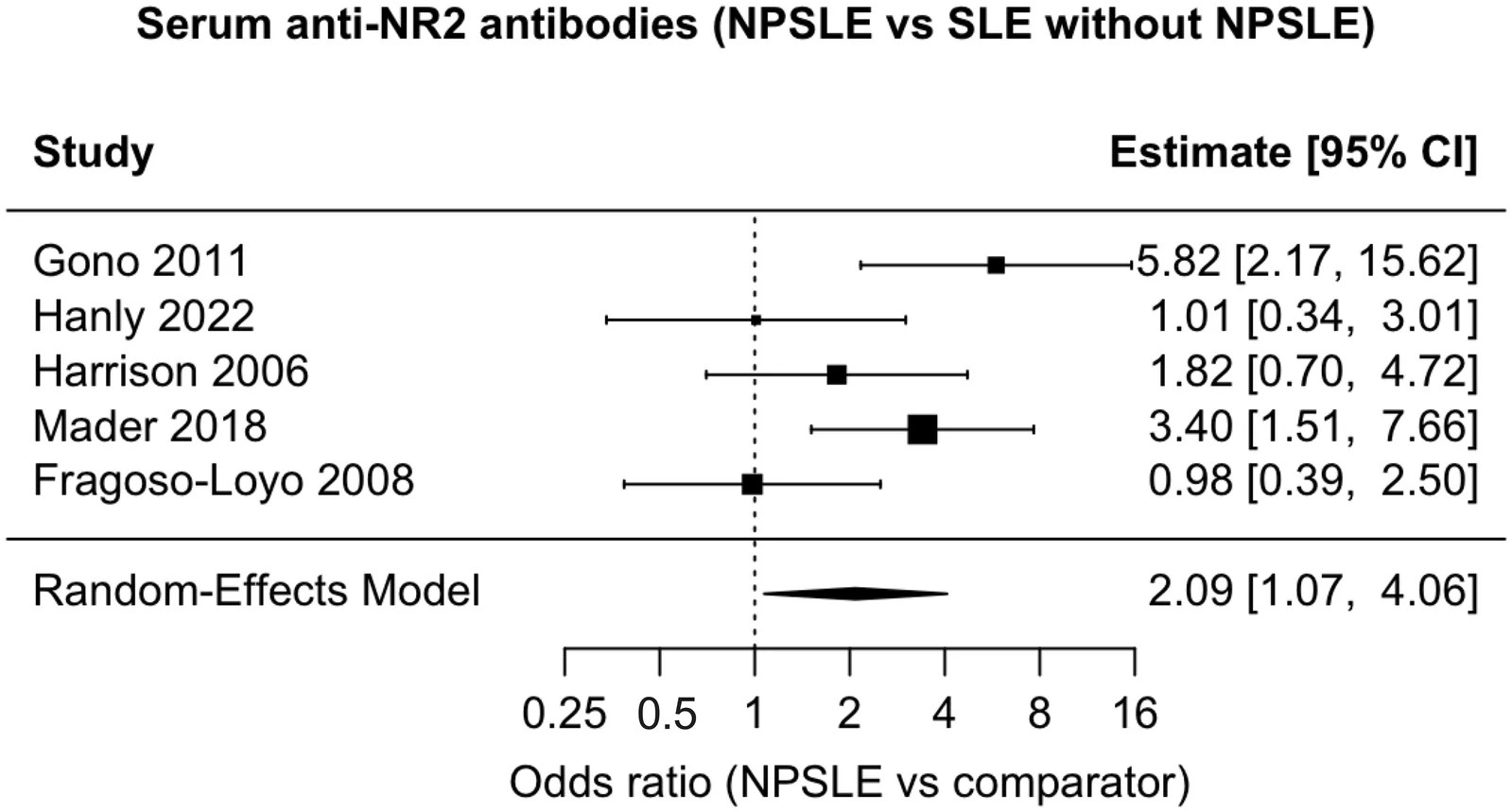
Forest plot of serum anti-NR2 antibodies in NPSLE versus SLE without NPSLE. Effect sizes are expressed as odds ratios (from log odds ratios, plotted on a logarithmic scale), with squares indicating study-specific estimates (size proportional to precision), horizontal lines indicating the 95% confidence intervals, and the diamond indicating the random-effects pooled estimate.

Lapteva et al. (72), which compared SLE patients with and without global CI, also measured serum IgG anti-NR2 Ab by ELISA and related Ab titers (absent/low/high) to a continuous global cognitive dysfunction score. Because the results were presented only as titers versus continuous scores rather than counts of positive/negative in each group, no extractable 2 × 2 data were available for our meta-analysis; the authors reported no association between anti-NR2 levels and global CI (72).

##### CSF anti-NR2 (dichotomous data)

Three studies (71, 73, 74) reported CSF anti-NR2/NMDAR as “positive” versus “negative” using assay-specific cutoffs rather than continuous levels. CSF positivity was consistently more frequent in NPSLE than in comparison groups, but the samples were small, the comparators were varied (surgical SLE, non-SLE neurologic controls, and septic meningitis), and only one study included an SLE non-NPSLE group. With *k* = 3 and this marked clinical/methodological heterogeneity, we did not meta-analyze them. In Fragoso-Loyo et al. (71), CSF anti-NMDAR was present in 14 of 35 (40%) central NPSLE, none of the five peripheral NPSLE, one of 16 (6%) surgical SLE, and one of 17 (6%) non-autoimmune controls, but in all five (100%) SLE patients with septic meningitis. Arinuma et al. (73) reported anti-NR2 above the cutoff in 31 of 38 (81.6%) diffuse and eight of 18 (44.4%) focal NPSLE versus none of the 20 neurologic controls (*χ*² *p =* 0.012), with serum anti-NR2 showing no diagnostic value. Hirohata et al. (74) found significantly higher CSF anti-NR2 levels and intrathecal index in 81 NPSLE patients compared with 22 non-SLE controls, peaking in ACS and correlating with BBB damage (Q-albumin *r* = 0.38, *p =* 0.0053).

Taken together, these three small studies suggest that CSF anti-NR2/NMDAR Abs are enriched in NPSLE, particularly central diffuse/ACS forms, and relate to BBB damage. However, because of very small, heterogeneous samples, the evidence remains exploratory.

#### Anti-Ribo P

For anti-Ribo P, 11 studies (Abdel-Nasser (75), Briani (76), Schneebaum (77), Hanly 2008 (78), Taha (79), Arnett (80), Isshi-Hirohat (81), Jönsen (36), Seth (39), Hanly 2022 (20), and Huang (48); refer to **Table 10**) provided extractable case–control data comparing serum anti-Ribo P levels between ACR-defined NPSLE (or NPSLE subsets; *n =* 628) and non-NPSLE (*n =* 1,660). In the random-effects meta-analysis of these studies (**Figure 6A**), serum anti-Ribo P Abs were significantly associated with NPSLE. The pooled log odds ratio was 0.98 (SE = 0.14, *z* = 6.94, *p* < 0.0001), corresponding to an OR of 2.68 (95% CI 2.03 to 3.53). The 95% prediction interval on the OR scale (2.03 to 3.53) was identical to the CI, reflecting the near-zero between-study variance estimated by the random-effects model. Between-study heterogeneity was not statistically detectable (*τ*² ≈ 0, *I*² = 0%, Q[10] = 9.52, *p =* 0.48). In meta-regression, assay class did not explain additional variability (QM[3] = 4.89, *p =* 0.18), and the heterogeneity estimate remained. The leave-one-out analyses produced highly similar pooled log ORs (≈ 0.94–1.06, all *p* < 0.001) with unchanged heterogeneity indices, indicating that no single study—including Hanly et al. (20), which used a neuropsychological definition of CI versus normal cognition—drove the overall result. Excluding the three psychosis-focused studies (*k* = 7) did not weaken the association either. The pooled log odds ratio was 0.90 (SE = 0.16, *z* = 5.52, *p* < 0.0001), corresponding to an OR of 2.47 (95% CI 1.79 to 3.41), with a prediction interval (1.79 to 3.41) that again overlapped the confidence interval. Heterogeneity remained undetectable (*τ*² ≈ 0, *I*² = 0%), assay class was not a significant modifier, and leave-one-out analyses yielded nearly identical estimates (log OR ≈ 0.87 to 0.99, all *p* < 0.001). Similarly, the funnel plot was broadly symmetrical, without obvious outliers or a lack of small neutral/negative studies, suggesting no strong small-study or publication-related bias (**Figure 6B**). These findings together indicate a consistent association between anti-Ribo P Ab and NPSLE that is not explained by assay methodology, not driven by any single study, and not confined to psychosis.

**Table 10:** Studies included in the anti-Ribo P meta-analysis.

Study	*N* NPSLE (total)	Central—diffuse, *n*	Central—focal, *n*	Peripheral, *n*	Notes
Abdel-Nasser/2008 (75)	26	37 (mood disorder 19, anxiety 3, ACS 2, psychosis 1, CI 12)	20 (CVD 1, seizure disorder 4, headache 15)	0	Counts are events; some patients have multiple manifestations
Briani/2009 (76)	85	6 (psychiatric disorders 6)	58 (headache 35, CVD 11, seizure disorder 10, myelopathy 2)	30 (polyneuropathy 17, mononeuropathy 13 including multiplex 2)	Counts are events; some patients have multiple manifestations
Hanly/2008 (78)	20	12 events (ACS 5, psychosis 3, CI 1, mood disorder 3)	13 events (CVD 6, myelopathy 1, seizure disorder 4, aseptic meningitis 2)	7 events (cranial neuropathy 2, mononeuropathy 4, polyneuropathy 1)	20 patients with central NP events attributed to SLE (model A); numbers are NP events, not unique patients
Hanly/2022 (20)	54	47 (CI)	0	0	Focus on CI
Huang/2020 (48)	86	83 (mood disorders 10, CI 6, and other ACR diffuse syndromes such as ACS, psychosis and anxiety mentioned but individual counts not provided)	23 (Headache 26, seizure disorders 21, myelopathy 7)	NR (PNS involvement not separately reported)	Events. Paper does not provide a clear breakdown of each NP manifestation
Taha/2022 (79)	30	30 (ACS 16, anxiety disorder 14)	26 headaches	0	Prominent headache and diffuse psychiatric/anxiety manifestations; categories may overlap
Isshi-Hirohata/1996 (81)	48	28 (psychosis 28)	19 (seizures 11, involuntary movements 1, focal lesions 7)	0	Focus on lupus psychosis
Jönsen/2003 (36)	68	8 (Psychosis 3, organic brain syndrome 1, CI 3, depression 2)	22 (Seizures 4, focal deficits 7, myelitis 2, headache 9)	5 (Paresthesia)	Counts are manifestations and can exceed NPSLE total
Seth/2020 (39)	167	74 (ACS 11, psychosis 31, mood disorder 28, CI 2, anxiety 2)	122 (headache 16, CVD 18, seizure disorder 69, demyelinating syndrome 10, aseptic meningitis 9	25 (mononeuropathy 17, peripheral neuropathy 6, cranial neuropathy 2)	Counts are manifestations and can exceed NPSLE total because many patients had multiple NP syndromes
Arnett/1996 (80)	30	32 (psychosis 17, mood disorder 15)	NR	NR	Case–control comparison restricted to diffuse psychiatric NPSLE (psychosis and major depression); focal CNS and PNS phenotypes not detailed
Schneebaum/1991 (77)	82	37 (severe depression 8, psychosis 29)	45 (other CNS syndromes including seizure, organic brain syndrome, coma and stroke)	0 (none reported)	each patient classified into a single category

**Figure 6:**
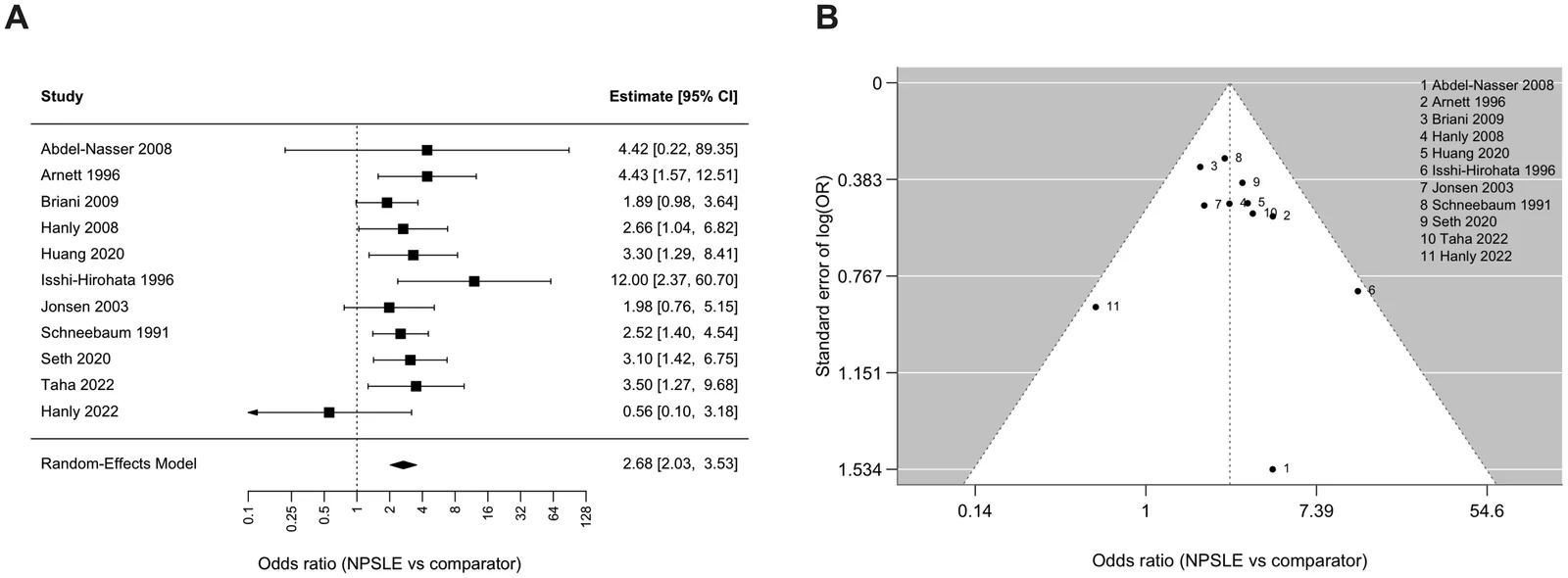
**(A)** Forest plot of serum anti-Ribo P antibodies in NPSLE versus SLE without NPSLE. Effect sizes are expressed as odds ratios (from log odds ratios, plotted on a logarithmic scale), with squares indicating study-specific estimates (size proportional to precision), horizontal lines indicating the 95% confidence intervals, and the diamond indicating the random-effects pooled estimate. **(B)** Funnel plot of the same studies, showing log(OR) on the x-axis and its standard error on the y-axis; each dot represents one study (listed on the right), the vertical dashed line marks the pooled effect, and the dashed diagonal lines indicate the expected 95% region in the absence of small-study or publication bias.

Four additional studies were not suitable for quantitative synthesis either because they lacked sufficient raw data (2 × 2 tables or precise odds ratios with confidence intervals) or because they reported time-to-event hazard ratios from Cox models rather than cross-sectional NPSLE vs. non-NPSLE contrasts. Bankole et al. (82) retrospectively studied 263 SLE patients and found that NPSLE was associated with antiphospholipid Ab (aPL) and showed a trend toward an association with low C4, whereas other SLE-related autoAbs, including anti-Ribo P, did not differ between NPSLE and non-NPSLE patients. In a prospective Italian cohort of 149 SLE outpatients, Gerli et al. (83) reported a 12% prevalence of serum anti-Ribo P; these Ab clustered with younger disease onset, more active disease, and aCL positivity but showed no association with any ACR-defined neuropsychiatric syndrome or with CI. In the SLICC inception cohort, Hanly et al. (2011) (63) followed 1,047 newly diagnosed SLE patients and found that baseline anti-Ribo P Ab (present in ~9%) predicted subsequent lupus psychosis attributed to SLE (14 events; HR 3.92, 95% CI 1.23 to 12.50), while other neuropsychiatric outcomes were not clearly linked to anti-Ribo P Ab. A later SLICC analysis focused specifically on psychosis (Hanly 2019; 1,826 patients, 28 psychosis cases) (64) confirmed a univariable association with anti-Ribo P, but this attenuated and lost statistical significance after adjustment for sex, age, and race/ethnicity.

##### CSF anti-Ribo P

Isshi and Hirohata (81) found that serum but not CSF anti-Ribo P Ab was elevated in lupus psychosis. Tjensvoll et al. (19) reported extremely low CSF anti-Ribo P levels in both SLE and primary Sjögren’s syndrome, with no disease specificity. Similarly, Jönsen et al. (36) only detected positive CSF anti-Ribo P in five of 39 patients equally distributed between NPSLE and non-NPSLE. Overall, the evidence suggests that, in contrast to serum, CSF anti-Ribo P is generally low and not a reliable biomarker.

#### Antiphospholipid Ab

Most studies that measured antiphospholipid (aPL) Ab (IgM/IgG aCL, IgM/IgG anti-β2GPI, and LA) reported outcomes in formats not suitable for pooling, which resulted in fewer than five compatible datasets for serum aCL IgM, anti-β2GPI IgG and IgM, LA, and CSF aPL Ab. Therefore, they were synthesized narratively. Studies restricted to particular NPSLE manifestations (e.g., seizures, CVD, headache, and CI) or within-NPSLE contrasts (e.g., stroke vs. non-stroke NPSLE) are also summarized narratively.

The overall study designs, NPSLE manifestations, and aPL specificities are summarized in **Table 11**.

**Table 11:** Overview of studies contributing to the serum antiphospholipid antibody data.

Study (first author, year)	NP comparison (case vs. control)	*N* case	*N* control	NPSLE manifestations contributing (cases)	Any aPL (composite)	LA	aCL IgG	aCL IgM	β2GPI IgG	β2GPI IgM
Abdul-Sattar 2013 (87)	NPSLE vs. non-NPSLE SLE	36	48	NPSLE (36); CVD 6, seizure 3, headache 10, mood 16, anxiety 7, psychosis 4, CI 1, polyneuropathy 4, mononeuropathy 2 aPL used to predict *severity* within NPSLE.	–	+	+	+	–	–
Afeltra/2003 (40)	NPSLE vs. non-NPSLE SLE	44	17	NPSLE (44); myelopathy 2, CVD 15, seizure 7, headache 13, anxiety 4, CI 32, mood 17, autonomic 2, polyneuropathy 8, cranial neuropathy 3	–	+	+	+	+	+
Alarcón-Segovia/1997 (86)	NPSLE vs. SLE without NP	238	429	NPSLE (migraine 93, seizures 59, CVD 57, peripheral neuropathy 53, psychosis 51, Sneddon’s syndrome (CVD + livedo) 26, transverse myelitis 6, chorea 4); phenotype breakdown NR. aPL measured as anticardiolipin IgG and/or IgM (ELISA). Composite aPL (aCL IgG and/or IgM, any titer but not separated)	–	–	+	+	–	–
Appenzeller/2008 (66)	Psychosis during follow-up vs. no psychosis (prognostic, time-to-event)	89	431	SLE patients with psychosis vs. SLE without psychosis; primary analysis is *prognostic* (good vs. poor outcome) Acute psychosis (89 total): 59 primary CNS-SLE psychosis, 28 corticosteroid-induced, 2 psychosis unrelated to SLE/drugs aPL (“moderate–high titer”) enter logistic models, but no table stratifies psychosis vs. no psychosis by aPL status	–	–	–	–	–	–
Bankole/2021 (82)	Any NPSLE vs. non-NPSLE SLE	76	160	Any NPSLE (76 with aPL data); phenotype NR. Only composite “any aPL”	+	–	–	–	–	–
Denburg/1997 (90)	CI vs. normal cognition (SLE)	39	79	CI (39) defined as ≥3 cognitive domain z-scores < –1.64 in SLE cohort.	–	+	–	–	–	–
Hanly/2008 (78)	CVD vs. SLE with no NP events	14	279	CVD attributed to SLE (14)	+	+	–	–	–	–
Hanly/2011 (63)	Incident NP event(s) during follow-up vs. no NP events (SLE inception cohort; time-to-event design)	495	552	Inception cohort with Cox models for time to first NP event. They report prevalence of each antibody at enrollment and hazard ratios for NP outcomes (CVD and psychosis) Any NP event (495 patients; 917 NP events total across all 19 ACR NP syndromes; NP events attributed to SLE in 141 [model A] and 259 [model B])	–	–	–	–	–	–
Hanly/2019 (64)	Psychosis vs. no psychosis (SLE inception cohort; time-to-event design)	28	1798	Psychosis (28 patients; 31 episodes, mostly SLE-attributed under model B) Prospective Cox models. Antibodies (LA, aCL, anti-β2GPI) are analyzed as predictors in Cox models	–	–	–	–	–	–
Hanly/2022 (20)	CI vs. normal cognition (SLE)	47	54	CI (47)	+	+	+	–	+	–
Hawro/2015 (91)	Specific NP phenotypes vs. SLE without those phenotypes	42	47	NPSLE overall 42; CVD 10, nonspecific intractable headache 10, seizure 8, plus other NP manifestations (headache types, polyneuropathies, etc.)	–	+	–	–	+	+
Herranz/1994 (85)	Epilepsy vs. no epilepsy (SLE)	21	197	Epilepsy/seizure disorder (21)	+	+	+	+	–	–
Jönsen/2003 (36)	Any NPSLE vs. non-NPSLE SLE	68	70	Any NPSLE (68); mix of focal, diffuse and peripheral NP manifestations	–	–	+	–	–	–
Mikdashi/2005 (65)	Seizures vs. no seizures (SLE)	28	167	Seizure disorder (28)	–	+	+	–	–	–
Mok/2001 (84)	Any NPSLE vs. non-NPSLE SLE	96	422	Any NPSLE (96); CVD 26, seizure 37, myelopathy 10, headache 5, movement 3, demyelinating 2, aseptic meningitis 1, ACS 18, psychosis 14, mood 8, anxiety 2, cranial neuropathy 4, mononeuritis/mononeuropathy 2, polyneuropathy 1	–	+	+	–	–	–
Monahan/2022 (28)	Major NPSLE vs. non-NPSLE SLE	104	245	104 (detailed breakdown NR)	–	–	+	–	–	–
Sanna/2003 (89)	NP phenotypes vs. SLE without each phenotype	varies	varies	Within larger NP cohort (185): CVD 47, headaches 78, seizure 27, mood 54, ci 35, psychosis 25, anxiety 24, ACS 13, myelopathy 4, demyelinating 3, chorea 4, polyneuropathy 9, mononeuritis 6, mg 5, cranial neuropathy 5, GBS 2	+	+	+	–	–	–
Seth/2020 (39)	Demyelinating/stroke subsets within NPSLE	82 demyelinating/138 non-demyelinating; 95 stroke/383 non-stroke	**-**	Demyelinating NPSLE 82 vs. other NPSLE 138; stroke 95 vs. non-stroke 383 within broader NPSLE sample Only NPSLE patients; comparisons are within NPSLE (stroke vs. non-stroke, demyelinating vs. other NPSLE)	–	+	–	–	+	–
Taha/2022 (79)	NPSLE vs. non-NPSLE	30	60	Any NPSLE (30); phenotype breakdown NR	–	+	+	–	–	–
Toubi/1995 (92)	CNS-SLE vs. SLE without CNS disease	96	100	CNS-SLE (96); CVD 56, seizure 24, myelopathy 3, movement 1, psychiatric disorders 12, polyneuropathy 1	+	–	–	–	–	–
Van der Meulen/2017 (27)	NPSLE vs. non-NPSLE	69	203	Any NPSLE (69); CVD 41, seizure 10, headache 10, demyelinating 1, movement 2, myelopathy 2, CI 30, mood 13, psychosis 6, ACS 3, anxiety 4, polyneuropathy 9, autonomic 1, mononeuropathy 1, cranial neuropathy 1	–	–	+	–	–	–
Velarde-Mejía/2024 (88)	SLE with CI vs. no CI	90	45	135 SLE; cognitive status by NEUROPSI (90 impaired, 45 normal) aCL IgG/IgM, anti-β2GPI IgM and LA evaluated only in linear models; no dichotomous counts by cognition status	–	–	–	–	–	–

This table summarizes all retrieved studies that examined antiphospholipid Ab in relation to neuropsychiatric involvement in SLE. For each study, we indicate the NP comparison, the number of cases and controls (if pertinent), and the specific NPSLE manifestations included in the case group. The right-hand columns show which Ab were analyzed: “Any aPL (composite)” denotes a combined definition (aCL and/or anti-β2GPI and/or LA), whereas + entries under LA, aCL IgG/IgM, and β2GPI IgG/IgM indicate that a separate 2 × 2 comparison was available for that specificity. Studies with “–” for all Ab columns assessed aPL only in regression or time-to-event models and did not provide data stratified by both NP and antibody status. In bold are papers included in the meta-analysis (aCL IgG).

CVD, cerebrovascular disease; CI, cognitive impairment; ACS, acute confusional state; CNS, central nervous system SLE; “Any”, any NPSLE vs. non-NPSLE SLE.

##### Serum anticardiolipin

Six studies (27, 28, 36, 40, 79, 84) comparing serum IgG aCL Ab in NPSLE with non-NPSLE SLE met our inclusion criteria for pooling. Although assay platforms and positivity thresholds varied across studies, all dichotomous odds ratios were derived using the original study-specific definitions of antibody positivity rather than applying a uniform threshold across studies (**Supplementary Table 5**). In the random effects meta-analysis (**Figure 7**), IgG aCL positivity was associated with higher odds of NPSLE, with a pooled log odds ratio of 0.86 (SE 0.21, *z* = 4.04, *p* < 0.0001), corresponding to an OR of 2.36 (95% CI 1.56 to 3.59). Heterogeneity was low (*τ*² = 0.06, *I*² ≈ 20%, Q[5] = 8.93, *p =* 0.11), and the 95% prediction interval on the OR scale was 1.27 to 4.40, suggesting that future studies in similar settings would also be expected to show a positive association, with effects typically in the two- to fourfold range. The leave-one-out analyses yielded log OR estimates between 0.73 and 1.02 (OR ≈ 2.07 to 2.77), indicating that the association is not driven by any single study.

**Figure 7:**
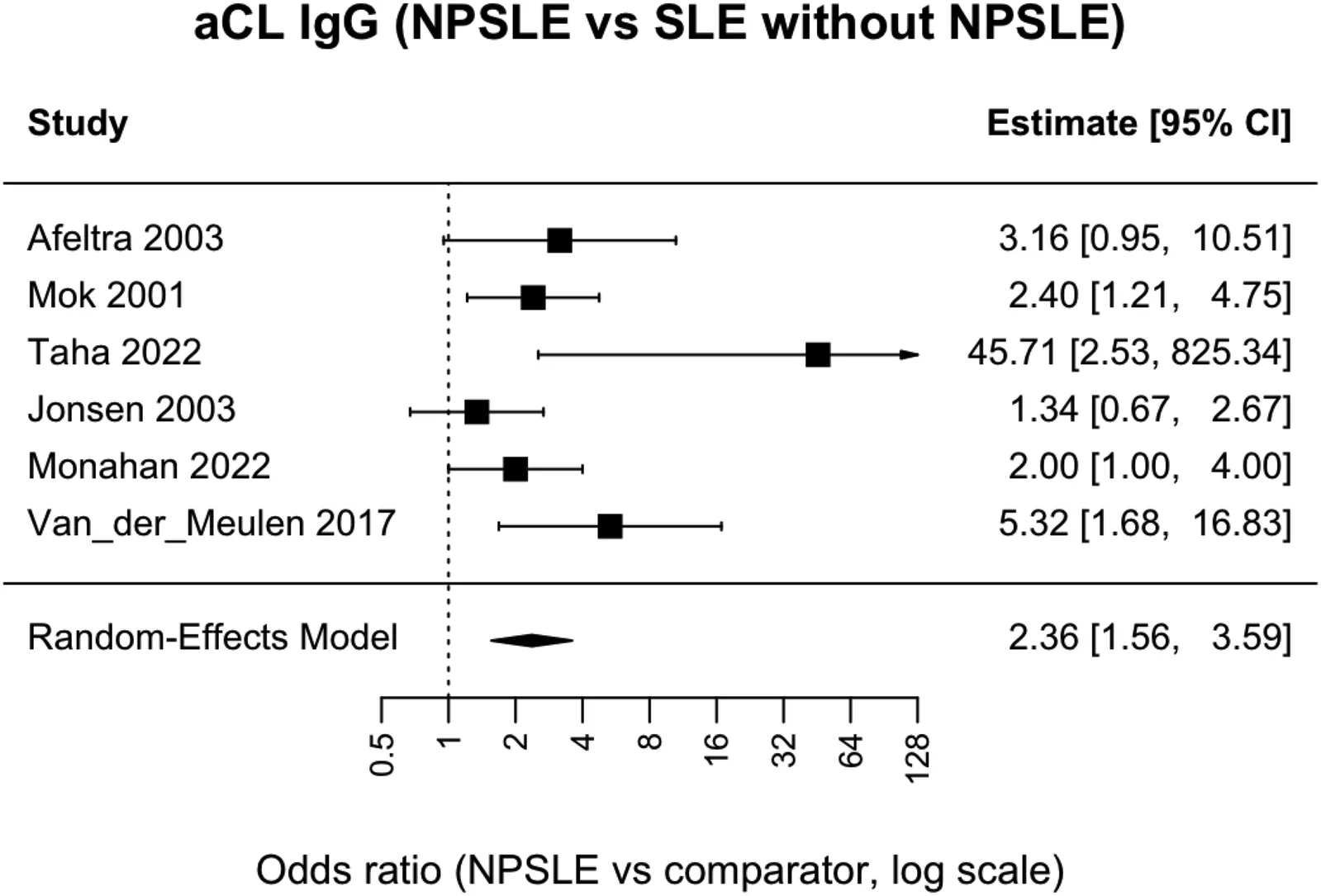
Forest plot of IgG anticardiolipin (aCL) antibodies in NPSLE versus SLE without NPSLE. Effect sizes are expressed as odds ratios (from log odds ratios, plotted on a logarithmic scale), with squares showing study-specific estimates (size proportional to precision), horizontal lines showing the 95% confidence intervals, and the diamond showing the random-effects pooled estimate.

Regarding studies not included in the meta-analysis, Herranz et al. (85) and Mikdashi et al. (65) observed that moderate-high IgG aCL predicted epilepsy/seizures. Similarly, Appenzeller et al. (66). reported that moderate-to-high titer aPL (aCL and/or LA) independently increased the risk of developing psychosis during follow-up. However, in a large inception cohort, Alarcón-Segovia et al. (86) did not observe cross-sectional associations between aCL (IgM and IgG combined) and neurological manifestations overall, highlighting the dependence of the aCL associations on case definition and titers. Abdul-Sattar et al. (87) studied 84 SLE patients, 36 of whom had NPSLE, and mainly examined predictors of severe versus non-severe NP disease within the NPSLE group rather than NPSLE vs. non-NPSLE. They found that IgG aCL and LA were more frequent in patients with severe NPSLE, and in multivariable models IgG aCL (along with nephritis flare and cutaneous vasculitis) remained independently associated with severe NP manifestations, suggesting that aPL may contribute to NP severity rather than serving as a simple cross-sectional marker of NPSLE presence.

Reports of serum IgM aCL Abs are fewer in number and significant results. Velarde-Mejía et al. (88) reported that IgM aCL was associated with lower cognitive test scores in univariable models, but this attenuated after adjustment for demographics, disease activity, and treatment. IgM aCL also showed no association with seizures in the study of Herranz et al. (85).

##### Serum lupus anticoagulant

Under our predefined criteria, only three studies provided extractable data for serum LA (40, 79, 84). We therefore present LA findings descriptively.

Afeltra et al. (40) found that LA positivity was numerically higher in NPSLE than in non-NPSLE, although the difference was modest and not clearly discriminatory. In the study of Mok et al. (84), NPSLE overall was significantly more frequent in LA-positive patients (*p =* 0.01), and CVD, in particular, was associated with LA (OR 3.3, 95% CI 1.4 to 8.0) as well as with strongly positive IgG aCL (OR 3.1, 95% CI 1.1 to 8.2). Taha et al. (79) reported LA in eight of 30 NPSLE patients and in none of 60 non-NPSLE SLE controls, giving a very large odds ratio of 45.7 (95% CI 2.5 to 825.3) after continuity correction; the wide confidence interval reflects sparse data rather than a precise estimate of effect size. Similarly, Bankole et al. (86) found that NPSLE patients were more often positive for a composite “any aPL” definition that included LA (reported OR ≈ 4), and Abdul-Sattar et al. (87) observed that within the NPSLE group LA (along with IgG aCL) tended to cluster in patients with more severe NP manifestations.

Other studies examining LA most consistently linked it to focal, thrombotic CNS manifestations rather than to NPSLE overall. In a mixed NPSLE cohort, LA positivity was more frequently seen in patients with SLE-attributed CVD and seizures and was also associated with demyelinating events and white-matter MRI lesions, suggesting both macro- and microthrombotic effects (Sanna et al., 2003) (89). In the SLICC inception cohort, baseline LA or composite aPL predicted subsequent stroke or sinus thrombosis, reinforcing its role as a marker of thrombotic CNS risk (Hanly et al., 2008; Hanly et al., 2011) (63, 78). Seizure-focused work similarly identified LA as an independent predictor of seizure occurrence in SLE (Mikdashi et al., 2005) (65). In the cognitive field, Denburg et al. (90) reported a roughly two- to threefold higher odds of clinically important CI among LA-positive patients, including within a “never-NP” subgroup, consistent with subclinical small-vessel disease. By contrast, associations between LA and diffuse psychiatric syndromes such as psychosis or mood disorder were weak or unstable after multivariable adjustment, including a psychosis cohort and the SLICC cohort analyses (Appenzeller et al., 2008; Hanly et al., 2019) (64, 66). Taken together, these non-pooled studies reinforce the view that LA is primarily a risk marker for thrombotic CNS manifestations (both micro- and macrovascular), with much weaker and less consistent evidence for a direct role in diffuse psychiatric NPSLE.

##### Serum anti-β2-glycoprotein I

In contrast to aCL and LA, serum anti-β2-glycoprotein I (β2GPI) antibodies (IgG and IgM) show weaker and less consistent associations with NPSLE overall. Specifically, Alarcón-Segovia et al. (86) and Afeltra et al. (40) did not find significant differences in β2GPI positivity between NPSLE and non-NPSLE.

Phenotype-specific analyses provide more nuance. Seth et al. (39) reported enrichment of IgG β2GPI in NPSLE patients with stroke compared with other NPSLE phenotypes, and Hawro et al. (91) found IgM β2GPI associated with stroke and intractable headache, as well as IgG β2GPI with seizure and grand-mal seizure subsets, although all associations were based on small case numbers. Cognitive-focused studies (20, 88) observed that IgG or IgM β2GPI correlated with worse cognitive performance in univariable analyses, yet these associations weakened and lost significance after adjustment for demographics, disease activity, and therapy. Similarly, time-to-event data from the SLICC cohort did not show strong, independent hazard signals for β2GPI once LA and other covariates were included (63).

##### Composite “any serum aPL positive”

For the composite “any aPL positive” exposure (aCL and/or anti-β2GPI and/or LA), Bankole et al. (82) reported that NPSLE patients were substantially more likely to be composite aPL positive than SLE controls (OR ≈ 4.2), and Toubi et al. (92) found a similarly increased prevalence of aPL in CNS SLE versus SLE without CNS involvement (OR ≈ 5.0). The remaining studies using a composite aPL exposure were phenotype specific. In the SLICC inception cohort, Hanly et al. (2008) (78) showed that composite aPL positivity was clearly more frequent among SLE patients with CVD at or near diagnosis than among SLE patients with no NP events and that this pattern was consistent with an increased subsequent risk of stroke or sinus thrombosis. Sanna et al. (89) reported that composite aPL positivity was more common in SLE patients with CVD and seizures than in SLE patients without those manifestations, with smaller and less consistent differences for chronic headache. In a cognition-focused study, Hanly et al. (2022) (20) found that composite aPL positivity tended to be higher in SLE patients with objectively defined CI than in those with normal cognition, although this difference was modest and not clearly independent of age, education, and vascular factors.

##### CSF antiphospholipid antibodies

Finally, CSF aPL were rarely positive. Pereira et al. (36, 44) and Jönsen et al. (36) reported very low or absent CSF aCL and LA positivity in NPSLE compared with SLE controls, indicating that intrathecal aPL testing adds little diagnostic information beyond serum assays.

## Discussion

In this systematic review, we synthesized evidence on serum, plasma, and CSF biomarkers in adult patients with NPSLE from 93 studies spanning more than four decades. Despite the many analytes that have been studied over those years, only a minority of analyte–biofluid combinations met our predetermined threshold of five independent studies with extractable data for meta-analysis, and most promising biomarkers have been evaluated in one or two small heterogeneous studies.

Notably, in most studies, PNS manifestations were embedded as small subgroups within populations with predominantly CNS NPSLE syndromes; thus, PNS involvement was markedly under-represented. In one study (51), serum S100B emerged in secondary analyses as a *fair* discriminator of peripheral polyneuropathy (AUC ≈ 0.71), but PNS disease was not the primary focus of this work. Consequently, the findings reported here apply predominantly to CNS NPSLE.

From this review, several promising serum (anti-amyloid, anti-α-internexin, anti-collagen II IgM, anti-GP2, anti-heparin sulfate, anti-MAA, anti-nucleoporin 62, anti-prothrombin, anti-UCH-L1, anti-vimentin, basic FGF, GSH, IL-9, MIP-1α, and S100A8/A9) and CSF (α-Klotho, anti-α-internexin, anti-UCH-L1, APRIL, basic FGF, GFAP, IL-1ra, IL-5, IL-7, IL-9, IL-12(p70), IL-15, MIP-1α, NfL, PAI-1, P-selectin, TGF-β, and VCAM-1) analytes emerged as candidate cross-sectional state biomarkers distinguishing NPSLE from non-NPSLE (**Table 1**). However, none of these studies met our criteria for meta-analysis, and the likelihood that some will fail replication or yield conflicting results in future cohorts is substantial. Thus, all should be considered provisional until independently validated.

Additionally, many of these signals come from single studies using large multiplex or protein-array panels [e.g., serum anti-collagen II IgM, anti-GP2, anti-heparin sulfate, anti-MAA, anti-nucleoporin 62, anti-prothrombin, and anti-vimentin in Van der Meulen et al. (2017) (27), CSF IL-1ra, IL-5, IL-7, IL-9, IL-12(p70), IL-15 in Ushigusa et al. (2016) (11), and CSF P-selectin and VCAM-1 in Lu et al. (2010) (24)], where extensive multiple testing raises a substantial risk of type I error. These analytes should therefore be regarded as hypothesis-generating only, pending replication in independent cohorts with prespecified analyses.

Also presented in **Table 1** are earlier studies of “anti-neuronal” and lymphocytotoxic Ab that relied on in-house cell-based assays using neuroblastoma lines (e.g., SK-N-SH, SK-N-MC) (13, 14, 16) or microcytotoxicity on donor lymphocytes (31), respectively. These techniques require live cells, complex handling, and lab-specific read-outs without standardized calibration or cutoffs, making them technically challenging and difficult to reproduce across centers. Moreover, broad reactivity to these cell substrates was also seen in non-NPSLE and other autoimmune or psychiatric controls (13, 16), and associations with individual NP syndromes were weak or inconsistent (14, 31). Taken together, these early anti-neuronal and lymphocytotoxic Ab assays are best viewed as exploratory evidence of broad, polyclonal autoAb responses against neuronal and lymphocyte surface antigens rather than as specific, clinically useful biomarkers for NPSLE.

Remarkably, across the abovementioned analytes, CSF findings rarely had a clear serum or plasma counterpart when paired blood and CSF-derived analytes were examined or when independent studies examining the same analyte in different biofluids were available—for instance, anti-neuronal Abs were increased in CSF, whereas paired serum samples were uninformative (13, 14). Anti-UCH-L1 Ab showed a similar dissociation, with elevated titers in CSF but not in blood in one paired study (15). The APRIL levels were also higher in CSF from NPSLE than in non-NPSLE SLE patients without systemic correlates (22). This discrepancy was particularly compelling for the analyte NfL, a marker of structural damage, where two studies showed high CSF levels (18, 19) and two different independent studies reported no difference in blood derivates (21, 20). In keeping with this compartmentalized pattern, serum NGAL (lipocalin-2) did not differ between cognitively impaired and cognitively intact SLE in the only eligible study (32), but an aptamer-based proteomic study found lipocalin-2 to be elevated in CSF from NPSLE compared with healthy and non-SLE neurological controls, albeit in two small cohorts (55). The only soluble mediators that met our criteria for meta-analysis (IL-6, IL-8, and MCP-1/CCL2) all tended to be likewise higher in the CSF of NPSLE patients, with pooled Hedges’ *g* of 0.81 and 0.66 for IL-6 and MCP-1, respectively, and a smaller, highly heterogeneous effect for IL-8. Although these findings support intrathecal inflammation as a feature of NPSLE, clinically applicable CSF biomarker cutoff values cannot currently be recommended. The available studies used heterogeneous assay platforms, commercial kits, laboratory protocols, and reporting methods, and only a limited number included appropriate neurological disease-control groups. Standardized assay methodologies and prospective validation against relevant inflammatory neurological disorders will be required before clinically useful diagnostic thresholds can be established.

Importantly, among the studies included in the CSF IL-6 and MCP-1 meta-analyses, disease activity was reported inconsistently, none matched participants on disease activity, and only one study adjusted its analyses for disease activity. Although several studies reported higher SLEDAI or SELENA-SLEDAI scores in patients with NPSLE, these indices include neuropsychiatric manifestations and therefore do not isolate systemic disease activity. Consequently, the pooled associations cannot distinguish whether elevated CSF IL-6 and MCP-1 reflect NPSLE-specific CNS inflammation, greater systemic SLE disease activity, or both.

By contrast, markers of innate immunity provided a more nuanced picture. Higher serum S100A8/A9 (calprotectin) was associated with CI in our cohort (32) and with NPSLE in an independent study where CSF S100A8/A9 did not distinguish groups (33). The serum S100B results were heterogeneous: some studies showed higher levels and fair discrimination in acute CNS NPSLE and peripheral neuropathy (49, 51), whereas others, including our cognition-focused cohort, showed only trends or null results (32); CSF S100B was uninformative (19, 50). These diverse data highlight questions about specific biologic mechanisms associated with different clinical manifestations and the role of blood–brain barrier leakage in NPSLE. Study design is another important consideration as cross-sectional analyses are limited by states of disease activity, which do not inform us about causality and whether analytes may be present in serum first as a pre-requisite to accessing the CNS.

It is worth noting that several CSF markers were not specific for NPSLE—for instance, in the study of Fragoso-Loyo et al. (37), SLE patients with infectious meningitis/encephalitis showed IL-6, IL-8, IL-17, MIG, IP-10, MCP-1, and RANTES levels similar to or higher than those in NPSLE, producing an infection > NPSLE > healthy/non-inflammatory CNS gradient. Ichinose et al. (56) and Ushigusa et al. (11) likewise found a substantial overlap between NPSLE and MS/NMO, with only a subset of cytokines (e.g., IL-2, IL-5, IL-7, IL-9, IL-10, IL-12p70, IL-13, IL-15, IL-17, IFN-γ, GM-CSF, TNF-α, FGF-b, and eotaxin) (56) and low CSF α-Klotho clearly discriminating groups (11). Overall, these patterns suggest that most CSF analytes capture generic CNS inflammatory activity rather than a disease-specific NPSLE signature. Moreover, as compelling as the CSF findings are, the invasiveness and limited feasibility of lumbar puncture in routine practice makes the identification of blood-based surrogates of CNS involvement in SLE paramount. Developing such markers will almost certainly require multicenter collaboration and, somewhat paradoxically, carefully designed early studies with paired CSF–serum samples to define reliable peripheral correlates of intrathecal pathology.

Among serum autoAb, anti-Ribo P had the strongest quantitative support. Our random-effects meta-analysis of 11 studies showed approximately 2.7-fold higher odds of NPSLE in anti-Ribo P-positive versus anti-Ribo P-negative SLE (pooled OR 2.68, 95% CI 2.03–3.53), with minimal heterogeneity. These findings are consistent with earlier meta-analyses, which reported similar effect sizes for diffuse NPSLE syndromes (93, 94). However, the prevalence of anti-Ribo P in SLE varies widely across populations and is strongly influenced by assay methodology and demographic factors (93), highlighting that, despite the absence of statistically detectable heterogeneity in our meta-analysis, biological and methodological differences across studies should be considered when interpreting these findings. Similarly, many patients with NPSLE are anti-Ribo P Ab-negative, limiting its value as a stand-alone diagnostic marker.

On the other hand, our meta-analysis of five studies examining anti-NR2/NMDAR Ab (255 NPSLE and 252 non-NPSLE SLE) showed that positivity was associated with NPSLE (pooled OR 2.09, 95% CI 1.07–4.06), but with moderate heterogeneity and a wide prediction interval. Moreover, the leave-one-out analyses confirmed the direction of effect but showed that statistical significance depended on individual studies. This mirrors an earlier meta-analysis of anti-NR2 which found modest, heterogeneous associations with overall NPSLE and where peripheral SLE disease activity and co-existing anti-dsDNA Ab were found to be significant moderators of this heterogeneity (95). Although mechanistically anti-NR2/NMDAR Ab could be more relevant to specific NPSLE manifestations rather than NPSLE overall, the studies that examined anti-NR2 Ab in SLE CI included here generally reported null findings, consistent with some previous systematic reviews (95, 96).

For IgG aCL, six eligible studies showed roughly 2.4-fold increased odds of NPSLE (pooled OR 2.36, 95% CI 1.56–3.59; *I*² ≈ 20%). Beyond this overall association, phenotype-specific and longitudinal studies narratively synthesized here consistently linked LA and high-titer aCL or anti-β_2_GPI with cerebrovascular events and seizures, whereas associations with diffuse psychiatric syndromes were weak or disappeared after multivariable adjustment. This pattern aligns with previous reviews (7, 97). Taken together, aPL Ab are best regarded as risk markers of focal thrombotic CNS involvement within SLE, not diagnostic markers for NPSLE as a whole.

Finally, longitudinal data on prognostic and monitoring biomarkers in NPSLE were scarce and heterogeneous. Across CSF studies, cytokines and adhesion molecules behaved mainly as activity and treatment-response markers rather than long-term predictors: higher baseline IL-6, IL-10, IFN-γ, and TNF-α identified non-responders in one cohort (60), while IL-6, IL-8, MCP-1, IP-10, MIG, P-selectin, and VCAM-1 consistently declined after induction therapy in parallel with clinical improvement in others (24, 37). In peripheral blood, elevated baseline BAFF predicted higher 24-month risk of relapse or death in a single study (46). Anti-Ribo P and aPL were linked to early mortality, seizures, psychosis, and accrual of cognitive/cerebrovascular damage in some cohorts (61) (62, 66). However, analyses from the SLICC inception cohort did not confirm anti-Ribo P as an independent predictor of overall NP events or psychosis after adjustment for demographic and clinical factors, and in the same large cohort after adjusted models, aPL and other Abs generally failed to independently predict NP events (63, 64). Overall, current prognostic biomarker candidates seem better at reflecting contemporaneous CNS inflammation and short-term treatment response than at forecasting long-term neuropsychiatric outcomes in NPSLE. This may be due, in part, to the limited availability of long-term outcomes.

The strengths of this review include a comprehensive multi-database search, explicit inclusion and exclusion criteria, restriction to adult populations with at least 20 NPSLE patients, and strict separation of CSF and blood compartments. Additionally, we harmonized NP syndromes to the 1999 ACR framework, summarized results overall and by phenotype where possible, and required at least five independent studies per analyte–matrix combination before meta-analysis, with sensitivity analyses and prediction intervals to aid interpretation. A limitation of this review is that the study protocol was not prospectively registered (e.g., in PROSPERO), which may reduce transparency regarding prespecified review methods and analyses. However, we confirm that the review methodology, including the eligibility criteria, data extraction process, and planned analyses, was established before study selection and data synthesis. In addition, although calibration exercises demonstrated high inter-reviewer agreement, the remaining title/abstract records and full-text articles were divided between reviewers rather than screened independently in duplicate because of the large number of retrieved records. This approach may have increased the possibility of missed eligible studies. Additionally, our predefined eligibility criterion requiring at least 20 patients with NPSLE may have excluded some small exploratory studies; however, this criterion was adopted to reduce the influence of very small cohorts with potentially imprecise effect estimates and to improve the methodological consistency of the evidence synthesis. Finally, methodological quality assessment was performed by a single reviewer, which may have introduced some subjectivity into the risk-of-bias assessment despite the use of a standardized appraisal tool.

Key limitations arise from the underlying retrieved eligible studies: most studies were cross-sectional and often retrospective, NPSLE definitions and attribution rules varied substantially across centers and eras, sample sizes were frequently small, particularly for CSF and phenotype-specific analyses, multiplex panels often lacked rigorous correction for multiple testing, and assays and cutoffs differed even for the same analyte. Furthermore, exploration of potential sources of heterogeneity was limited because clinically relevant study-level variables, including patient age, disease activity, and disease duration, were inconsistently reported across studies and were therefore unsuitable for meta-regression. An additional limitation is that phenotype-specific meta-analyses could not be performed because most primary studies included heterogeneous NPSLE populations and did not report biomarker results separately for diffuse, focal, and peripheral manifestations. Consequently, the pooled estimates represent average effects across clinically diverse NPSLE phenotypes, and biomarker performance may differ according to specific neuropsychiatric manifestations. Future studies reporting phenotype-specific data using standardized NPSLE classifications are needed to enable clinically informative subgroup meta-analyses. Similarly, for many promising markers (for example, CSF NfL, GFAP, a-Klotho, lipocalin-2, and anti-UCH-L1 Ab), only one or two eligible studies were available; thus, conclusions must remain cautious. In addition, although the available evidence through March 2024 did not demonstrate consistent differences in circulating NfL between patients with and without NPSLE, this literature is rapidly evolving. Consequently, our conclusions regarding blood-based NfL should be considered provisional and may require revision as additional studies become available.

Overall, four broad conclusions emerged. First, CSF markers show the clearest and most consistent differences between NPSLE and non-NPSLE. Second, serum autoAbs traditionally linked to NPSLE (anti-Ribo P, anti-NR2, and aPL) provide only modest diagnostic or prognostic information. Third, the evidence base is fragmented, with small samples, heterogeneous assays and cutoffs, and variable NPSLE definitions. Fourth, almost all data report on CNS NPSLE; thus, biomarkers specific for PNS involvement remain largely unexplored. These points underline the desperate need for harmonized definitions, sampling and assays, and multicenter cohorts with longitudinal CSF–blood pairing to identify reliable blood surrogates of CNS pathology. Integration of imaging and rigorous statistical practice (including correction for multiple testing and independent replication) will also be essential. Within that framework, the biomarkers highlighted here remain promising candidates, but substantial validation work is required before they can be incorporated into routine clinical practice.

## Data Availability

The original contributions presented in the study are included in the article/**Supplementary Material**. Further inquiries can be directed to the corresponding author.
