# Supplementary material for: The neuropsychiatric systemic lupus erythematosus biomarker landscape: what blood and cerebrospinal fluid analytes have been studied and how they performed? A systematic review and meta-analysis
Source: Front Immunol. 2026 Oct 1;17:1903757. doi: 10.3389/fimmu.2026.1903757 (PMC13668822; doi:10.3389/fimmu.2026.1903757)
Supplement: Supplementary file 1 [file SupplementaryFile1.docx]

**Search strategy documentation**

# **Searches run on June 2, 2022**

- Ovid MEDLINE(R) ALL 1946 to June 01, 2022
- Ovid Embase Classic+Embase 1947 to 2022 June 01
- Ovid EBM Reviews - Cochrane Central Register of Controlled Trials April 2022

# **Searches run on March 14, 2024**

- Ovid MEDLINE(R) ALL 1946 to March 13, 2024
- Ovid Embase Classic+Embase 1947 to 2024 March 13
- Ovid EBM Reviews - Cochrane Central Register of Controlled Trials February 2024

**Ovid MEDLINE(R) ALL 1946 to June 01, 2022**

Search Strategy:

| **#** | **Searches** | **Results** |
| --- | --- | --- |
| 1 | Lupus Erythematosus, Systemic/ | 58997 |
| 2 | (systemic adj3 lupus).mp. | 76733 |
| 3 | sle.tw,kf. | 37915 |
| 4 | or/1-3 | 80158 |
| 5 | neuro*.mp. | 2381746 |
| 6 | 4 and 5 | 5367 |
| 7 | Neurolupus.mp. | 64 |
| 8 | CNS lupus.mp. | 157 |
| 9 | (central nervous system adj6 systemic lupus).mp. | 321 |
| 10 | npsle.mp. | 596 |
| 11 | or/6-10 | 5596 |
| 12 | Biological Products/ | 31570 |
| 13 | exp Cytokines/ | 763661 |
| 14 | exp Antibodies/ | 884479 |
| 15 | biologics.mp. | 14373 |
| 16 | (biologic* adj3 agent*).mp. | 19613 |
| 17 | (biologic* adj3 therap*).mp. | 18947 |
| 18 | biologicals.mp. | 3200 |
| 19 | cytokine*.mp. | 469434 |
| 20 | chemokine*.mp. | 106081 |
| 21 | interferon*.mp. | 218597 |
| 22 | interleukin*.mp. | 391815 |
| 23 | lymphokine*.mp. | 26408 |
| 24 | monokine*.mp. | 3139 |
| 25 | antibod*.mp. | 1241321 |
| 26 | autoantibod*.mp. | 98798 |
| 27 | isoantibod*.mp. | 14093 |
| 28 | (inflamm* factor* adj4 mediator*).mp. | 50 |
| 29 | metalloproteinas*.mp. | 84365 |
| 30 | belimumab.mp. | 871 |
| 31 | benlysta.mp. | 41 |
| 32 | lymphostat B.mp. | 8 |
| 33 | rituximab.mp. | 28313 |
| 34 | ritemvia.mp. | 1 |
| 35 | ritumax.mp. | 0 |
| 36 | rituxan.mp. | 329 |
| 37 | rituxin.mp. | 1 |
| 38 | rituzena.mp. | 0 |
| 39 | rixathon.mp. | 8 |
| 40 | riximyo.mp. | 3 |
| 41 | ruxience.mp. | 6 |
| 42 | truxima.mp. | 20 |
| 43 | tuxella.mp. | 0 |
| 44 | anifrolumab.mp. | 99 |
| 45 | tabalumab.mp. | 56 |
| 46 | epratuzumab.mp. | 249 |
| 47 | lymphocide.mp. | 1 |
| 48 | atacicept.mp. | 108 |
| 49 | blisibimod.mp. | 20 |
| 50 | tofacitinib.mp. | 2305 |
| 51 | ocrelizumab.mp. | 693 |
| 52 | abatacept.mp. | 4192 |
| 53 | edratide.mp. | 17 |
| 54 | lupuzor.mp. | 12 |
| 55 | forigerimod.mp. | 1 |
| 56 | sirukumab.mp. | 59 |
| 57 | plivensia.mp. | 0 |
| 58 | tocilizumab.mp. | 5703 |
| 59 | actemra.mp. | 64 |
| 60 | atlizumab.mp. | 19 |
| 61 | lusinex.mp. | 1 |
| 62 | roactemra.mp. | 23 |
| 63 | sifalimumab.mp. | 41 |
| 64 | rontalizumab.mp. | 15 |
| 65 | ustekinumab.mp. | 2710 |
| 66 | baricitinib.mp. | 867 |
| 67 | PF-04236921.mp. | 4 |
| 68 | dapirolizumab.mp. | 8 |
| 69 | bortezomib.mp. | 10317 |
| 70 | IL-2.mp. | 54986 |
| 71 | Interleukin-2.mp. | 63227 |
| 72 | or/12-71 | 2451832 |
| 73 | 11 and 72 | 2524 |
| 74 | exp animals/ not (exp animals/ and exp humans/) | 5013096 |
| 75 | 73 not 74 | 2396 |

**Embase Classic+Embase 1947 to 2022 June 01**

Search Strategy:

| **#** | **Searches** | **Results** |
| --- | --- | --- |
| 1 | systemic lupus erythematosus/ | 110483 |
| 2 | (systemic adj3 lupus).mp. | 123749 |
| 3 | sle.tw,kf. | 65397 |
| 4 | or/1-3 | 132860 |
| 5 | neuro*.mp. | 3452084 |
| 6 | 4 and 5 | 12975 |
| 7 | Neurolupus.mp. | 153 |
| 8 | CNS lupus.mp. | 273 |
| 9 | (central nervous system adj6 systemic lupus).mp. | 419 |
| 10 | npsle.mp. | 1118 |
| 11 | or/6-10 | 13323 |
| 12 | exp biological product/ | 862122 |
| 13 | exp cytokine/ | 1747940 |
| 14 | exp antibody/ | 1491351 |
| 15 | biologics.mp. | 30142 |
| 16 | (biologic* adj3 agent*).mp. | 28919 |
| 17 | (biologic* adj3 therap*).mp. | 40295 |
| 18 | biologicals.mp. | 7413 |
| 19 | cytokine*.mp. | 745595 |
| 20 | chemokine*.mp. | 178310 |
| 21 | interferon*.mp. | 443464 |
| 22 | interleukin*.mp. | 812098 |
| 23 | lymphokine*.mp. | 17802 |
| 24 | monokine*.mp. | 2759 |
| 25 | antibod*.mp. | 1688819 |
| 26 | autoantibod*.mp. | 117186 |
| 27 | isoantibod*.mp. | 844 |
| 28 | (inflamm* factor* adj4 mediator*).mp. | 63 |
| 29 | metalloproteinas*.mp. | 124507 |
| 30 | belimumab.mp. | 3321 |
| 31 | benlysta.mp. | 270 |
| 32 | lymphostat B.mp. | 103 |
| 33 | rituximab.mp. | 102025 |
| 34 | ritemvia.mp. | 27 |
| 35 | ritumax.mp. | 0 |
| 36 | rituxan.mp. | 3290 |
| 37 | rituxin.mp. | 34 |
| 38 | rituzena.mp. | 18 |
| 39 | rixathon.mp. | 59 |
| 40 | riximyo.mp. | 29 |
| 41 | ruxience.mp. | 22 |
| 42 | truxima.mp. | 114 |
| 43 | tuxella.mp. | 1 |
| 44 | anifrolumab.mp. | 341 |
| 45 | tabalumab.mp. | 282 |
| 46 | epratuzumab.mp. | 1480 |
| 47 | lymphocide.mp. | 77 |
| 48 | atacicept.mp. | 674 |
| 49 | blisibimod.mp. | 144 |
| 50 | tofacitinib.mp. | 7310 |
| 51 | ocrelizumab.mp. | 3121 |
| 52 | abatacept.mp. | 11363 |
| 53 | edratide.mp. | 71 |
| 54 | lupuzor.mp. | 36 |
| 55 | forigerimod.mp. | 60 |
| 56 | sirukumab.mp. | 359 |
| 57 | plivensia.mp. | 3 |
| 58 | tocilizumab.mp. | 22002 |
| 59 | actemra.mp. | 679 |
| 60 | atlizumab.mp. | 566 |
| 61 | lusinex.mp. | 1 |
| 62 | roactemra.mp. | 264 |
| 63 | sifalimumab.mp. | 257 |
| 64 | rontalizumab.mp. | 152 |
| 65 | ustekinumab.mp. | 10295 |
| 66 | baricitinib.mp. | 3095 |
| 67 | PF-04236921.mp. | 38 |
| 68 | dapirolizumab.mp. | 35 |
| 69 | bortezomib.mp. | 37436 |
| 70 | IL-2.mp. | 72997 |
| 71 | Interleukin-2.mp. | 148834 |
| 72 | or/12-71 | 4485081 |
| 73 | 11 and 72 | 7313 |
| 74 | (exp animals/ or exp animal experimentation/ or nonhuman/) not ((exp animals/ or exp animal experimentation/ or nonhuman/) and exp human/) | 7722261 |
| 75 | 73 not 74 | 7015 |

**EBM Reviews - Cochrane Central Register of Controlled Trials April 2022**

Search Strategy:

| **#** | **Searches** | **Results** |
| --- | --- | --- |
| 1 | systemic lupus erythematosus/ or Lupus Erythematosus, Systemic/ | 935 |
| 2 | (systemic adj3 lupus).mp. | 2882 |
| 3 | sle.tw,kf. | 2287 |
| 4 | or/1-3 | 3271 |
| 5 | neuro*.mp. | 131626 |
| 6 | 4 and 5 | 181 |
| 7 | Neurolupus.mp. | 0 |
| 8 | CNS lupus.mp. | 2 |
| 9 | (central nervous system adj6 systemic lupus).mp. | 0 |
| 10 | npsle.mp. | 15 |
| 11 | or/6-10 | 186 |
| 12 | exp biological product/ or Biological Products/ | 29606 |
| 13 | exp cytokine/ or exp Cytokines/ | 21504 |
| 14 | exp antibody/ or exp Antibodies/ | 27895 |
| 15 | biologics.mp. | 1609 |
| 16 | (biologic* adj3 agent*).mp. | 1297 |
| 17 | (biologic* adj3 therap*).mp. | 2994 |
| 18 | biologicals.mp. | 1161 |
| 19 | cytokine*.mp. | 20789 |
| 20 | chemokine*.mp. | 2683 |
| 21 | interferon*.mp. | 17219 |
| 22 | interleukin*.mp. | 23634 |
| 23 | lymphokine*.mp. | 268 |
| 24 | monokine*.mp. | 51 |
| 25 | antibod*.mp. | 50910 |
| 26 | autoantibod*.mp. | 2170 |
| 27 | isoantibod*.mp. | 132 |
| 28 | (inflamm* factor* adj4 mediator*).mp. | 3 |
| 29 | metalloproteinas*.mp. | 2196 |
| 30 | belimumab.mp. | 297 |
| 31 | benlysta.mp. | 25 |
| 32 | lymphostat B.mp. | 19 |
| 33 | rituximab.mp. | 5791 |
| 34 | ritemvia.mp. | 0 |
| 35 | ritumax.mp. | 1 |
| 36 | rituxan.mp. | 112 |
| 37 | rituxin.mp. | 0 |
| 38 | rituzena.mp. | 0 |
| 39 | rixathon.mp. | 6 |
| 40 | riximyo.mp. | 0 |
| 41 | ruxience.mp. | 1 |
| 42 | truxima.mp. | 8 |
| 43 | tuxella.mp. | 0 |
| 44 | anifrolumab.mp. | 106 |
| 45 | tabalumab.mp. | 42 |
| 46 | epratuzumab.mp. | 62 |
| 47 | lymphocide.mp. | 0 |
| 48 | atacicept.mp. | 89 |
| 49 | blisibimod.mp. | 27 |
| 50 | tofacitinib.mp. | 966 |
| 51 | ocrelizumab.mp. | 283 |
| 52 | abatacept.mp. | 956 |
| 53 | edratide.mp. | 3 |
| 54 | lupuzor.mp. | 4 |
| 55 | forigerimod.mp. | 2 |
| 56 | sirukumab.mp. | 117 |
| 57 | plivensia.mp. | 0 |
| 58 | tocilizumab.mp. | 1549 |
| 59 | actemra.mp. | 68 |
| 60 | atlizumab.mp. | 4 |
| 61 | lusinex.mp. | 1 |
| 62 | roactemra.mp. | 127 |
| 63 | sifalimumab.mp. | 32 |
| 64 | rontalizumab.mp. | 8 |
| 65 | ustekinumab.mp. | 1066 |
| 66 | baricitinib.mp. | 543 |
| 67 | PF-04236921.mp. | 13 |
| 68 | dapirolizumab.mp. | 17 |
| 69 | bortezomib.mp. | 2400 |
| 70 | IL-2.mp. | 2980 |
| 71 | Interleukin-2.mp. | 3273 |
| 72 | or/12-71 | 141589 |
| 73 | 11 and 72 | 70 |

# Strategies March 14, 2024

Ovid MEDLINE(R) ALL 1946 to March 13, 2024
Search Strategy:

| **#** | **Searches** | **Results** |
| --- | --- | --- |
| 1 | Lupus Erythematosus, Systemic/ | 62559 |
| 2 | (systemic adj3 lupus).mp. | 82542 |
| 3 | sle.tw,kf. | 41960 |
| 4 | or/1-3 | 86287 |
| 5 | neuro*.mp. | 2596918 |
| 6 | 4 and 5 | 5904 |
| 7 | Neurolupus.mp. | 71 |
| 8 | CNS lupus.mp. | 160 |
| 9 | (central nervous system adj6 systemic lupus).mp. | 333 |
| 10 | npsle.mp. | 702 |
| 11 | or/6-10 | 6143 |
| 12 | Biological Products/ | 36387 |
| 13 | exp Cytokines/ | 805502 |
| 14 | exp Antibodies/ | 927528 |
| 15 | biologics.mp. | 18406 |
| 16 | (biologic* adj3 agent*).mp. | 22081 |
| 17 | (biologic* adj3 therap*).mp. | 22207 |
| 18 | biologicals.mp. | 3586 |
| 19 | cytokine*.mp. | 517042 |
| 20 | chemokine*.mp. | 115481 |
| 21 | interferon*.mp. | 231603 |
| 22 | interleukin*.mp. | 424151 |
| 23 | lymphokine*.mp. | 26454 |
| 24 | monokine*.mp. | 3177 |
| 25 | antibod*.mp. | 1307229 |
| 26 | autoantibod*.mp. | 105528 |
| 27 | isoantibod*.mp. | 14453 |
| 28 | (inflamm* factor* adj4 mediator*).mp. | 73 |
| 29 | metalloproteinas*.mp. | 90392 |
| 30 | belimumab.mp. | 1128 |
| 31 | benlysta.mp. | 51 |
| 32 | lymphostat B.mp. | 8 |
| 33 | rituximab.mp. | 32142 |
| 34 | ritemvia.mp. | 1 |
| 35 | ritumax.mp. | 0 |
| 36 | rituxan.mp. | 341 |
| 37 | rituxin.mp. | 1 |
| 38 | rituzena.mp. | 0 |
| 39 | rixathon.mp. | 13 |
| 40 | riximyo.mp. | 5 |
| 41 | ruxience.mp. | 8 |
| 42 | truxima.mp. | 27 |
| 43 | tuxella.mp. | 0 |
| 44 | anifrolumab.mp. | 201 |
| 45 | tabalumab.mp. | 64 |
| 46 | epratuzumab.mp. | 254 |
| 47 | lymphocide.mp. | 1 |
| 48 | atacicept.mp. | 123 |
| 49 | blisibimod.mp. | 22 |
| 50 | tofacitinib.mp. | 3215 |
| 51 | ocrelizumab.mp. | 1006 |
| 52 | abatacept.mp. | 4583 |
| 53 | edratide.mp. | 17 |
| 54 | lupuzor.mp. | 13 |
| 55 | forigerimod.mp. | 1 |
| 56 | sirukumab.mp. | 63 |
| 57 | plivensia.mp. | 0 |
| 58 | tocilizumab.mp. | 6990 |
| 59 | actemra.mp. | 87 |
| 60 | atlizumab.mp. | 19 |
| 61 | lusinex.mp. | 1 |
| 62 | roactemra.mp. | 25 |
| 63 | sifalimumab.mp. | 45 |
| 64 | rontalizumab.mp. | 15 |
| 65 | ustekinumab.mp. | 3435 |
| 66 | baricitinib.mp. | 1548 |
| 67 | PF-04236921.mp. | 4 |
| 68 | dapirolizumab.mp. | 12 |
| 69 | bortezomib.mp. | 11357 |
| 70 | IL-2.mp. | 57689 |
| 71 | Interleukin-2.mp. | 64829 |
| 72 | or/12-71 | 2625922 |
| 73 | 11 and 72 | 2767 |
| 74 | exp animals/ not (exp animals/ and exp humans/) | 5202658 |
| 75 | 73 not 74 | 2629 |
| 76 | (202206* or 202207* or 202208* or 202209* or 20221* or 2023* or 2024*).dt,ez,da. | 3058689 |
| 77 | 75 and 76 | 259 |

**Ovid Embase Classic+Embase 1947 to 2024 March 13**

Search Strategy:

| **#** | **Searches** | **Results** |
| --- | --- | --- |
| 1 | systemic lupus erythematosus/ | 123504 |
| 2 | (systemic adj3 lupus).mp. | 137693 |
| 3 | sle.tw,kf. | 73123 |
| 4 | or/1-3 | 147669 |
| 5 | neuro*.mp. | 3814298 |
| 6 | 4 and 5 | 14857 |
| 7 | Neurolupus.mp. | 177 |
| 8 | CNS lupus.mp. | 290 |
| 9 | (central nervous system adj6 systemic lupus).mp. | 440 |
| 10 | npsle.mp. | 1318 |
| 11 | or/6-10 | 15223 |
| 12 | exp biological product/ | 968832 |
| 13 | exp cytokine/ | 1974996 |
| 14 | exp antibody/ | 1667386 |
| 15 | biologics.mp. | 37813 |
| 16 | (biologic* adj3 agent*).mp. | 32582 |
| 17 | (biologic* adj3 therap*).mp. | 47921 |
| 18 | biologicals.mp. | 8497 |
| 19 | cytokine*.mp. | 834330 |
| 20 | chemokine*.mp. | 204931 |
| 21 | interferon*.mp. | 496140 |
| 22 | interleukin*.mp. | 929219 |
| 23 | lymphokine*.mp. | 17924 |
| 24 | monokine*.mp. | 2816 |
| 25 | antibod*.mp. | 1829722 |
| 26 | autoantibod*.mp. | 128628 |
| 27 | isoantibod*.mp. | 862 |
| 28 | (inflamm* factor* adj4 mediator*).mp. | 91 |
| 29 | metalloproteinas*.mp. | 135828 |
| 30 | belimumab.mp. | 4463 |
| 31 | benlysta.mp. | 298 |
| 32 | lymphostat B.mp. | 105 |
| 33 | rituximab.mp. | 121297 |
| 34 | ritemvia.mp. | 32 |
| 35 | ritumax.mp. | 0 |
| 36 | rituxan.mp. | 3440 |
| 37 | rituxin.mp. | 36 |
| 38 | rituzena.mp. | 20 |
| 39 | rixathon.mp. | 82 |
| 40 | riximyo.mp. | 42 |
| 41 | ruxience.mp. | 46 |
| 42 | truxima.mp. | 167 |
| 43 | tuxella.mp. | 1 |
| 44 | anifrolumab.mp. | 608 |
| 45 | tabalumab.mp. | 322 |
| 46 | epratuzumab.mp. | 1542 |
| 47 | lymphocide.mp. | 78 |
| 48 | atacicept.mp. | 755 |
| 49 | blisibimod.mp. | 170 |
| 50 | tofacitinib.mp. | 10962 |
| 51 | ocrelizumab.mp. | 4773 |
| 52 | abatacept.mp. | 13439 |
| 53 | edratide.mp. | 71 |
| 54 | lupuzor.mp. | 37 |
| 55 | forigerimod.mp. | 61 |
| 56 | sirukumab.mp. | 411 |
| 57 | plivensia.mp. | 3 |
| 58 | tocilizumab.mp. | 29631 |
| 59 | actemra.mp. | 783 |
| 60 | atlizumab.mp. | 568 |
| 61 | lusinex.mp. | 1 |
| 62 | roactemra.mp. | 287 |
| 63 | sifalimumab.mp. | 291 |
| 64 | rontalizumab.mp. | 160 |
| 65 | ustekinumab.mp. | 14068 |
| 66 | baricitinib.mp. | 5802 |
| 67 | PF-04236921.mp. | 44 |
| 68 | dapirolizumab.mp. | 52 |
| 69 | bortezomib.mp. | 42694 |
| 70 | IL-2.mp. | 78284 |
| 71 | Interleukin-2.mp. | 164366 |
| 72 | or/12-71 | 4986075 |
| 73 | 11 and 72 | 8501 |
| 74 | (exp animals/ or exp animal experimentation/ or nonhuman/) not ((exp animals/ or exp animal experimentation/ or nonhuman/) and exp human/) | 8179990 |
| 75 | 73 not 74 | 8171 |
| 76 | limit 75 to dc=20220601-20240314 | 1196 |

**Ovid EBM Reviews - Cochrane Central Register of Controlled Trials February 2024**

Search Strategy:

| **#** | **Searches** | **Results** |
| --- | --- | --- |
| 1 | systemic lupus erythematosus/ or Lupus Erythematosus, Systemic/ | 1267 |
| 2 | (systemic adj3 lupus).mp. | 3068 |
| 3 | sle.tw,kf. | 2410 |
| 4 | or/1-3 | 3468 |
| 5 | neuro*.mp. | 145637 |
| 6 | 4 and 5 | 164 |
| 7 | Neurolupus.mp. | 0 |
| 8 | CNS lupus.mp. | 3 |
| 9 | (central nervous system adj6 systemic lupus).mp. | 0 |
| 10 | npsle.mp. | 14 |
| 11 | or/6-10 | 171 |
| 12 | exp biological product/ or Biological Products/ | 36924 |
| 13 | exp cytokine/ or exp Cytokines/ | 27352 |
| 14 | exp antibody/ or exp Antibodies/ | 37235 |
| 15 | biologics.mp. | 1798 |
| 16 | (biologic* adj3 agent*).mp. | 1094 |
| 17 | (biologic* adj3 therap*).mp. | 3125 |
| 18 | biologicals.mp. | 1155 |
| 19 | cytokine*.mp. | 22955 |
| 20 | chemokine*.mp. | 2867 |
| 21 | interferon*.mp. | 17309 |
| 22 | interleukin*.mp. | 25202 |
| 23 | lymphokine*.mp. | 277 |
| 24 | monokine*.mp. | 53 |
| 25 | antibod*.mp. | 55994 |
| 26 | autoantibod*.mp. | 2338 |
| 27 | isoantibod*.mp. | 164 |
| 28 | (inflamm* factor* adj4 mediator*).mp. | 4 |
| 29 | metalloproteinas*.mp. | 2401 |
| 30 | belimumab.mp. | 357 |
| 31 | benlysta.mp. | 22 |
| 32 | lymphostat B.mp. | 19 |
| 33 | rituximab.mp. | 5983 |
| 34 | ritemvia.mp. | 0 |
| 35 | ritumax.mp. | 1 |
| 36 | rituxan.mp. | 114 |
| 37 | rituxin.mp. | 0 |
| 38 | rituzena.mp. | 0 |
| 39 | rixathon.mp. | 11 |
| 40 | riximyo.mp. | 0 |
| 41 | ruxience.mp. | 5 |
| 42 | truxima.mp. | 13 |
| 43 | tuxella.mp. | 0 |
| 44 | anifrolumab.mp. | 136 |
| 45 | tabalumab.mp. | 41 |
| 46 | epratuzumab.mp. | 56 |
| 47 | lymphocide.mp. | 0 |
| 48 | atacicept.mp. | 93 |
| 49 | blisibimod.mp. | 25 |
| 50 | tofacitinib.mp. | 1102 |
| 51 | ocrelizumab.mp. | 337 |
| 52 | abatacept.mp. | 1028 |
| 53 | edratide.mp. | 2 |
| 54 | lupuzor.mp. | 3 |
| 55 | forigerimod.mp. | 1 |
| 56 | sirukumab.mp. | 108 |
| 57 | plivensia.mp. | 0 |
| 58 | tocilizumab.mp. | 1655 |
| 59 | actemra.mp. | 82 |
| 60 | atlizumab.mp. | 3 |
| 61 | lusinex.mp. | 1 |
| 62 | roactemra.mp. | 132 |
| 63 | sifalimumab.mp. | 31 |
| 64 | rontalizumab.mp. | 6 |
| 65 | ustekinumab.mp. | 1170 |
| 66 | baricitinib.mp. | 705 |
| 67 | PF-04236921.mp. | 13 |
| 68 | dapirolizumab.mp. | 25 |
| 69 | bortezomib.mp. | 2458 |
| 70 | IL-2.mp. | 3187 |
| 71 | Interleukin-2.mp. | 3077 |
| 72 | or/12-71 | 159201 |
| 73 | 11 and 72 | 65 |
| 74 | ("202206" or "202207" or "202208" or "202209" or 20221* or 2023* or 2024*).up. | 919796 |
| 75 | ("2022" or "2023" or "2024").dl,yr. | 304659 |
| 76 | 74 or 75 | 953118 |
| 77 | 73 and 76 | 41 |

**Manuscript Abbreviations**

| **Abbreviation** | **Definition** |
| --- | --- |
| Aβ42 | Amyloid-beta 42 |
| Ab | Antibody |
| ACR | American College of Rheumatology |
| ACS | Acute confusional state |
| AGA | Anti-ganglioside antibody |
| ANFA | Anti-neuronal filament antibody |
| anti-aCL | Anticardiolipin antibody |
| anti-AQP4 | Anti-aquaporin-4 antibody |
| anti-GAPDH | Anti-glyceraldehyde-3-phosphate dehydrogenase antibody |
| anti-GFAP | Anti-glial fibrillary acidic protein antibody |
| anti-GP2 | Anti-glycoprotein 2 antibody |
| anti-GRP78 | Anti-glucose-regulated protein 78 antibody |
| anti-MAA | Anti-malondialdehyde acetaldehyde antibody |
| anti-MAG | Anti-myelin-associated glycoprotein antibody |
| anti-MOG | Anti-myelin oligodendrocyte glycoprotein antibody |
| anti-mNR1 | Anti-NMDA receptor NR1 antibody |
| anti-NMDAR | Anti-N-methyl-D-aspartate receptor antibody |
| anti-NR1 | Anti-NMDA receptor NR1 antibody |
| anti-NR2 | Anti-NMDA receptor NR2 antibody |
| anti-NR2/NMDAR | Anti-NMDA receptor NR2 antibody |
| anti-Ribo P | Anti-ribosomal P antibody |
| anti-SBSN | Anti-suprabasin antibody |
| anti-Sm | Anti-Smith antibody |
| anti-UCH-L1 | Anti-ubiquitin C-terminal hydrolase L1 antibody |
| aPL | Antiphospholipid antibody |
| APP | Amyloid precursor protein |
| APRIL | A Proliferation-Inducing Ligand |
| AQP4 | Aquaporin-4 |
| AUC | Area under the curve |
| BAFF | B-cell activating factor |
| BBB | Blood–brain barrier |
| C3 | Complement component 3 |
| C4 | Complement component 4 |
| CCL2 | C-C motif chemokine ligand 2 |
| CCL3 | C-C motif chemokine ligand 3 |
| CCL4 | C-C motif chemokine ligand 4 |
| CCL11 | C-C motif chemokine ligand 11 (Eotaxin) |
| CI | Cognitive impairment |
| CNS | Central nervous system |
| CSF | Cerebrospinal fluid |
| CVD | Cerebrovascular disease |
| ELISA | Enzyme-linked immunosorbent assay |
| FGF | Fibroblast growth factor |
| FGF-b (bFGF) | Basic fibroblast growth factor |
| GAPDH | Glyceraldehyde-3-phosphate dehydrogenase |
| G-CSF | Granulocyte colony-stimulating factor |
| GFAP | Glial fibrillary acidic protein |
| GM-CSF | Granulocyte-macrophage colony-stimulating factor |
| GP2 | Glycoprotein 2 |
| GRP78 | Glucose-regulated protein 78 |
| GSH | Reduced glutathione |
| HC | Healthy control |
| HMGB1 | High mobility group box 1 |
| HR | Hazard ratio |
| HRQoL | Health-related quality of life |
| IFN | Interferon |
| IFN-α | Interferon alpha |
| IFN-γ | Interferon gamma |
| IgG | Immunoglobulin G |
| IgM | Immunoglobulin M |
| IL | Interleukin |
| IL-1α | Interleukin-1 alpha |
| IL-1β | Interleukin-1 beta |
| IL-1ra | Interleukin-1 receptor antagonist |
| IL-2 | Interleukin-2 |
| IL-4 | Interleukin-4 |
| IL-5 | Interleukin-5 |
| IL-6 | Interleukin-6 |
| IL-7 | Interleukin-7 |
| IL-8 | Interleukin-8 |
| IL-9 | Interleukin-9 |
| IL-10 | Interleukin-10 |
| IL-12 | Interleukin-12 |
| IL-12p40 | Interleukin-12 p40 subunit |
| IL-12p70 | Interleukin-12 p70 |
| IL-13 | Interleukin-13 |
| IL-15 | Interleukin-15 |
| IL-17 | Interleukin-17 |
| IP-10 | Interferon gamma-induced protein 10 (CXCL10) |
| I² | I-squared statistic |
| LA | Lupus anticoagulant |
| MAP | Multiplex assay panel |
| MCP-1 | Monocyte chemoattractant protein-1 |
| M-CSF | Macrophage colony-stimulating factor |
| MDC | Macrophage-derived chemokine (CCL22) |
| MEDLINE | Medical Literature Analysis and Retrieval System Online |
| MFI | Median fluorescence intensity |
| MIG | Monokine induced by interferon gamma (CXCL9) |
| MMP | Matrix metalloproteinase |
| MMP-2 | Matrix metalloproteinase-2 |
| MMP-9 | Matrix metalloproteinase-9 |
| MPO | Myeloperoxidase |
| MS | Multiple sclerosis |
| NfL | Neurofilament light chain |
| NGAL | Neutrophil gelatinase-associated lipocalin |
| NMDA | N-methyl-D-aspartate |
| NMO | Neuromyelitis optica |
| NOx | Nitric oxide metabolites |
| NOS | Newcastle–Ottawa Scale |
| NP | Neuropsychiatric |
| NPSLE | Neuropsychiatric systemic lupus erythematosus |
| OR | Odds ratio |
| PAI-1 | Plasminogen activator inhibitor-1 |
| PDGF-BB | Platelet-derived growth factor-BB |
| PI | Prediction interval *(or Plasminogen inhibitor? In your manuscript PI is used as prediction interval.)* |
| PNP | Peripheral neuropathy |
| PNS | Peripheral nervous system |
| PRISMA | Preferred Reporting Items for Systematic Reviews and Meta-Analyses |
| Q-albumin | Albumin quotient |
| RANTES | Regulated upon Activation, Normal T-cell Expressed and Secreted (CCL5) |
| REML | Restricted maximum likelihood |
| S100A8/A9 | Calprotectin |
| S100B | S100 calcium-binding protein B |
| SBSN | Suprabasin |
| Se | sensitivity |
| Sp | Specificity |
| SLE | Systemic lupus erythematosus |
| SLICC | Systemic Lupus International Collaborating Clinics |
| TGF-β | Transforming growth factor beta |
| TNF-α | Tumor necrosis factor alpha |
| TWEAK | TNF-like weak inducer of apoptosis |
| UCH-L1 | Ubiquitin C-terminal hydrolase L1 |
| uPA | Urokinase-type plasminogen activator |
| VCAM-1 | Vascular cell adhesion molecule-1 |
| VEGF | Vascular endothelial growth factor |

**PRISMA Check List**

| **Section and Topic** | **Item #** | **Checklist item** | **Location where item is reported** |
| --- | --- | --- | --- |
| **TITLE** | | |  |
| Title | 1 | Identify the report as a systematic review. | Page 1 |
| **ABSTRACT** | | |  |
| Abstract | 2 | See the PRISMA 2020 for Abstracts checklist. | Page 2 |
| **INTRODUCTION** | | |  |
| Rationale | 3 | Describe the rationale for the review in the context of existing knowledge. | Pages 3-4 (introduction) |
| Objectives | 4 | Provide an explicit statement of the objective(s) or question(s) the review addresses. | Page 3 (last paragraph of the introduction) |
| **METHODS** | | |  |
| Eligibility criteria | 5 | Specify the inclusion and exclusion criteria for the review and how studies were grouped for the syntheses. | Pages 3-4 |
| Information sources | 6 | Specify all databases, registers, websites, organisations, reference lists and other sources searched or consulted to identify studies. Specify the date when each source was last searched or consulted. | Page 3 (literature search) |
| Search strategy | 7 | Present the full search strategies for all databases, registers and websites, including any filters and limits used. | Supplementary File 1 |
| Selection process | 8 | Specify the methods used to decide whether a study met the inclusion criteria of the review, including how many reviewers screened each record and each report retrieved, whether they worked independently, and if applicable, details of automation tools used in the process. | Page 4 |
| Data collection process | 9 | Specify the methods used to collect data from reports, including how many reviewers collected data from each report, whether they worked independently, any processes for obtaining or confirming data from study investigators, and if applicable, details of automation tools used in the process. | Page 4 |
| Data items | 10a | List and define all outcomes for which data were sought. Specify whether all results that were compatible with each outcome domain in each study were sought (e.g. for all measures, time points, analyses), and if not, the methods used to decide which results to collect. | Page 4 |
|  | 10b | List and define all other variables for which data were sought (e.g. participant and intervention characteristics, funding sources). Describe any assumptions made about any missing or unclear information. | Pages 4-5 |
| Study risk of bias assessment | 11 | Specify the methods used to assess risk of bias in the included studies, including details of the tool(s) used, how many reviewers assessed each study and whether they worked independently, and if applicable, details of automation tools used in the process. | Page 4 |
| Effect measures | 12 | Specify for each outcome the effect measure(s) (e.g. risk ratio, mean difference) used in the synthesis or presentation of results. | Pages 4-5 |
| Synthesis methods | 13a | Describe the processes used to decide which studies were eligible for each synthesis (e.g. tabulating the study intervention characteristics and comparing against the planned groups for each synthesis (item #5)). | Pages 4-5 |
|  | 13b | Describe any methods required to prepare the data for presentation or synthesis, such as handling of missing summary statistics, or data conversions. | Page 5 |
|  | 13c | Describe any methods used to tabulate or visually display results of individual studies and syntheses. | Pages 5-16; Tables 1-11; Figures 1-7 |
|  | 13d | Describe any methods used to synthesize results and provide a rationale for the choice(s). If meta-analysis was performed, describe the model(s), method(s) to identify the presence and extent of statistical heterogeneity, and software package(s) used. | Pages 4-5 |
|  | 13e | Describe any methods used to explore possible causes of heterogeneity among study results (e.g. subgroup analysis, meta-regression). | Pages 10-16 |
|  | 13f | Describe any sensitivity analyses conducted to assess robustness of the synthesized results. | Pages, 10-16 (leave-one out, exclusion analyses) |
| Reporting bias assessment | 14 | Describe any methods used to assess risk of bias due to missing results in a synthesis (arising from reporting biases). | Page 5 (planned methods);Page 13 and Figure 6B (funnel plot/small-study effects for anti-ribosomal P) |
| Certainty assessment | 15 | Describe any methods used to assess certainty (or confidence) in the body of evidence for an outcome. | Not performed (no formal certainty assessment such as GRADE) |
| **RESULTS** | | |  |
| Study selection | 16a | Describe the results of the search and selection process, from the number of records identified in the search to the number of studies included in the review, ideally using a flow diagram. | Page 5; Figure 1 |
|  | 16b | Cite studies that might appear to meet the inclusion criteria, but which were excluded, and explain why they were excluded. | Figure 1 |
| Study characteristics | 17 | Cite each included study and present its characteristics. | Page 5; Tables 1-11 |
| Risk of bias in studies | 18 | Present assessments of risk of bias for each included study. | Page 5; Supplementary Files 2-4 (NOS assessments) |
| Results of individual studies | 19 | For all outcomes, present, for each study: (a) summary statistics for each group (where appropriate) and (b) an effect estimate and its precision (e.g. confidence/credible interval), ideally using structured tables or plots. | Pages 5-16; Tables 1-11 |
| Results of syntheses | 20a | For each synthesis, briefly summarise the characteristics and risk of bias among contributing studies. | Pages 10-16 |
|  | 20b | Present results of all statistical syntheses conducted. If meta-analysis was done, present for each the summary estimate and its precision (e.g. confidence/credible interval) and measures of statistical heterogeneity. If comparing groups, describe the direction of the effect. | Pages 10-16; figures 2-7 |
|  | 20c | Present results of all investigations of possible causes of heterogeneity among study results. | Pages 10-16 |
|  | 20d | Present results of all sensitivity analyses conducted to assess the robustness of the synthesized results. | Pages 10-16 |
| Reporting biases | 21 | Present assessments of risk of bias due to missing results (arising from reporting biases) for each synthesis assessed. | Page 13; Figure 6B |
| Certainty of evidence | 22 | Present assessments of certainty (or confidence) in the body of evidence for each outcome assessed. | Not assessed (no formal certainty assessment) |
| **DISCUSSION** | | |  |
| Discussion | 23a | Provide a general interpretation of the results in the context of other evidence. | Discussion |
|  | 23b | Discuss any limitations of the evidence included in the review. | Discussion |
|  | 23c | Discuss any limitations of the review processes used. | Discussion |
|  | 23d | Discuss implications of the results for practice, policy, and future research. | Discussion and conclusion |
| **OTHER INFORMATION** | | |  |
| Registration and protocol | 24a | Provide registration information for the review, including register name and registration number, or state that the review was not registered. | Page 3 ("This systematic review was not prospectively registered.") |
|  | 24b | Indicate where the review protocol can be accessed, or state that a protocol was not prepared. | Page 3 ("The review methodology... was established before study selection and data synthesis.") |
|  | 24c | Describe and explain any amendments to information provided at registration or in the protocol. | Not applicable (no registered protocol) |
| Support | 25 | Describe sources of financial or non-financial support for the review, and the role of the funders or sponsors in the review. | Funding/Support section |
| Competing interests | 26 | Declare any competing interests of review authors. | Competing Interests/Declaration section |
| Availability of data, code and other materials | 27 | Report which of the following are publicly available and where they can be found: template data collection forms; data extracted from included studies; data used for all analyses; analytic code; any other materials used in the review. | Data Availability statement |

*From:*  Page MJ, McKenzie JE, Bossuyt PM, Boutron I, Hoffmann TC, Mulrow CD, et al. The PRISMA 2020 statement: an updated guideline for reporting systematic reviews. BMJ 2021;372:n71. doi: 10.1136/bmj.n71. This work is licensed under CC BY 4.0. To view a copy of this license, visit <https://creativecommons.org/licenses/by/4.0/>
