# Supplementary material for: The neuropsychiatric systemic lupus erythematosus biomarker landscape: what blood and cerebrospinal fluid analytes have been studied and how they performed? A systematic review and meta-analysis
Source: Front Immunol. 2026 Oct 1;17:1903757. doi: 10.3389/fimmu.2026.1903757 (PMC13668822; doi:10.3389/fimmu.2026.1903757)
Supplement: Supplementary file 2 [file SupplementaryFile2.docx]

**Cross-sectional studies quality assessment [ adapted from Newcastle-Ottawa Scale]**

| **Author / year** | **Analyte and matrix (Serum, plasma, CSF)** | **Representativeness of the sample**  **[Representative of the average in the target population]** | **Sample size**  **[Justified and satisfactory]** | **Non respondents**  **[Analyte comparability between NPSLE and non-NPSLE characteristics is established, and the response rate is satisfactory (≥90% of patients have the analyte [Ab, cytokine, or molecule] levels available)].** | **Ascertainment of exposure**  **[Appropriate measurement of the analyte levels (assay and features/performance of the assay -matrix, CV, Sensitivity, etc.-)]** | **Comparability**  **[NPSLE and non-NPSLE were comparable in terms of demographic (main=age) and clinical characteristics]** | **Assessment of outcome**  **NPSLE manifestation(s) defined by accepted methods/tests/tools/clinically by expert (when apply)** | **Statistical test**  **Statistical test used to analyze the data clearly described, appropriate, and measures of association presented including confidence intervals and probability level (P value)** | **Total score** | **Quality** |
| --- | --- | --- | --- | --- | --- | --- | --- | --- | --- | --- |
| **Abdel-Nasser/**  **2008**^1^ | **Anti-Ribo P (aRP)**  **Serum** | ***** | **No justified** | ***** | *****  **Appropriate measurement (assay) of the analyte levels but no further description** | ***** | ***** | ***** | **6/10** | **Satisfactory** |
| **Abdul-Sattar /2013**^2^ | **Anti-cardiolipin (aCL)**  **anti-La**  **anti-Ro**  **Serum**  **Lupus anticoagulant (LAC)-Plasma** | ***** | **No justified** | ***** | *****  **Appropriate measurement (assay) of the analyte levels but no further description** | ***** | ***** | ***** | **6/10** | **Satisfactory** |
| **Afeltra/**  **2003**^3^ | **Anti-cardiolipin (aCL)**  **anti-phosphatidylserine,**  **anti-phosphatidylinositol anti-phosphatidic acid**  **anti-2-glycoprotein I**  **Serum**  **Lupus anticoagulant (LAC)-Plasma** | ***** | **No justified** | ***** | *****  **Appropriate measurement (assay) of the analyte levels but no further description** | ***** | ***** | ***** | **6/10** | **Satisfactory** |
| **Ahmadzade/**  **2024**^4^ | **Neurofilament (nfl), HMGB1, MMP-9**  **Serum** | **No description of the derivation of the included subjects (? All consecutive patient)** | **No justified** | **No description of the response rate or the characteristics of the responders and the non-responders** | *****  **Appropriate measurement (assay) of the analyte levels but no further description** | ***** | **No accepted method for CI (MoCA)** | ***** | **3/10** | **Unsatisfactory** |
| **Arinuma/**  **2019**^5^ | **Anti-Ribo P**  **Anti-dsDNA**  **Anti-Sm**  **Anti-cardiolipin**  **Anti-β2-glycoprotein I**  **Lupus anticoagulant**  **Serum**  ***Mortality** | ***** | **No justified** | ***** | *****  **Appropriate measurement (assay) of the analyte levels but no further description** | **Control group was not non-NPSLE** | ***** | ***** | **5/10** | **Satisfactory** |
| **Arinuma/**  **2014**^6^ | **Anti-Ribo P**  **Anti-dsDNA**  **Anti-Sm**  **Anti-cardiolipin (CL)**  **Anti-CL β2-glycoprotein I**  **Lupus anticoagulant**  **IL-6**  **CSF and serum**  ***Vs changes in MRI** | ***** | **No justified** | ***** | *****  **Appropriate measurement (assay) of the analyte levels but no further description** | **Control group was not non-NPSLE** | ****** | ***** | **5/10** | **Satisfactory** |
| **Arinuma/**  **2008**^7^ | **Anti-NR2**  **Serum and CSF** | ***** | **No justified** | ***** | ****** | **Comparison group was not SLE-non NPSLE** | ***** | ***** | **6/10** | **Satisfactory** |
| **Asano/**  **2017**^8^ | **Quotient of alpha2 macroglobulin (Qa2MG)**  **C3**  **IL-6**  **CSF** | **No description of the derivation of the included subjects** | **No justified** | ***** | ****** | ***** | ***** | ***** | **5/10** | **Satisfactory** |
| **Balajkova/ 2020**^9^ | **TWEEK**  **Serum** | ***** | **No justified** | ***** | ****** | ***** | ***** | ***** | **7/10** | **Good** |
| **Baraczka/**  **2004**^10^ | **IL-6**  **IL-10**  **TNF-a**  **Serum and CSF** | ***** | **No described/no justified** | ***** | *****  **Appropriate measurement (assay) of the analyte levels but no further description** | **Comparison MS no other SLE without NP** | ***** | ***** | **5/10** | **Satisfactory** |
| **Bluestein /1981**^11^ | **Anti-neuronal Ab**  **CSF** | ***** | **No justified** | ***** | ****** | ***** | ***** | **No P levels or statistical descriptors for main outcome, only description of levels** | **6/10** | **Satisfactory** |
| **De Sousa/**  **2023**^12^ | **L-6, S100ß, myeloperoxidase (MPO), malondialdehyde and**  **reduced glutathione (GSH)**  **Plasma** | ***** | ***** | ***** | *****  **Appropriate measurement (assay) of the analyte levels but no further description** | ****** | ***** | ***** | **8/10** | **Good** |
| **Dellalibera-Joviliano/**  **2003**^13^ | **Low (LKg) and high molecular weight kininogen (HKg)**  **IL-1ß**  **IL-6**  **IL-8**  **IL-10**  **TNF-α**  **Plasma and CSF** | **No description of the derivation of the included subjects** | **No justified** | ***** | ****** | **Control group no SLE** | ***** | ***** | **5/10** | **Satisfactory** |
| **Denburg/**  **1997**^14^ | **Lupus anticoagulant**  **Plasma** | ***** | **No justified** | ***** | ****** | *****  **Comparisons made between LA + and – patients, no between NPSLE and non-NPSLE** | ***** | ***** | **7/10** | **Good** |
| **Fujieda/**  **2019**^15^ | **Anti-NR2**  **Serum** | ***** | **No justified** | ***** | *****  **Appropriate measurement (assay) of the analyte levels but no further description** | **Comparison post steroids NP (PSNP) or HC, no SLE without NPSLE** | ***** | ***** | **6/10** | **Satisfactory** |
| **George-Chandy/**  **2008**^16^ | **BAFF and APRIL**  **CSF and serum** | ***** | ***** | ***** | ****** | **No information to determine whether NPSLE and non-NPSLE were comparable in terms of demographic and clinical characteristics** | ****** | ***** | **8/10** | **Good** |
| **Gerli/**  **2002**^17^ | **Anti-Ribo P**  **Serum** | ***** | ***** | ***** | *****  **Appropriate measurement (assay) of the analyte levels but no further description** | ***** | ***** | ***** | **7/10** | **Good** |
| **Gono/**  **2011**^18^ | **Anti-NR2**  **Serum** | **No description of the derivation of the included subjects** | **No justified** | ***** | ****** | ***** | ****** | ***** | **7/10** | **Good** |
| **Guo/**  **2022**^19^ | **Anti-UCH**  **Serum** | ***** | **No justified** | ******* | ****** | ***** | ***** | ***** | **7/10** | **Good** |
| **Hanly/**  **2022**^20^ | **Anti-dsDNA**  **Anti-chromatin**  **Anti-ribosomal P**  **Anti-Ro (60, 52)**  **Anti-La**  **Anti-Sm**  **Anti-RNP (A, 68)**  **Anticardiolipin**  **Anti-β2 glycoprotein I**  **LAC**  **Anticardiolipin or anti-β2 glycoprotein I or LAC**  **Anti-NR2 (peptide mix)**  **Anti-NR2 (protein mix)**  **Anti-GFAP**  **Anti-MPP1**  **C3 and C4**  **Elevated neurofilament light chains**  **Plasma** | ***** | ***** | ***** | *****  **No CV, Sensitivity provided** | ****** | ****** | ***** | **9/10** | **Very good** |
| **Harrison/**  **2006**^21^ | **Anti-NR2**  **Serum** | ***** | ***** | ******* | *****  **Appropriate measurement (assay) of the analyte levels but no further description** | ***** | ***** | ***** | **7/10** | **Good** |
| **Hawro/**  **2015**^22^ | **aCL**  **LAC**  **Anti-B2GPI**  **Serum** | ***** | ***** | ******* | *****  **Appropriate measurement (assay) of the analyte levels but no further description** | ***** | ***** | ***** | **7/10** | **Good** |
| **Herranz/**  **1994**^23^ | **aCL**  **LAC**  **Serum** | ***** | ***** | **< 90% Tested for LAC in the NPSLE. < 90% tested for aCL and LAC in the non NPSLE** | *****  **Appropriate measurement (assay) of the analyte levels but no further description** | ***** | ***** | ***** | **6/10** | **Satisfactory** |
| **Hirohata/**  **2007**^24^ | **Anti-Ribo P=**  **anti-PC22**  **anti-whole P**  **anti-PEX.C22**  **CSF and Serum** | **No description of the derivation of the included subjects** | **No justified** | ******* | *****  **Appropriate measurement (assay) of the analyte levels but no further description** | ***** | ***** | ***** | **5/10** | **Satisfactory** |
| **Hirohata/**  **2014**^25^ | **Anti-NR2**  **CSF** | ***** | **No justified** | ***** | *****  **Appropriate measurement (assay) of the analyte levels but no further description** | **Control group no SLE** | ***** | ***** | **5/10** | **Satisfactory** |
| **Hirohata/**  **2009**^26^ | **IL-6**  **CSF** | ***** | **No justified** | ******* | *****  **Appropriate measurement (assay) of the analyte levels but no further description** | **Control group 13 SLE with NP manifestations but retrospective review no related to lupus. No comparison with SLE no NP** | ***** | **Limited statistics. Only ROC** | **4/10** | **Unsatisfactory** |
| **Hirohata/**  **2021**^27^ | **IL-6**  **CSF**  **Serum** | ***** | **No justified** | ******* | *****  **Appropriate measurement (assay) of the analyte levels but no further description** | **Control group no SLE** | ***** | ***** | **5/10** | **Satisfactory** |
| **Hirohata/**  **2018**^28^ | **Anti-Sm**  **Anti-Ribo P**  **aCL IgG**  **Serum** | **No description of the derivation of the included subjects (? All patient that presented with NPSLE)** | **No justified** | ******* | *****  **Appropriate measurement (assay) of the analyte levels but no further description** | **Control group no SLE** | ***** | ***** | **4/10** | **Unsatisfactory** |
| **Hirohata/**  **2014**^29^ | **Anti-Sm**  **Anti-RNP**  **Serum and CSF** | ***** | **No justified** | ******* | *****  **Appropriate measurement (assay) of the analyte levels but no further description** | **No information to determine whether NPSLE and non-NPSLE were comparable in terms of demographic and clinical characteristics** | ***** | ***** | **4/10** | **Unsatisfactory** |
| **Hirohata/**  **2019**^30^ | **Anti-NR1/NR2 (In vitro reactivity)**  **Anti-NR2**  **CSF and serum** | **No description of the derivation of the included subjects** | **No justified** | ******* | *****  **Appropriate measurement (assay) of the analyte levels but no further description** | **Control group no SLE** | ***** | ***** | **4/10** | **Unsatisfactory** |
| **Hoang/**  **2022**^31^ | **Anti-suprabasin antibodies**  **Serum** | ***** | ***** | ******* | *****  **Appropriate measurement (assay) of the analyte levels but no further description** | ***** | ***** | ***** | **7/10** | **Good** |
| **Hopia/**  **2011**^32^ | **APRIL**  **BAFF**  **Plasma and CSF** | ***** | **No justified** | **No description of the response rate or the characteristics of the responders and the non-responders** | *****  **Appropriate measurement (assay) of the analyte levels but no further description** | **Control group no SLE** | ***** | ***** | **4/10** | **Unsatisfactory** |
| **How/**  **1985**^33^ | **Anti-Neuronal**  **(Reactivity to Neuroblastoma and glioma cell lines)**  **Serum** | ***** | ***** | ******* | *****  **Appropriate measurement (assay) of the analyte levels but no further description** | ***** | ***** | ***** | **7/10** | **Good** |
| **Huang/**  **2020**^34^ | **High mobility group box 1 (HMGB1)**  **Serum**  **CSF (In a proportion)** | ***** | **ND** | ***** | *****  **No CV, Sensitivity provided** | ***** | **No description (Mentioned as per ACR, but no methods)** | ***** | **5/10** | **Satisfactory** |
| **Ichinose/**  **2016**^35^ | **IL-1b, IL-1 receptor antagonist (IL-1ra), IL-2, -4, -5, -6,**  **and -7, C-X-C motif ligand (CXCL)8/IL-8, IL-9, -10, and -12 (p70),**  **IL-13, -15, and -17A, b-FGF, C-C motif ligand (CCL)11/eotaxin, gran-**  **ulocyte colony stimulating factor (G-CSF), granulocyte–macro-**  **phage colony stimulating factor (GM-CSF), IFN-c, CXCL10/**  **nducible protein-10, CCL2/macrophage chemoattractant protein**  **(MCP)-1, CCL3/macrophage inflammatory protein (MIP)-1a**  **,**  **CCL4/MIP-1b, platelet-derived growth factor (PDGF)-BB, CCL5/reg-**  **ulated on activation, normal T cell expressed and secreted**  **(RANTES), TNF-a, and vascular endothelial growth factor (VEGF)**  **CSF** | **No description of the derivation of the included subjects (? All patient that presented with NPSLE)** | **Not justified** | ***** | ****** | ****** | ***** | ***** | **7/10** | **Good** |
| **Ichinose/**  **2015**^36^ | **IL-1b, IL-1 receptor antagonist (IL-1ra), IL-2, -4, -5, -6,**  **and -7, C-X-C motif ligand (CXCL)8/IL-8, IL-9, -10, and -12 (p70),**  **IL-13, -15, and -17A, b-FGF, C-C motif ligand (CCL)11/eotaxin, gran-**  **ulocyte colony stimulating factor (G-CSF), granulocyte–macro-**  **phage colony stimulating factor (GM-CSF), IFN-c, CXCL10/**  **nducible protein-10, CCL2/macrophage chemoattractant protein**  **(MCP)-1, CCL3/macrophage inflammatory protein (MIP)-1a**  **,**  **CCL4/MIP-1b, platelet-derived growth factor (PDGF)-BB, CCL5/reg-**  **ulated on activation, normal T cell expressed and secreted**  **(RANTES), TNF-a, and vascular endothelial growth factor (VEGF)**  **CSF** | **No description of the derivation of the included subjects (? All patient that presented with NPSLE)** | **Not justified** | ***** | ****** | **Information was not provided** | ***** | ***** | **5/10** | **Satisfactory** |
| **Iikuni/**  **2006**^37^ | **MCP-1/CCL2**  **CSF** | **No description of the derivation of the included subjects (? All consecutive patient)** | **Not justified** | **No description of the response rate or the characteristics of the responders and the non-responders** | *****  **Appropriate measurement (assay) of the analyte levels but no further description** | **Information was not provided** | *****  **Presence of NPSLE manifestation as per record linkage** | ***** | **3/10** | **Unsatisfactory** |
| **Isshi /**  **1996**^38^  **Has other paper from 1998 with same patients** | **Anti-Ribo P**  **Anti-Neural Ab**  **Serum**  **CSF (In a proportion of patients)** | **No description of the derivation of the included subjects** | **Not justified** | **No description of the response rate or the characteristics of the responders and the non-responders** | ****** | **Information was not provided** | ***** | **Incomplete** | **3/10** | **Unsatisfactory** |
| **Jönsen/**  **2003**^39^ | **IFN-a**  **IL-6**  **IL-10**  **Soluble terminal**  **complement complexes (TCC)***  **Anti-Ribo P**  **Anti-CL**  **CSF**  **Serum**  ***Plasma** | ***** | ***** | **No description** | *****  **Appropriate measurement (assay) of the analyte levels but no further description** | **Information was not provided** | ***** | ***** | **5/10** | **Satisfactory** |
| **Katsumata /2013**^40^ | **Anti-U1 RNP**  **Anti-70Kd RNP**  **Serum** | ***** | ***** | ***** | *****  **Appropriate measurement (assay) of the analyte levels but no further description (Se, Sp, CV)** | ***** | *****  **Define as ACR, but no mentioned of which syndroms** | ***** | **7/10** | **Good** |
| **Kwiecinski/ 2009**^41^ | **Urokinase plasminogen activator (uPA),**  **tissue plasminogen activator (tPA), D-dimer, and plas-**  **minogen activator inhibitor 1 (PAI-1)**  **CSF** | ***** | ***** | ***** | ****** | ***** | ****** | ***** | **9/10** | **Very good** |
| **Labrador-Horrillo/**  **2012**^42^ | **Anti-ganglioside antibodies (AGA) [GM1, GM2, GM3, asialo-GM1 GD1a, GD1b,**  **GD3, GT1b, GQ1b]**  **Serum** | ***** | ***** | ***** | ****** | **Information was not provided** | ***** | ***** | **7/10** | **Good** |
| **Lapteva/**  **2006**^43^ | **Anti-NR2**  **Serum** | ***** | **Not justified** | ***** | ***** | *****  **(Information was not provided, but regression results were adjusted by relevant clinical and demographic features)** | ***** | ***** | **6/10** | **Satisfactory** |
| **Li/**  **2019**^44^ | **Tau protein**  **Phospho-Tau protein**  **DJ-1 protein**  **Nerve**  **growth factor (NGF)**  **α-synuclein (α-SYN)**  **ubiquitin carboxyl hydrolase L1 (UCH-L1)**  **Anti- UCH-L1**  **CSF**  **Serum (a proportion of patient)** | **No description of the derivation of the included subjects** | **Not justified** | ***** | ***** | ***** | ***** | ***** | **5/10** | **Satisfactory** |
| **Long/**  **1990**^45^ | **Anti-lymphocyte Ab**  **Serum** | ***** | ***** | ******* | *****  **Appropriate measurement (assay) of the analyte levels but no further description** | ***** | ***** | ***** | **7/10** | **Good** |
| **Lu/**  **2010**^46^ | **Cytokines (IL-1, IL-4, IL-6, IL-10, IL-17, TNF-a,**  **and IFN-g)**  **Chemokines (CCL2/MCP-1, CCL5/**  **RANTES, CXCL8/IL-8, CXCL10/IP-10)**  **Adhesion molecules (VCAM-1, P-selectin)**  **CSF** | ***** | **No description** | ***** | *****  **Appropriate measurement (assay) of the analyte levels but no further description (limit of detection, CV, etc.)** | ***** | **No description** | ***** | **4/10** | **Unsatisfactory** |
| **Lu/**  **2010**^47^ | **Anti-INA (Internexin)**  **CSF**  **Serum** | ***** | **No description** | *****  **Appropriate measurement (assay) of the analyte levels but no further description (limit of detection, CV, etc.)** | ***** | ***** | *****  **No description, but patients were classified**  **according to the ACR nomenclature and case definitions** | ***** | **6/10** | **Satisfactory** |
| **Mader/**  **2018**^48^ | **IgG anti-DWEYS**  **Anti-4 (AQP-4)**  **Anti- myelin oligoden-**  **drocyte glycoprotein (MOG)**  **Serum** | **No description** | **No description** | ***** | ***** | **No description** | *****  **No description, but patients were classified**  **according to the ACR nomenclature and case definitions** | ***** | **4/10** | **Unsatisfactory** |
| **Magro-Checa /2016**^49^ | **anti-C1q**  **C1q CIC**  **AP50**  **CH50**  **C3**  **C4**  **C1q**  **Serum** | ***** | ***** | ***** | *****  **Appropriate measurement (assay) of the analyte levels but no further description (limit of detection, CV, etc.)** | ***** | *****  **No description, but patients were classified**  **according to the ACR nomenclature and case definitions** | ***** | **7/10** | **Good** |
| **Massardo/ 2015**^50^ | **Anti-NRD2**  **Anti-Ribo P**  **Serum** | ***** | **No description** | ***** | *****  **Appropriate measurement (assay) of the analyte levels but no further description (limit of detection, CV, etc.)** | **No description** | ***** | ***** | **5/10** | **Satisfactory** |
| **Matsueda /2018**^51^ | **Anti-GRP78**  **(Glucose-regulated protein 78)**  **Serum** | ***** | **No description** | ***** | ****** | **No description** | ***** | ***** | **6/10** | **Satisfactory** |
| **Monahan /2022**^52^ | **anti-PTM [Post-translationally modified proteins] IgG**  **(MAA, AGE, CarP, Cit,**  **AL and NT)**  **Serum** | ***** | **No description** | ***** | ****** | ***** | ****** | ***** | **8/10** | **Good** |
| **Munoz-Grajales/**  **2024**^53^ | **IL-10, IL-6, IFN-g, TNF-a, TWEAK,**  **S100B, S100A8/A9, NGAL**  **MMP-9**  **Serum** | ***** | **No description** | ***** | ****** | ****** | ****** | ***** | **9/10** | **Very good** |
| **Noris-Garcia /2018**^54^ | **S100B**  **Brain-derived neurotrophic factor (BNDF)**  **Serum** | **No description of the derivation of the included subjects** | **Not justified** | **No description of the response rate or the characteristics of the responders and the non-responders** | *****  **Appropriate measurement (assay) of the analyte levels but no further description** | **Information was not provided** | ****** | ***** | **4/10** | **Unsatisfactory** |
| **Ogawa/**  **2016**^55^ | **IgG antibodies against mNR1 and four preparations of 25-AA**  **peptides (NR1-A, B, C, and D)=mNR1 and various NR1**  **peptides**  **Serum**  **CSF** | **No description** | **No description** | ***** | ****** | **No description** | *****  **No description, but patients were classified**  **according to the ACR nomenclature and case definitions** | ***** | **5/10** | **Satisfactory** |
| **Pang/**  **2024**^56^ | **BAFF**  **IL-6**  **TNF-a**  **IL-10**  **Serum** | ***** | **No description** | ***** | ***** | ***** | *****  **No description, but patients were classified**  **according to the ACR nomenclature and case definitions** | ***** | **6/10** | **Satisfactory** |
| **Pereira/**  **1992**^57^ | **Anti-Ganglioside antibodies (AGA) IgG and IgM**  **Serum**  **CSF** | **No description of the derivation of the included subjects** | **Not justified** | ***** | *****  **Appropriate measurement (assay) of the analyte levels but no further description (limit of detection, CV, etc.)** | ***** | **No description** | ***** | **4/10** | **Unsatisfactory** |
| **Robbins/**  **1988**^58^ | **ANFA (**  **Anti neurofilament antibody)**  **Serum** | ***** | **No description** | ***** | ***** | **No description** | *****  **NPSLE was clinically diagnosed**  **using the criteria of How** | ***** | **5/10** | **Satisfactory** |
| **Sanna/**  **2003**^59^ | **aPL**  **Serum** | ***** | ***** | ***** | ***** | ***** | ***** | ***** | **7/10** | **Good** |
| **Sato/**  **2010**^60^ | **Anti–U1 RNP**  **Serum**  **CSF** | **No** | **No description** | ***** | ***** | **No description (comparators groups were central vs no central NPSLE)** | ***** | ***** | **4/10** | **Satisfactory** |
| **Schenatto/ 2006**^61^ | **S100B**  **Serum** | **No description** | **No description** | ***** | ***** | ***** | ***** | ***** | **5/10** | **Satisfactory** |
| **Schneebaum/ 1991**^62^ | **Anti-RiboP**  **Serum** | ***** | **No description** | ***** | ***** | **No description** | ***** | ***** | **5/10** | **Satisfactory** |
| **Seth/**  **2020**^63^ | **C3 and C4**  **IgG and IgM antibodies against**  **aCL and b2GP1**  **LA test**  **ENAs**  **Antibodies against**  **the myelin antigens [anti-myelin anti-**  **body (AMA), anti–myelin oligodendrocyte glyco-**  **protein (MOG)**  **and anti–myelin-associated**  **glycoprotein antibody (MAG)**  **Serum** | ***** | ***** | **No all patient anti-Myeline** | ***** | ***** | ***** | ***** | **6/10** | **Satisfactory** |
| **Shi/**  **2023**^64^ | **IL-6**  **Serum**  **CSF** | ***** | ***** | ***** | ***** | ***** | ***** | ***** | **7/10** | **Good** |
| **Sun/**  **2019**^65^ | **Anti-GAPDH Autoantibody**  **Serum** | **No description** | **No description** | ***** | ***** | **No description** | ***** | ***** | **4/10** | **Unsatisfactory** |
| **Svenungsson/ 2001**^66^ | **ON metabolites (CSF)**  **PBMC and CSF (TNFa, IFNg, IL-10, IL-4)** | **No** | **No description** | ***** | ***** | ****** | ****** | ***** | **7/10** | **Good** |
| **Taha/**  **2022**^67^ | **Anti-Sm**  **Anti-dsDNA**  **Anti-histone**  **Anti-nucleosome**  **Anti-ribosomal p**  **Anti RO52**  **Anti SS-B (? 52 and 60)**  **Anti PNCA Abs**  **Anti-Centromere B Abs**  **Anti SS-A**  **Serum** | ***** | **Not justified** | **No description of the response rate or the characteristics of the responders and the non-responders** | *****  **Appropriate measurement (assay) of the analyte levels but no further description** | *****  **By design (matched by age, sex, and**  **level of education, but no treatment)** | *****  **Presence of NPSLE manifestation as per record linkage** | ***** | **5/10** | **Satisfactory** |
| **Tjensvoll/**  **2021**^68^ | **Neurofilament light (Nf),, anti-P antibodies, protein S100B, TWEAK and anti-NR2 antibodies**  **CSF** | **No** | **No description** | **No description** | ***** | **No description** | ***** | ***** | **3/10** | **Unsatisfactory** |
| **Toubi/**  **1995**^69^ | **Antiphospholipid**  **Antibodies (aCL and LA)**  **Serum/plasma** | ***** | ***** | **No clear** | ***** | **No description** | ***** | ***** | **5/10** | **Satisfactory** |
| **Trysberg/**  **2003**^70^ | **Light subunit of the neurofila-**  **ment triplet protein (NFL) and that of glial fibrillary**  **acidic protein (GFAP)**  **CSF** | **No clear** | *** (consecutive)** | ***** | ***** | ***** | ***** | ***** | **6/10** | **Satisfactory** |
| **Trysberg/**  **2004**^71^ | **MMP-2**  **MMP-9**  **(CSF)** | **No clear** | *** (consecutive)** | ***** | ***** | ***** | ***** | ***** | **6/10** | **Satisfactory** |
| **Trysberg/ 2004**^72^ | **Tau, amyloid**  **precursor protein (APP), β-amyloid protein (Aβ42), and**  **transforming growth factor beta (TGF-β)**  **CSF** | **No clear** | *** (consecutive)** | **No measured in all (no clear)** | ***** | ***** | ***** | ***** | **5/10** | **Satisfactory** |
| **Ushigusa/**  **2016**^73^ | **Soluble α-klotho**  **Serum**  **CSF**  **IL-1ra, IL-2, IL-5, IL-6, IL-7, IL-9, IL-**  **10, IL-12(p70), IL-15, IL-17, FGF-basic, GM-CSF, IFN-γ, MCP-1, MIP-1a,**  **and TNF-α=**  **CSF** | **No description of the derivation of the included subjects** | **Not justified** | ***** | ****** | ***** | ***** | ***** | **6/10** | **Satisfactory** |
| **Van der Meulen/**  **2017**^74^ | **Ab against 94bAgs (multiplexed protein array for 94 antigens)**  **Serum** | **No description** | **No description** | ***** | ****** | ***** | ***** | ***** | **6/10** | **Satisfactory** |
| **Vanarsa/2022 ^75^** | **CSF proteomic panel of 1,129 (incl. lipocalin-2, IgM, M-CSF, C3)** | ***** | **No justified** | ***** | ****** | ***** | ***** | ***** | **7/10** | **Good** |
| **Velarde-Mejia/**  **2024**^76^ | **IgM anti-β2 glycoprotein**  **IgG anti-β2 glycoprotein**  **IgM anticardiolipin**  **IgG anticardiolipin**  **Serum**  **Lupus anticoagulant**  **Plasma** | **No description** | ***** | ***** | ***** | ***** | ***** | ***** | **6/10** | **Satisfactory** |
| **Xu-Yang/**  **2008**^77^ | **S100B**  **Serum**  **CSF** | ***** | **No description** | ***** | *****  **Appropriate measurement (assay) of the analyte levels but no further description (limit of detection, CV, etc.)** | ***** | ***** | ***** | **5/10** | **Satisfactory** |
| **Yoshio/**  **2016**^78^ | **IL-6, IL-8, IP-10, MCP-1, gran-**  **ulocyte colony-stimulating factor (G-CSF), granu-**  **locyte-macrophage CSF (GM-CSF), IFN-, IL-1,**  **IL-2, IL-4, IL-5, IL-7, IL-9, IL-10, IL-12 (p70), IL-**  **13, IL-15, IL-17, IL-1 receptor antagonist (IL-1ra),**  **TNF-a, eotaxin, basic fibroblast growth factor (FGF**  **basic), macrophage inflammatory proteins-1b**  **(MIP-1b), MIP-1, platelet-derived growth factor**  **(PDGF), RANTES and vascular endothelial growth**  **factor (VEGF)**  **IFN-a**  **Anti-ribosomal p**  **Serum**  **CSF** | **No description of the derivation of the included subjects** | **Not justified** | ***** | *****  **Appropriate measurement (assay) of the analyte levels but no further description** | ****** | **No description** | ***** | **5/10** | **Satisfactory** |
| **Zervides/**  **2022**^79^ | **S100A8/A9**  **Serum**  **CSF (In a proportion)** | ***** | *** (consecutive)** | ***** | ***** | ****** | ***** | ***** | **8/10** | **Good** |

This scale has been adapted from the Newcastle-Ottawa Quality Assessment Scale for cohort studies to provide a quality assessment of cross-sectional studies.

SELECTION: (Maximum 4 stars)

1. Representativeness of the sample:

a. Truly representative of the average in the target population. * (all subjects or random sampling)

b. Somewhat representative of the average in the target group. * (non-random sampling)

c. Selected group of users/convenience sample.

d. No description of the derivation of the included subjects.

2. Sample size:

a. Justified and satisfactory *

b. Not justified

3. Non-respondents:

a. Comparability between respondents and non-respondents’ characteristics is established, and the response rate is satisfactory (≥90% of patients have the analyte [Ab, cytokine, or molecule] levels available). *

b. The response rate is unsatisfactory, or the comparability between respondents and nonrespondents is unsatisfactory

c. No description of the response rate or the characteristics of the responders and the non-responders.

4. Ascertainment of the exposure (risk factor):

a. Appropriate measurement of the analyte levels (assay) and features/performance of the assay (matrix, CV, Sensitivity, etc.) **

b. Appropriate measurement (assay) of the analyte levels but no further description*

c. Measurement methods not described.

COMPARABILITY: (Maximum 2 stars)

1. Comparability of subjects in different outcome groups (NPSLE and non-NPSLE) based on design or analysis.

a. One star was given if age was comparable between the groups. *

b. One additional star was given if any other variables such as treatment, race, disease activity, disease duration, etc.

were comparable. *

c. Information was not provided, or groups were not comparable.

OUTCOME: (Maximum 3 stars)

a. NPSLE manifestation(s) defined by accepted methods/tests/tools/clinically by expert (when apply)**

b. Presence of NPSLE manifestation as per record linkage *

c. No description.

STATISTICAL TEST: (Maximum 1 star)

a. Statistical test used to analyze the data clearly described, appropriate, and measures of association presented including confidence intervals and probability level (P value). *

b. Statistical test not appropriate, not described, or incomplete.

**Interpreting total stars (Cross-sectional Studies; max = 10)**

**Very Good Studies:** 9-10

**Good Studies:** 7-8

**Satisfactory Studies:** 5-6

**Unsatisfactory Studies:** 0 to 4

**References**

1.	Abdel-Nasser, A. M., Ghaleb, R. M., Mahmoud, J. A., Khairy, W. & Mahmoud, R. M. Association of anti-ribosomal P protein antibodies with neuropsychiatric and other manifestations of systemic lupus erythematosus. *Clin Rheumatol* 27, 1377–1385 (2008).

2.	Abdul-Sattar, A. B., Goda, T. & Negm, M. G. Neuropsychiatric manifestations in a consecutive cohort of systemic lupus erythematosus; a single center study. *Int J Rheum Dis* 16, 715–723 (2013).

3.	Afeltra, A. *et al.* Neuropsychiatric lupus syndromes: relationship with antiphospholipid antibodies. *Neurology* 61, 108–110 (2003).

4.	Ahmadzade, A. *et al.* Correlation between neurofilament, HMGB1, MMP9, ds DNA blood levels and cognitive impairment in patients with neuropsychiatric systemic lupus erythematosus. *Caspian J Intern Med* 15, 58–65 (2024).

5.	Arinuma, Y., Kikuchi, H. & Hirohata, S. Anti-ribosomal P protein antibodies influence mortality of patients with diffuse psychiatric/neuropsychological syndromes in systemic lupus erythematous involving a severe form of the disease. *Mod Rheumatol* 29, 612–618 (2019).

6.	Arinuma, Y. *et al.* Brain MRI in patients with diffuse psychiatric/neuropsychological syndromes in systemic lupus erythematosus. *Lupus Sci Med* 1, e000050 (2014).

7.	Y., A. & T., Y. Association of cerebrospinal fluid anti-NR2 glutamate receptor antibodies with diffuse neuropsychiatric systemic lupus erythematosus. *Arthritis Rheum* 58, 1130–1135 (2008).

8.	Asano, T. *et al.* Evaluation of blood-brain barrier function by quotient alpha2 macroglobulin and its relationship with interleukin-6 and complement component 3 levels in neuropsychiatric systemic lupus erythematosus. *PLoS One* 12, e0186414 (2017).

9.	Balajkova, V. *et al.* Is serum TWEAK a useful biomarker of neuropsychiatric systemic lupus erythematosus?. *Physiol Res* 69, 339–346 (2020).

10.	Baraczka, K., Nekam, K., Pozsonyi, T., Szuts, I. & Ormos, G. Investigation of cytokine (tumor necrosis factor-alpha, interleukin-6, interleukin-10) concentrations in the cerebrospinal fluid of female patients with multiple sclerosis and systemic lupus erythematosus. *Eur J Neurol* 11, 37–42 (2004).

11.	Bluestein, H. G., Williams, G. W. & Steinberg, A. D. Cerebrospinal fluid antibodies to neuronal cells: association with neuropsychiatric manifestations of systemic lupus erythematosus. *Am J Med* 70, 240–246 (1981).

12.	de Sousa, D. C. *et al.* Cognitive dysfunction in systemic lupus erythematosus is associated with disease activity and oxidative stress: a comparative study with rheumatoid arthritis for identifying biomarkers. *BMC Neurosci* 24, 66 (2023).

13.	Dellalibera-Joviliano, R., Dos Reis, M. L., Cunha, F. de Q. & Donadi, E. A. Kinins and cytokines in plasma and cerebrospinal fluid of patients with neuropsychiatric lupus. *J Rheumatol* 30, 485–92 (2003).

14.	Denburg, S. D., Carbotte, R. M., Ginsberg, J. S. & Denburg, J. A. The relationship of antiphospholipid antibodies to cognitive function in patients with systemic lupus erythematosus. *J Int Neuropsychol Soc* 3, 377–386 (1997).

15.	Fujieda, Y. *et al.* Clinical significance of anti‐DNA/N‐methyl‐D‐aspartate receptor 2 antibodies in de novo and post‐steroid cases with neuropsychiatric systemic lupus erythematosus. *Int J Rheum Dis* 22, 443–448 (2019).

16.	George-Chandy, A., Trysberg, E. & Eriksson, K. Raised intrathecal levels of APRIL and BAFF in patients with systemic lupus erythematosus: relationship to neuropsychiatric symptoms. *Arthritis Res Ther* 10, R97 (2008).

17.	Gerli, R. *et al.* Clinical and serological associations of ribosomal P autoantibodies in systemic lupus erythematosus: prospective evaluation in a large cohort of Italian patients. *Rheumatology (Oxford)* 41, 1357–1366 (2002).

18.	Gono, T. *et al.* Anti-NR2A antibody as a predictor for neuropsychiatric systemic lupus erythematosus. *Rheumatology (Oxford)* 50, 1578–1585 (2011).

19.	Guo, Y. *et al.* Utility of autoantibody against an UCH-L1 epitope as a serum diagnostic marker for neuropsychiatric systemic lupus erythematosus. *Clin Exp Rheumatol* https://doi.org/https://dx.doi.org/10.55563/clinexprheumatol/0bjstd (2022) doi:https://dx.doi.org/10.55563/clinexprheumatol/0bjstd.

20.	Hanly, J. G. *et al.* Role of autoantibodies and blood–brain barrier leakage in cognitive impairment in systemic lupus erythematosus. *Lupus Sci Med* 9, e000668 (2022).

21.	Harrison, M. J., Ravdin, L. D. & Lockshin, M. D. Relationship between serum NR2a antibodies and cognitive dysfunction in systemic lupus erythematosus. *Arthritis Rheum* 54, 2515–2522 (2006).

22.	Hawro, T., Bogucki, A., Krupinska-Kun, M., Maurer, M. & Wozniacka, A. Intractable headaches, ischemic stroke, and seizures are linked to the presence of anti-beta2GPI antibodies in patients with systemic lupus erythematosus. *PLoS One* 10, e0119911 (2015).

23.	Herranz, M. T., Rivier, G., Khamashta, M. A., Blaser, K. U. & Hughes, G. R. Association between antiphospholipid antibodies and epilepsy in patients with systemic lupus erythematosus. *Arthritis Rheum* 37, 568–571 (1994).

24.	Hirohata, S., Arinuma, Y., Takayama, M. & Yoshio, T. Association of cerebrospinal fluid anti-ribosomal p protein antibodies with diffuse psychiatric/neuropsychological syndromes in systemic lupus erythematosus. *Arthritis Res Ther* 9, R44 (2007).

25.	Hirohata, S., Arinuma, Y., Yanagida, T. & Yoshio, T. Blood-brain barrier damages and intrathecal synthesis of anti-N-methyl-D-aspartate receptor NR2 antibodies in diffuse psychiatric/neuropsychological syndromes in systemic lupus erythematosus. *Arthritis Res Ther* 16, R77 (2014).

26.	Hirohata, S. *et al.* Accuracy of cerebrospinal fluid IL-6 testing for diagnosis of lupus psychosis. A multicenter retrospective study. *Clin Rheumatol* 28, 1319–1323 (2009).

27.	Hirohata, S. & Kikuchi, H. Role of Serum IL-6 in Neuropsychiatric Systemic lupus Erythematosus. *ACR Open Rheumatol* 3, 42–49 (2021).

28.	Hirohata, S., Sakuma, Y., Matsueda, Y., Arinuma, Y. & Yanagida, T. Role of serum autoantibodies in blood brain barrier damages in neuropsychiatric systemic lupus erythematosus. *Clin Exp Rheumatol* 36, 1003–1007 (2018).

29.	Hirohata, S., Sakuma, Y., Yanagida, T. & Yoshio, T. Association of cerebrospinal fluid anti-Sm antibodies with acute confusional state in systemic lupus erythematosus. *Arthritis Res Ther* 16, 450 (2014).

30.	Hirohata, S. & Tanaka, K. Differential expression of antibodies to NMDA receptor in anti-NMDA receptor encephalitis and in neuropsychiatric systemic lupus erythematosus. *Lupus Sci Med* 6, e000359 (2019).

31.	Hoang, T. T. T. *et al.* Measurement of anti-suprabasin antibodies, multiple cytokines and chemokines as potential predictive biomarkers for neuropsychiatric systemic lupus erythematosus. *Clin Immunol* 237, 108980 (2022).

32.	Hopia, L. *et al.* Cerebrospinal fluid levels of a proliferation-inducing ligand (APRIL) are increased in patients with neuropsychiatric systemic lupus erythematosus. *Scand J Rheumatol* 40, 363–372 (2011).

33.	How, A., Dent, P. B., Liao, S. K. & Denburg, J. A. Antineuronal antibodies in neuropsychiatric systemic lupus erythematosus. *Arthritis Rheum* 28, 789–795 (1985).

34.	Huang, Q. *et al.* Expression of HMGB1 and TLR4 in neuropsychiatric systemic lupus erythematosus patients with seizure disorders. *Ann Transl Med* 8, 9–9 (2020).

35.	Ichinose, K. *et al.* Predictors of clinical outcomes in patients with neuropsychiatric systemic lupus erythematosus. *Cytokine* 79, 31–37 (2016).

36.	Ichinose, K. *et al.* Distinguishing the cerebrospinal fluid cytokine profile in neuropsychiatric systemic lupus erythematosus from other autoimmune neurological diseases. *Clin Immunol* 157, 114–120 (2015).

37.	Iikuni, N. *et al.* Raised monocyte chemotactic protein-1 (MCP-1)/CCL2 in cerebrospinal fluid of patients with neuropsychiatric lupus. *Ann Rheum Dis* 65, 253–256 (2006).

38.	K., I. Association of anti-ribosomal P protein antibodies with neuropsychiatric systemic lupus erythematosus. *Arthritis Rheum* 39, 1483–1490 (1996).

39.	Jonsen, A. *et al.* The heterogeneity of neuropsychiatric systemic lupus erythematosus is reflected in lack of association with cerebrospinal fluid cytokine profiles. *Lupus* 12, 846–850 (2003).

40.	Katsumata, Y. *et al.* Serum antibodies against the 70k polypeptides of the U1 ribonucleoprotein complex are associated with psychiatric syndromes in systemic lupus erythematosus: a retrospective study. *Mod Rheumatol* 23, 71–80 (2013).

41.	Kwiecinski, J. *et al.* Relationship between elevated cerebrospinal fluid levels of plasminogen activator inhibitor 1 and neuronal destruction in patients with neuropsychiatric systemic lupus erythematosus. *Arthritis Rheum* 60, 2094–2101 (2009).

42.	Labrador-Horrillo, M. *et al.* Anti-ganglioside antibodies in patients with systemic lupus erythematosus and neurological manifestations. *Lupus* 21, 611–615 (2012).

43.	Lapteva, L. *et al.* Anti-N-methyl-D-aspartate receptor antibodies, cognitive dysfunction, and depression in systemic lupus erythematosus. *Arthritis Rheum* 54, 2505–2514 (2006).

44.	Li, X. *et al.* The clinical significance of ubiquitin carboxyl hydrolase L1 and its autoantibody in neuropsychiatric systemic lupus erythematosus. *Clin Exp Rheumatol* 37, 474–480 (2019).

45.	Long, A. A., Denburg, S. D., Carbotte, R. M., Singal, D. P. & Denburg, J. A. Serum lymphocytotoxic antibodies and neurocognitive function in systemic lupus erythematosus. *Ann Rheum Dis* 49, 249–253 (1990).

46.	Lu, X. Y. *et al.* Intrathecal cytokine and chemokine profiling in neuropsychiatric lupus or lupus complicated with central nervous system infection. *Lupus* 19, 689–695 (2010).

47.	Lu, X. *et al.* Anti-alpha-internexin autoantibody from neuropsychiatric lupus induce cognitive damage via inhibiting axonal elongation and promote neuron apoptosis. *PLoS One* 5, e11124 (2010).

48.	Mader, S. *et al.* Understanding the Antibody Repertoire in Neuropsychiatric Systemic Lupus Erythematosus and Neuromyelitis Optica Spectrum Disorder: Do They Share Common Targets?. *Arthritis Rheumatol* 70, 277–286 (2018).

49.	Magro-Checa, C. *et al.* Complement levels and anti-C1q autoantibodies in patients with neuropsychiatric systemic lupus erythematosus. *Lupus* 25, 878–888 (2016).

50.	Massardo, L. *et al.* Anti-N-methyl-D-aspartate receptor and anti-ribosomal-P autoantibodies contribute to cognitive dysfunction in systemic lupus erythematosus. *Lupus* 24, 558–568 (2015).

51.	Matsueda, Y., Arinuma, Y., Nagai, T. & Hirohata, S. Elevation of serum anti-glucose-regulated protein 78 antibodies in neuropsychiatric systemic lupus erythematosus. *Lupus Sci Med* 5, e000281 (2018).

52.	Monahan, R. C. *et al.* Autoantibodies against specific post-translationally modified proteins are present in patients with lupus and associate with major neuropsychiatric manifestations. *RMD Open* 8, (2022).

53.	Muñoz-Grajales, C. *et al.* Serum S100A8/A9 and MMP-9 levels are elevated in systemic lupus erythematosus patients with cognitive impairment. *Front Immunol* 14, (2024).

54.	Noris-García, E. *et al.* Peripheral levels of brain-derived neurotrophic factor and S100B in neuropsychiatric systemic lupus erythematous. *Lupus* 27, 2041–2049 (2018).

55.	Ogawa, E., Nagai, T., Sakuma, Y., Arinuma, Y. & Hirohata, S. Association of antibodies to the NR1 subunit of N-methyl-D-aspartate receptors with neuropsychiatric systemic lupus erythematosus. *Mod Rheumatol* 26, 377–383 (2016).

56.	Pang, J. *et al.* Correlation Between B-Cell Activating Factor of the Tumor Necrosis Factor Family Level in Serum and Immune Inflammation in Patients with Neuropsychiatric Systemic Lupus Erythematosus and its Clinical Value. *Immunol Invest* 53, 559–573 (2024).

57.	Pereira, R. M., Yoshinari, N. H., De Oliveira, R. M. & Cossermelli, W. Antiganglioside antibodies in patients with neuropsychiatric systemic lupus erythematosus. *Lupus* 1, 175–179 (1992).

58.	Robbins, M. L. *et al.* Antineurofilament antibody evaluation in neuropsychiatric systemic lupus erythematosus. Combination with anticardiolipin antibody assay and magnetic resonance imaging. *Arthritis Rheum* 31, 623–631 (1988).

59.	Sanna, G. *et al.* Neuropsychiatric manifestations in systemic lupus erythematosus: prevalence and association with antiphospholipid antibodies. *J Rheumatol* 30, 985–992 (2003).

60.	Sato, T. *et al.* Anti-U1 RNP antibodies in cerebrospinal fluid are associated with central neuropsychiatric manifestations in systemic lupus erythematosus and mixed connective tissue disease. *Arthritis Rheum* 62, 3730–3740 (2010).

61.	Schenatto, C. B. *et al.* Raised serum S100B protein levels in neuropsychiatric lupus. *Ann Rheum Dis* 65, 829–831 (2006).

62.	Schneebaum, A. B. *et al.* Association of psychiatric manifestations with antibodies to ribosomal P proteins in systemic lupus erythematosus. *Am J Med* 90, 54–62 (1991).

63.	Seth, G. *et al.* Immunological biomarkers in neuropsychiatric systemic lupus erythematosus: a comparative cross-sectional study from a tertiary care center in South India. *Lupus* 29, 413–420 (2020).

64.	Shi, L. *et al.* Performance of eye sign combined with increased interleukin‐6 in cerebrospinal fluid in patients with neuropsychiatric lupus erythematosus. *Int J Rheum Dis* 26, 1464–1473 (2023).

65.	Sun, J. *et al.* Anti-GAPDH Autoantibody Is Associated with Increased Disease Activity and Intracranial Pressure in Systemic Lupus Erythematosus. *J Immunol Res* 2019, 7430780 (2019).

66.	Svenungsson, E. *et al.* Increased levels of proinflammatory cytokines and nitric oxide metabolites in neuropsychiatric lupus erythematosus. *Ann Rheum Dis* 60, 372–379 (2001).

67.	Taha, Z. I. *et al.* Anti-Ribosomal-P Antibody Association with Neuropsychiatric Lupus in Sudanese Patients Attending Rheumatology Clinic in Omdurman Military Hospital. *Open Access Rheumatol* 14, 281–289 (2022).

68.	Tjensvoll, A. B. *et al.* Neurofilament light is a biomarker of brain involvement in lupus and primary Sjogren’s syndrome. *J Neurol* 268, 1385–1394 (2021).

69.	Toubi, E., Khamashta, M. A., Panarra, A. & Hughes, G. R. Association of antiphospholipid antibodies with central nervous system disease in systemic lupus erythematosus. *Am J Med* 99, 397–401 (1995).

70.	Trysberg, E., Nylen, K., Rosengren, L. E. & Tarkowski, A. Neuronal and astrocytic damage in systemic lupus erythematosus patients with central nervous system involvement. *Arthritis Rheum* 48, 2881–2887 (2003).

71.	Trysberg, E., Blennow, K., Zachrisson, O. & Tarkowski, A. Intrathecal levels of matrix metalloproteinases in systemic lupus erythematosus with central nervous system engagement. *Arthritis Res Ther* 6, R551-6 (2004).

72.	Trysberg, E., Höglund, K., Svenungsson, E., Blennow, K. & Tarkowski, A. Decreased levels of soluble amyloid β-protein precursor and β-amyloid protein in cerebrospinal fluid of patients with systemic lupus erythematosus. *Arthritis Res Ther* 6, R129 (2004).

73.	Ushigusa, T. *et al.* Soluble alpha-klotho is a potential biomarker associated with neuropsychiatric systemic lupus erythematosus. *Clin Immunol* 165, 29–34 (2016).

74.	van der Meulen, P. M. *et al.* Protein array autoantibody profiles to determine diagnostic markers for neuropsychiatric systemic lupus erythematosus. *Rheumatology (Oxford)* 56, 1407–1416 (2017).

75.	Vanarsa, K. *et al.* Aptamer‐Based Screen of Neuropsychiatric Lupus Cerebrospinal Fluid Reveals Potential Biomarkers That Overlap With the Choroid Plexus Transcriptome. *Arthritis & Rheumatology* 74, 1223–1234 (2022).

76.	Velarde-Mejía, Y. *et al.* Unveiling the Link Between Antiphospholipid Antibodies and Cognitive Dysfunction in the Almenara Lupus Cohort. *J Clin Rheumatol* 30, 41–45 (2024).

77.	Yang, X., Lin, J., Lu, X. & Zhao, X. Expression of S100B protein levels in serum and cerebrospinal fluid with different forms of neuropsychiatric systemic lupus erythematosus. *Clin Rheumatol* 27, 353–357 (2008).

78.	Yoshio, T. *et al.* IL-6, IL-8, IP-10, MCP-1 and G-CSF are significantly increased in cerebrospinal fluid but not in sera of patients with central neuropsychiatric lupus erythematosus. *Lupus* 25, 997–1003 (2016).

79.	Zervides, K. A. *et al.* Serum S100A8/A9 concentrations are associated with neuropsychiatric involvement in systemic lupus erythematosus: a cross-sectional study. *BMC Rheumatol* 6, 38 (2022).
