# Supplementary material for: The neuropsychiatric systemic lupus erythematosus biomarker landscape: what blood and cerebrospinal fluid analytes have been studied and how they performed? A systematic review and meta-analysis
Source: Front Immunol. 2026 Oct 1;17:1903757. doi: 10.3389/fimmu.2026.1903757 (PMC13668822; doi:10.3389/fimmu.2026.1903757)
Supplement: Supplementary file 3 [file SupplementaryFile3.docx]

Case–control studies quality assessment [Newcastle-Ottawa Scale]

| **First author / year** | **Analyte**  **Matrix** | **Adequate Case definition** | **Representativeness of cases** | **Selection controls** | **Definition controls** | **Comparability cases and controls** | **Ascertainment of exposure** | **Same method ascertainment cases and controls** | **Non-response rate** | **Total score** | **Quality** |
| --- | --- | --- | --- | --- | --- | --- | --- | --- | --- | --- | --- |
| **Fragoso-Loyo / 2012** ^1^ | **IFN-a**  **Serum and CSF** | ***** | ***** | ***** | ***** | ***** | ***** | ***** | ***** | **9/10** | **Good** |
| **Fragoso-Loyo / 2007** ^2^ | **IL-2, IL-4, IL-6, IL-10, TNF-α, IFN-γ, MCP-1, RANTES, IL-8, MIG, IP-10**  **CSF** | ***** | ***** | ***** | ***** | ***** | ***** | ***** | ***** | **9/10** | **Good** |
| **Fragoso-Loyo / 2008** ^3^ |  | ***** | ***** | ***** | ***** | ***** | ***** | ***** | ***** | **9/10** | **Good** |

Coding for case-control studies

SELECTION (Maximum 4 starts)

1) Is the Case Definition Adequate?

a. Requires some independent validation (e.g. >1 person/record/time/process to extract information, or reference to primary record source such as images or medical/hospital records) *

b. Record linkage (e.g. ICD codes in data base) or self-report with no reference to primary record

c. No description

2) Representativeness of the Cases

a. All eligible cases with outcome of interest over a defined period, all cases in a defined catchment area, all cases in a defined hospital or clinic, group of hospitals, health maintenance organisation, or an appropriate sample of those cases (e.g. random sample) *

b. Not satisfying requirements in part(a) or not stated.

3) Selection of Controls

This item assesses whether the control series used in the study is derived from the same population as the cases and essentially would have been cases had the outcome been present.

a. Community controls (i.e. same community as cases and would be cases if had outcome) *

b. Hospital controls, within same community as cases (i.e. not another city) but derived from a hospitalised population

c. No description

4) Definition of Controls

a. If cases are first occurrence of outcome, then it must explicitly state that controls have no history of this outcome. If cases have new (not necessarily first) occurrence of outcome, then controls with previous occurrences of outcome of interest should not be excluded *

b. No mention of history of outcome

COMPARABILITY (Maximum of 2 stars)

Comparability of Cases and Controls based on the design or analysis.

a. *study controls for SLE diagnosis (Select the most important factor.)
b. *study controls for any additional factor(s): disease duration, age, sex (These criteria could be modified to indicate specific control for a second important factor.)

Note:

Either cases and controls must be matched in the design and/or confounders must be adjusted for in the analysis. Statements of no differences between groups or that differences were not statistically significant are not sufficient for establishing comparability.

If the odds ratio for the exposure of interest is adjusted for the confounders listed, then the groups will be comparable on each variable used in the adjustment.

There may be multiple ratings for this item for different categories of exposure (e.g. ever vs. never, current vs. previous or never)

EXPOSURE (A maximum of 3 stars)

1) Ascertainment of exposure
a. *secure record (e.g., surgical records)
b. *structured interview was blind to case/control status

c. Interview not blinded to case/control status
d. Written self-report or medical record only
e. No description

2) Same method of ascertainment for cases and controls

a. *yes

b. no

3) Non-Response rate (dropouts)
a. *same rate for both groups
b. non respondents described
c. rate different and no designation

**Interpreting total stars (Case–Control studies; max = 9)**

**Good quality:** 7-9

**Fair quality:** 5-6

**Poor quality:** 0-4

**Case–control studies (official NOS convention)**

**Good quality:** Selection **3–4*** **and** Comparability **1–2*** **and** Exposure **2–3***

**Fair quality:** Selection **2*** **and** Comparability **1–2*** **and** Exposure **2–3***

**Poor quality:** Selection **0–1*** **or** Comparability **0*** **or** Exposure **0–1***

**References**

1.	Fragoso-Loyo, H., Atisha-Fregoso, Y., Nunez-Alvarez, C. A., Llorente, L. & Sanchez-Guerrero, J. Utility of interferon-alpha as a biomarker in central neuropsychiatric involvement in systemic lupus erythematosus. *J Rheumatol* **39**, 504–509 (2012).

2.	Fragoso-Loyo, H. *et al.* Interleukin-6 and chemokines in the neuropsychiatric manifestations of systemic lupus erythematosus. *Arthritis Rheum* **56**, 1242–1250 (2007).

3.	Fragoso-Loyo, H. *et al.* Serum and cerebrospinal fluid autoantibodies in patients with neuropsychiatric lupus erythematosus. Implications for diagnosis and pathogenesis. *PLoS One* **3**, e3347 (2008).
