# Supplementary material for: The neuropsychiatric systemic lupus erythematosus biomarker landscape: what blood and cerebrospinal fluid analytes have been studied and how they performed? A systematic review and meta-analysis
Source: Front Immunol. 2026 Oct 1;17:1903757. doi: 10.3389/fimmu.2026.1903757 (PMC13668822; doi:10.3389/fimmu.2026.1903757)
Supplement: Supplementary file 4 [file SupplementaryFile4.docx]

Cohort studies quality assessment [ Newcastle-Ottawa Scale]

| **Author /year** | **Analyte and matrix** | **Representativeness of the exposed cohort** | **Selection of the non- exposed cohort** | **Ascertainment of exposure** | **Outcome of interest was not present at start of study** | **Comparability of cohorts based on the design or analysis** | **Assessment of outcome** | **Was follow-up long enough for outcomes to occur** | **Adequacy of follow-up of cohorts** | **Total score** | **Quality** |
| --- | --- | --- | --- | --- | --- | --- | --- | --- | --- | --- | --- |
| **Alarcon-Segovia /1997**^1^ | **APLA=**  **IgG and IgM aCL**  **Anti-B2GPI**  **Serum** | ***** | ***** | ***** | **No description** | ***** | ***** | ***** | ***** | **7/9** | **Good** |
| **Appenzeller /2008**^2^  ***** | **APLA=**  **IgG and IgM aCL**  **LA**  **Serum** | ***** | ***** | ***** | ***** | ***** | ***** | ***** | ***** | **8/9** | **Very good** |
| **Arnett/**  **1996**^3^ | **Anti-Ribo P**  **Serum** | ***** | ***** | ***** | **Could be present** | ***** | ***** | **No description** | **No description** | **5/9** | **Satisfactory** |
| **Bankole/ 2021**^4^  ***** | **aPLA (no specified)**  **Low C3**  **Low C4**  **Anti-Ribo P**  **Anti-Sm**  **Anti-dsDNA**  **Serum** | ***** | ***** | ***** | **Could be present** | ***** | ***** | **No description** | **No description** | **5/9** | **Satisfactory** |
| **Briani/**  **2009**^5^ | **Anti-Ribo P**  **Serum**  **(7 also measured in CSF)** | ***** | ***** | ***** | **Could be present** | ***** | ***** | ***** | ***** | **7/9** | **Good** |
| **Ding/**  **2022**^6^  **CSTAR cohort**  ****** | **Anti-Ribo P**  **Serum** | ***** | ***** | ***** | ***** | ***** | ***** | ***** | ***** | **8/9** | **Very good** |
| **Hanly/**  **2008**^7^  **SLICC Cohort (cross-sectional)** | **LAC**  **IgG aCL**  **Anti-B2GPI**  **Anti-Ribo-P**  **Anti-NR2**  **Serum** | ***** | ***** | ***** | **Present at onset** | ***** | ***** | ***** | ***** | **7/9** | **Good** |
| **Hanly/**  **2011**^8^  **SLICC Cohort**  **(prospective)** | **LAC**  **IgG aCL**  **Anti-B2GPI**  **Anti-Ribo-P**  **Anti-NR2**  **Serum** | ***** | ***** | ***** | ***** | ***** | ***** | ***** | ***** | **8/9** | **Very good** |
| **Hanly/**  **2019**^9^  **SLICC Cohort** | **LAC**  **, serum IgG anticardiolipin, anti–β2-glycoprotein I, anti–ribosomal P (anti-P), and anti–NR2 glutamate receptor antibodies**  **Serum** | ***** | ***** | ***** | **Could be present at onset** | ***** | ***** | ***** | ***** | **7/9** | **Good** |
| **Mikdashi /2005**^10^ | **Anti-Sm**  **Anti-dsDNA**  **C3**  **C4**  **APLA (aCL, LA)**  **Serum** | ***** | ***** | ***** | ***** | ***** | ***** | ***** | **No statement** | **7/9** | **Good** |
| **Mok/**  **2001**^11^ | **APLA=**  **LA**  **IgM and IgM aCL**  **Anti-dsDNA**  **ENA (Ro, La, nRNP, Sm)**  **Serum** | ***** | ***** | ***** | **Could have** | **No specified** | ***** | ***** | ***** | **6/9** | **Good** |

*Retrospective analysis of a cohort

** Look at relationship with neurological damage accrual (longitudinal)

SELECTION (Maximum of 1 star)

1) Representativeness of the exposed cohort

a. Truly representative of the average NPSLE in the community *

b. Somewhat representative of the average NPSLE in the community *

c. Selected group of users eg nurses, volunteers

d. No description of the derivation of the cohort

2) Selection of the non-exposed cohort

a. Drawn from the same community as the exposed cohort *

b. Drawn from a different source

c. No description of the derivation of the non-exposed cohort

3) Ascertainment of exposure

a. Secure record (e.g. Medical records) *

b. Structured interview *

c. Written self-report

d. No description

4) Demonstration that outcome of interest was not present at start of study

a. Yes *

b. No

COMPARABILITY (A maximum of 2 stars)

Comparability of cohorts on the basis of the design or analysis

a. Study controls for age/ethnicity/treatment *

b. Study controls for any additional factor *

OUTCOME (A maximum of 1 star)

1) Assessment of outcome

a. Independent blind assessment *

b. Record linkage *

c. Self-report

d. No description

2) Was follow-up long enough for outcomes to occur

a. Yes *

b. No

3) Adequacy of follow up of cohorts

a. Complete follow up - all subjects accounted for *

b. Subjects lost to follow up unlikely to introduce bias *

c. Follow up rate insufficient (<80 %) and no description of those lost

d. No statement

**Interpreting total stars (Cohort Studies; max = 9)**

**Very Good Studies:** 8-9

**Good Studies:** 6-7

**Satisfactory Studies:** 4-5

**Unsatisfactory Studies:** 0 to 3

**Quality grade (official NOS convention)**

- **Good**: Selection **3–4*and** Comparability **1–2*** **and** Outcome **2–3***
- **Fair**: Selection **2*** **and** Comparability **1–2*** **and** Outcome **2–3***
- **Poor**: Selection **0–1*** **or** Comparability **0*** **or** Outcome **0–1***

**References**

1.	Alarcon-Segovia, D., Estanol, B., Garcia-Ramos, G. & Villa, A. R. Antiphospholipid antibodies and the antiphospholipid syndrome. Clinical relevance in neuropsychiatric systemic lupus erythematosus. *Ann N Y Acad Sci* 823, 279–288 (1997).

2.	Appenzeller, S., Cendes, F. & Costallat, L. T. L. Acute psychosis in systemic lupus erythematosus. *Rheumatol Int* 28, 237–243 (2008).

3.	Arnett, F. C., Reveille, J. D., Moutsopoulos, H. M., Georgescu, L. & Elkon, K. B. Ribosomal P autoantibodies in systemic lupus erythematosus. Frequencies in different ethnic groups and clinical and immunogenetic associations. *Arthritis Rheum* 39, 1833–1839 (1996).

4.	Bankole, A. A., Kazmi, T. R. & Strazanac, A. R. Determination of the Risk Factors Contributing to the Development of Neuropsychiatric Lupus in a Systemic Lupus Erythematosus Cohort. *Cureus* 13, e20129 (2021).

5.	Briani, C. *et al.* Neurolupus is associated with anti-ribosomal P protein antibodies: an inception cohort study. *J Autoimmun* 32, 79–84 (2009).

6.	Ding, Y. *et al.* The role of anti-ribosomal P autoantibodies in the prediction of neuropsychiatric damage in systemic lupus erythematosus based on CSTAR cohort (XIV). *Clin Rheumatol* 41, 1371–1379 (2022).

7.	Hanly, J. G. *et al.* Autoantibodies and neuropsychiatric events at the time of systemic lupus erythematosus diagnosis: results from an international inception cohort study. *Arthritis Rheum* 58, 843–853 (2008).

8.	Hanly, J. G. *et al.* Autoantibodies as biomarkers for the prediction of neuropsychiatric events in systemic lupus erythematosus. *Ann Rheum Dis* 70, 1726–1732 (2011).

9.	Hanly, J. G. *et al.* Psychosis in Systemic Lupus Erythematosus: Results From an International Inception Cohort Study. *Arthritis Rheumatol* 71, 281–289 (2019).

10.	Mikdashi, J., Krumholz, A. & Handwerger, B. Factors at diagnosis predict subsequent occurrence of seizures in systemic lupus erythematosus. *Neurology* 64, 2102–2107 (2005).

11.	Mok, C. C. & Lau, C. S. Neuropsychiatric manifestations and their clinical associations in southern Chinese patients with systemic lupus erythematosus. *Journal of Rheumatology* 28, 766–771 (2001).
