# Supplementary material for: The neuropsychiatric systemic lupus erythematosus biomarker landscape: what blood and cerebrospinal fluid analytes have been studied and how they performed? A systematic review and meta-analysis
Source: Front Immunol. 2026 Oct 1;17:1903757. doi: 10.3389/fimmu.2026.1903757 (PMC13668822; doi:10.3389/fimmu.2026.1903757)
Supplement: Supplementary file 5 [file SupplementaryFile5.docx]

**Supplementary Table 1.** Assay platform, measurement units, positivity thresholds, and derivation of dichotomous odds ratios for studies included in the IgG anticardiolipin meta-analysis.

| **Study** | **Assay platform** | **Units** | **Positivity threshold** | **Method used to derive OR** |
| --- | --- | --- | --- | --- |
| Afeltra et al., 2003^1^ | Commercial ELISA (ORGen Tec) | U/mL | ≥10 U/mL (cut-off above the 99th percentile of controls) | OR reconstructed from reported frequencies (25/44 vs 5/17) |
| Mok et al., 2001^2^ | ELISA | IU/mL | Strongly positive IgG aCL ≥30 IU/mL | OR reconstructed from reported frequencies (16/82 vs 29/317) |
| Jönsen et al., 2003^3^ | ELISA | Not reported | Standard laboratory positivity; numerical cut-off not reported | OR reconstructed from reported frequencies (29/68 vs 25/70) |
| van der Meulen et al., 2017^4^ | Protein microarray | Net Fluorescence Intensity (NFI) | ROC-derived cut-off >0.5 NFI | Published OR reported by the study authors |
| Taha et al., 2022^5^ | ELISA | Not reported | Study laboratory positivity; threshold not reported | OR reconstructed from reported frequencies (8/30 vs 0/60) |
| Monahan et al., 2022^6^ | ELISA | Not reported | Routine clinical laboratory positivity; numerical threshold not reported | OR reconstructed from reported frequencies (21/104 vs 28/245) |

**Abbreviations:** ELISA, enzyme-linked immunosorbent assay; IU, international units; U, arbitrary units; NFI, net fluorescent intensity; OR, odds ratio.

1.	Afeltra, A. *et al.* Neuropsychiatric lupus syndromes: relationship with antiphospholipid antibodies. *Neurology* 61, 108–110 (2003).

2.	Mok, C. C. & Lau, C. S. Neuropsychiatric manifestations and their clinical associations in southern Chinese patients with systemic lupus erythematosus. *Journal of Rheumatology* 28, 766–771 (2001).

3.	Jonsen, A. *et al.* The heterogeneity of neuropsychiatric systemic lupus erythematosus is reflected in lack of association with cerebrospinal fluid cytokine profiles. *Lupus* 12, 846–850 (2003).

4.	van der Meulen, P. M. *et al.* Protein array autoantibody profiles to determine diagnostic markers for neuropsychiatric systemic lupus erythematosus. *Rheumatology (Oxford)* 56, 1407–1416 (2017).

5.	Taha, Z. I. *et al.* Anti-Ribosomal-P Antibody Association with Neuropsychiatric Lupus in Sudanese Patients Attending Rheumatology Clinic in Omdurman Military Hospital. *Open Access Rheumatol.* 14, 281–289 (2022).

6.	Monahan, R. C. *et al.* Autoantibodies against specific post-translationally modified proteins are present in patients with lupus and associate with major neuropsychiatric manifestations. *RMD Open* 8, (2022).
